# Supplementary figures and images for: The Relationship Between the Global Burden of Influenza From 2017 to 2019 and COVID-19: Descriptive Epidemiological Assessment
Source: JMIR Public Health Surveill. 2021 Mar 2;7(3):e24696. doi: 10.2196/24696 (PMC7927952; doi:10.2196/24696)

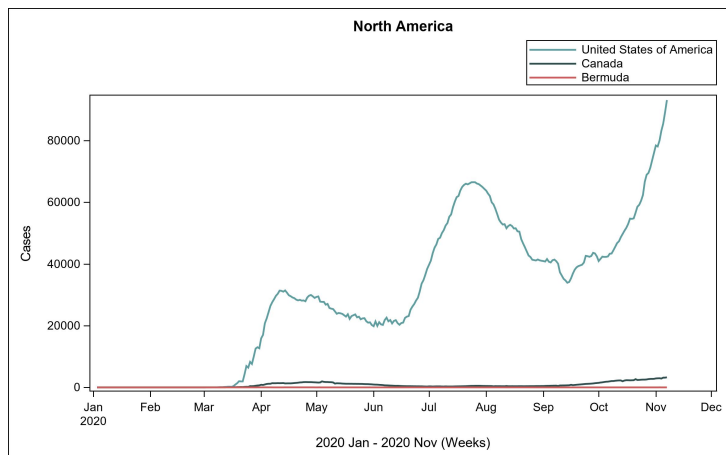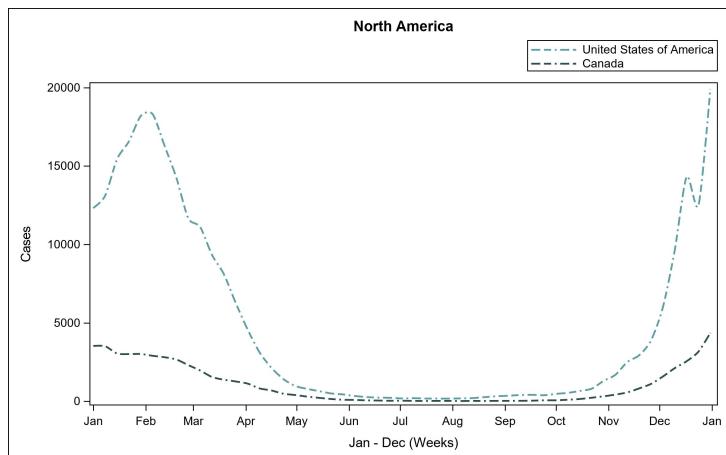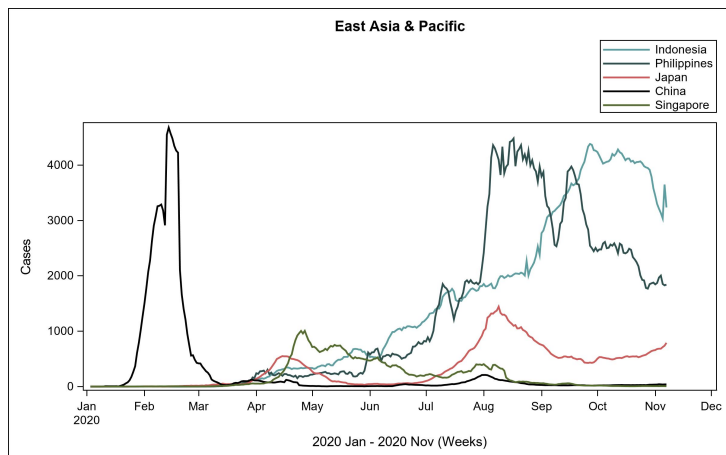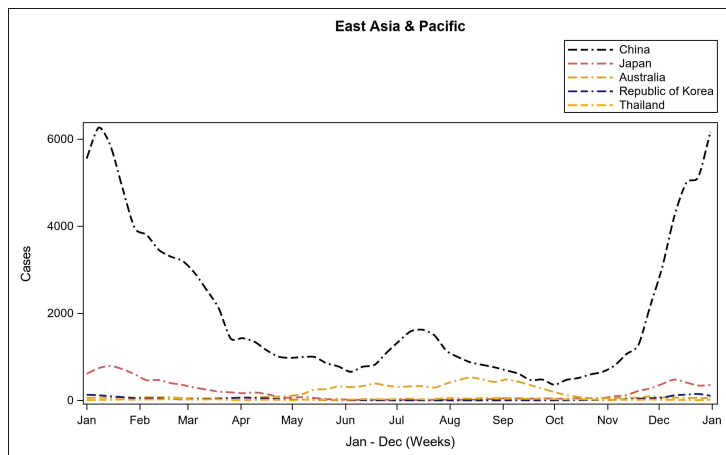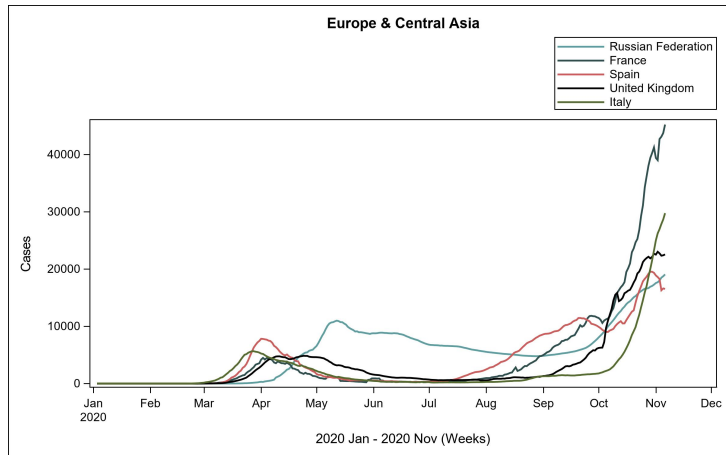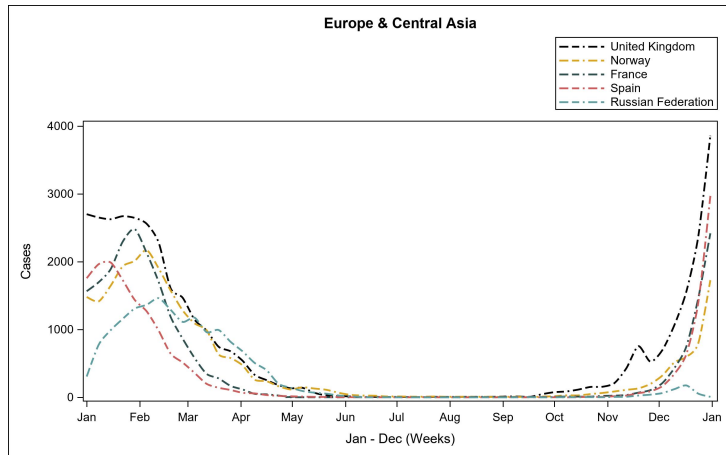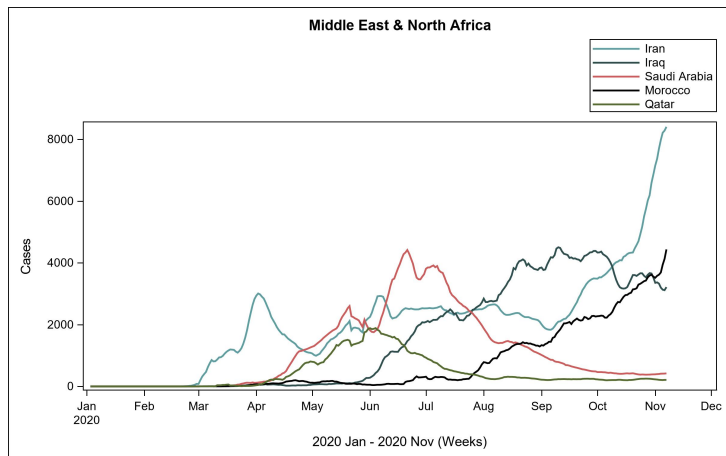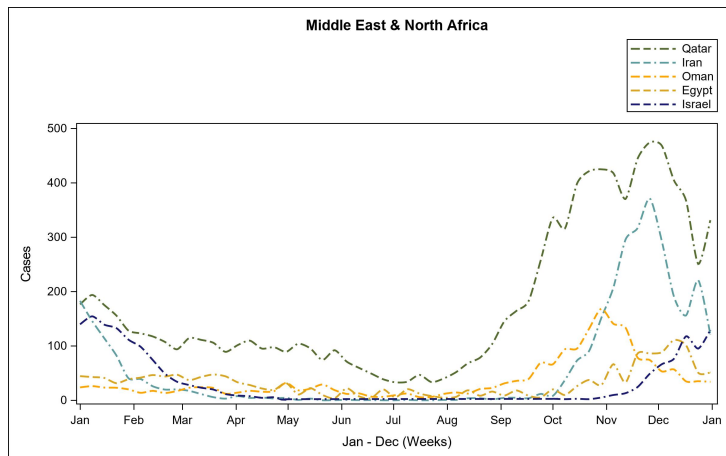

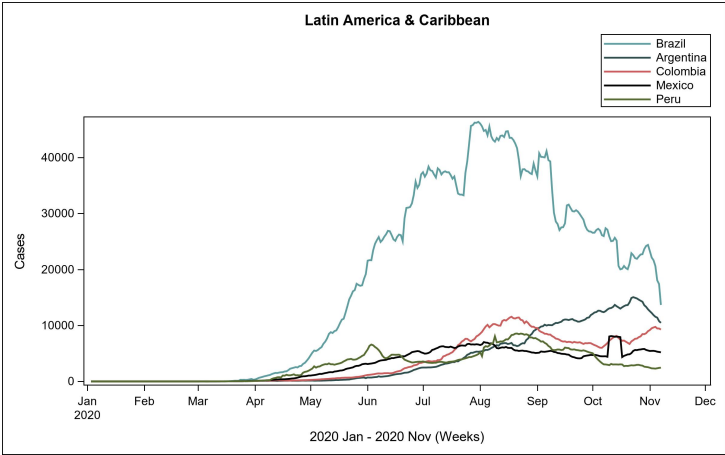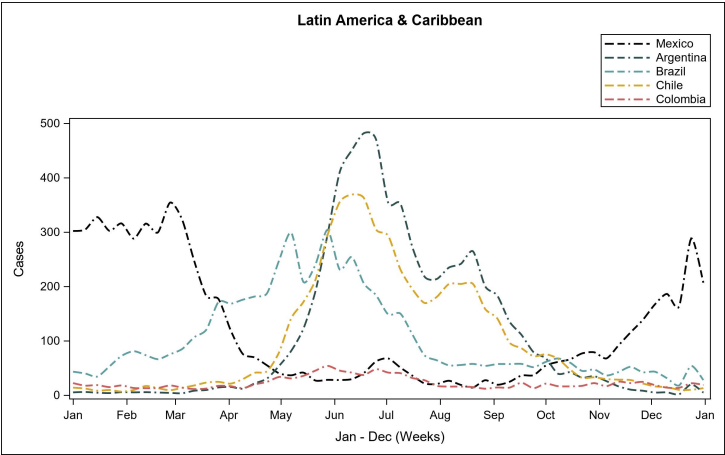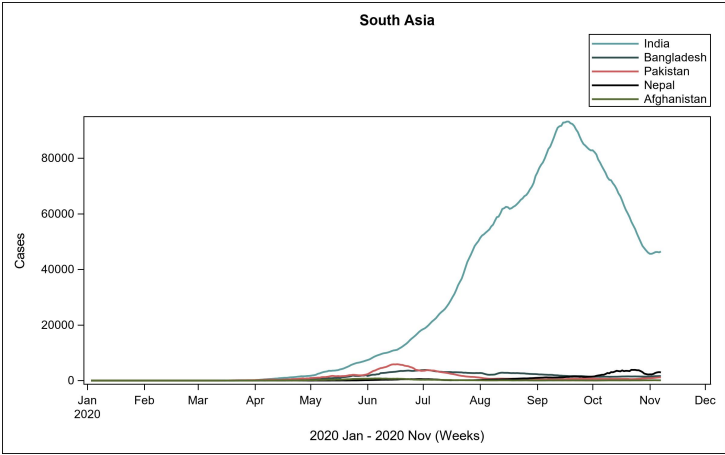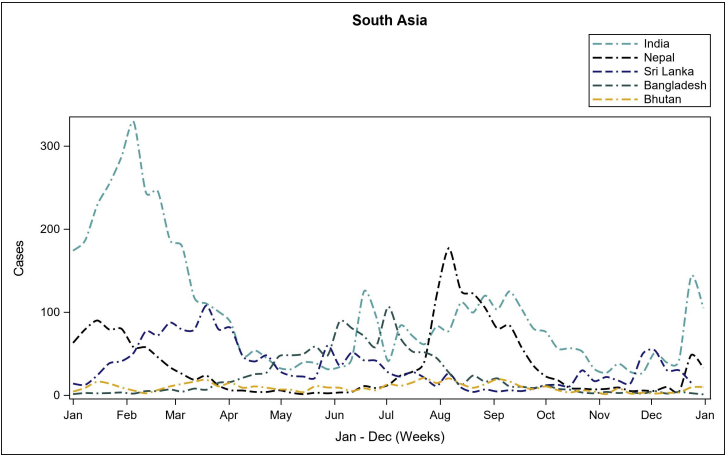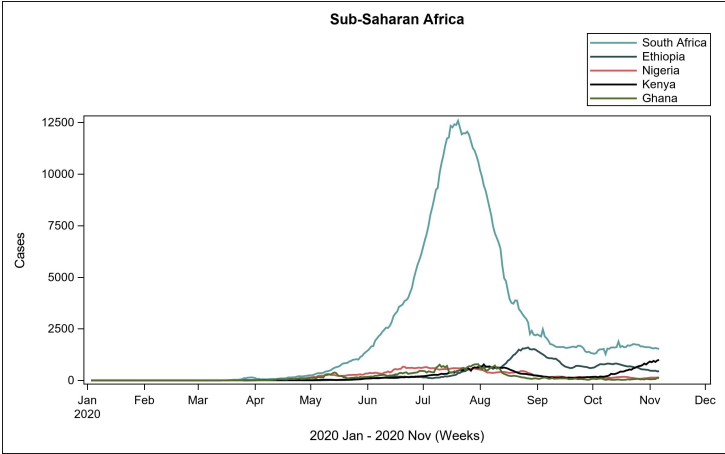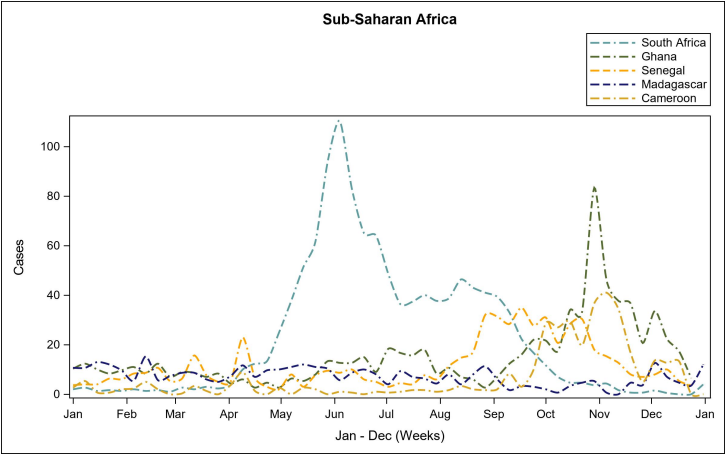

Supplement: Multimedia Appendix 2 [file publichealth_v7i3e24696_app2.pdf]

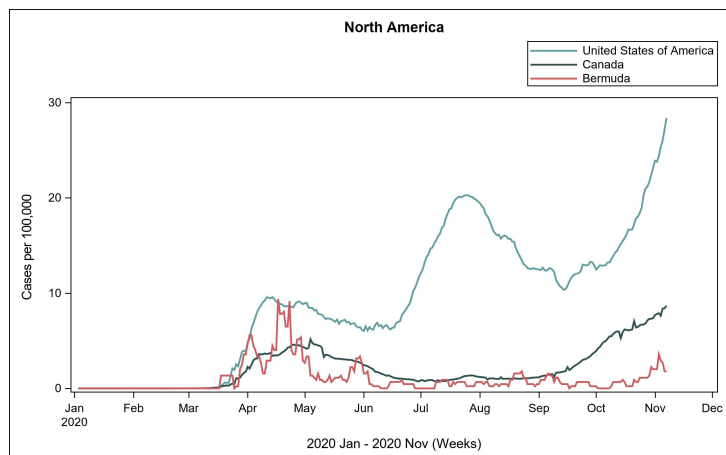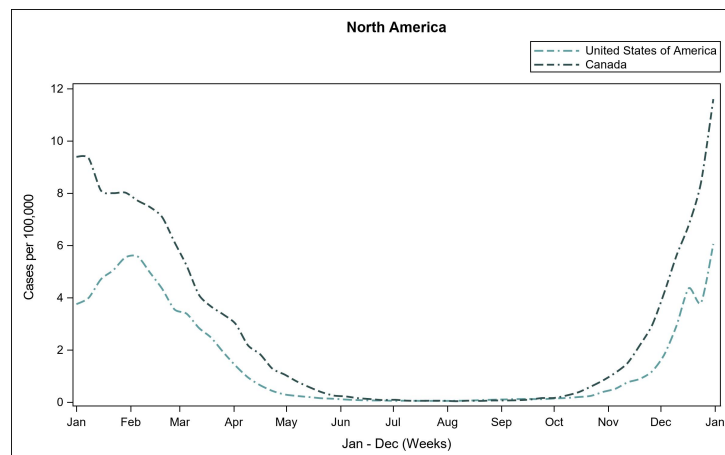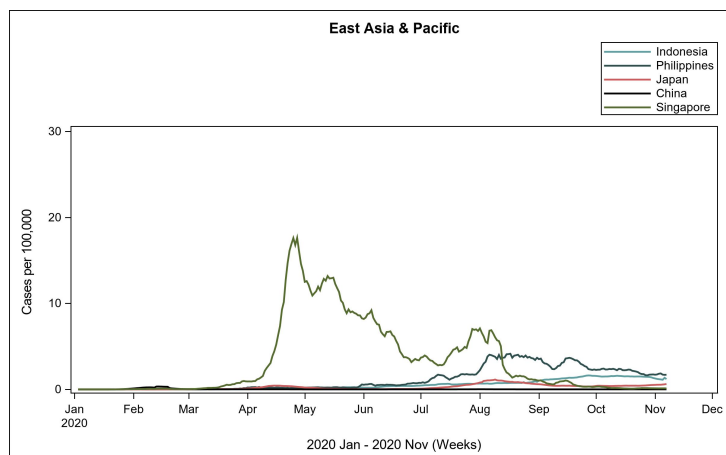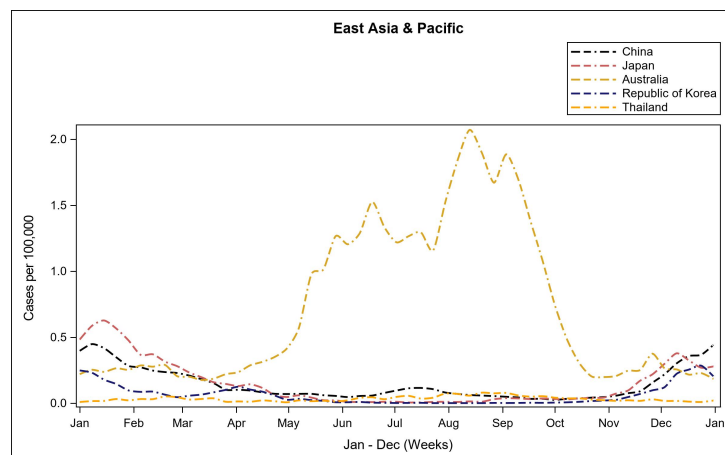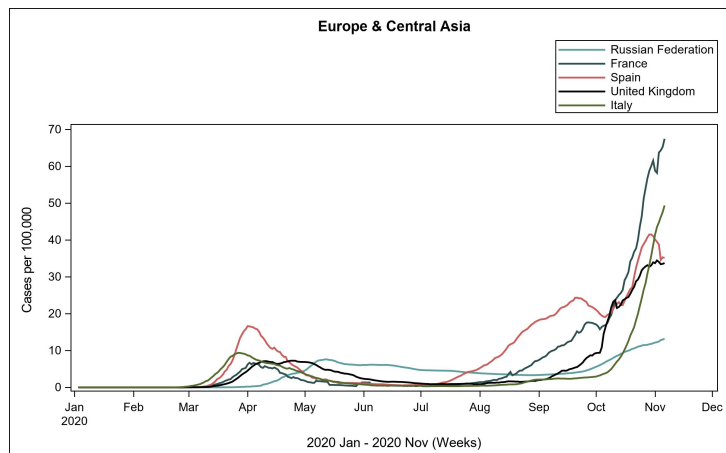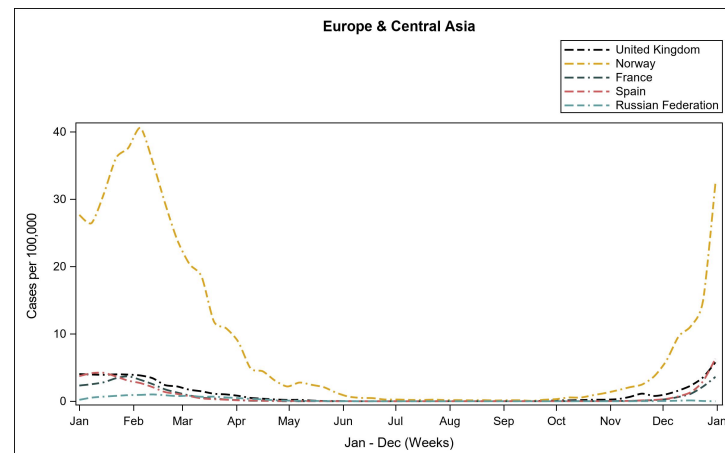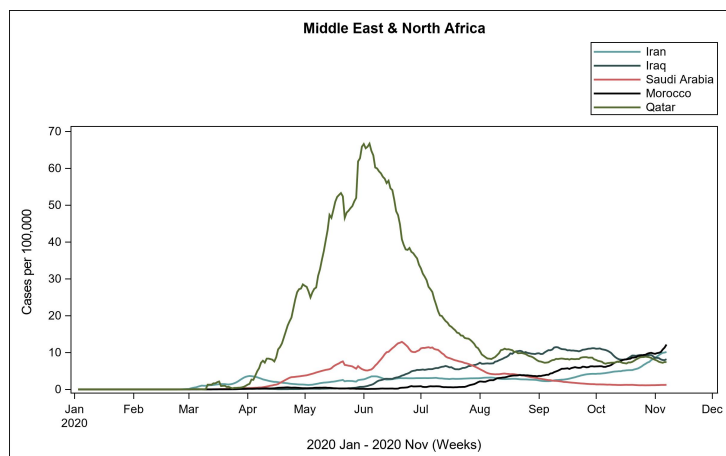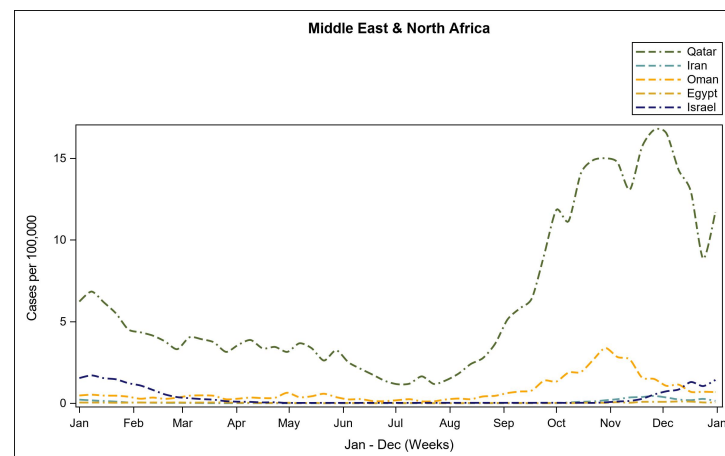

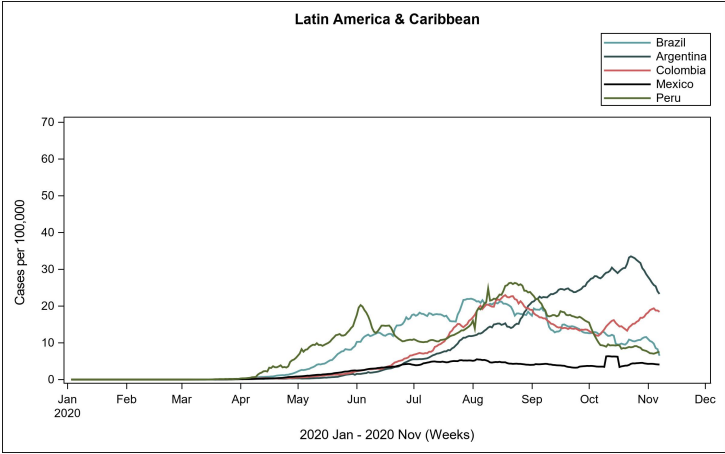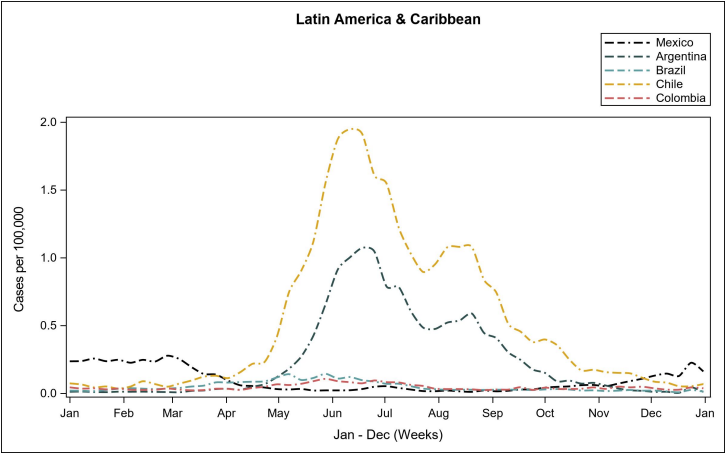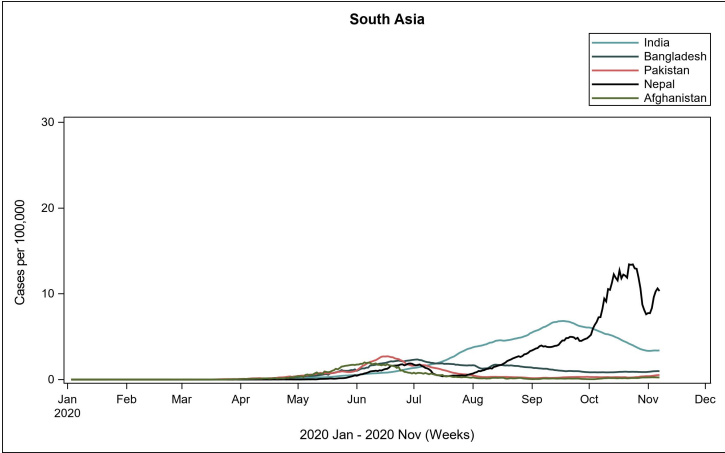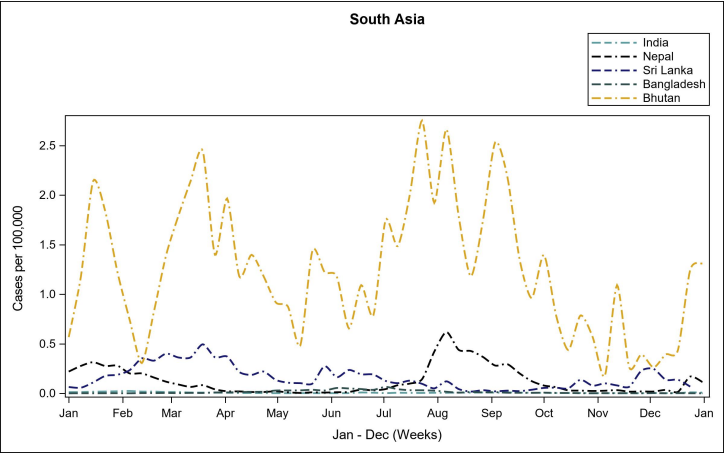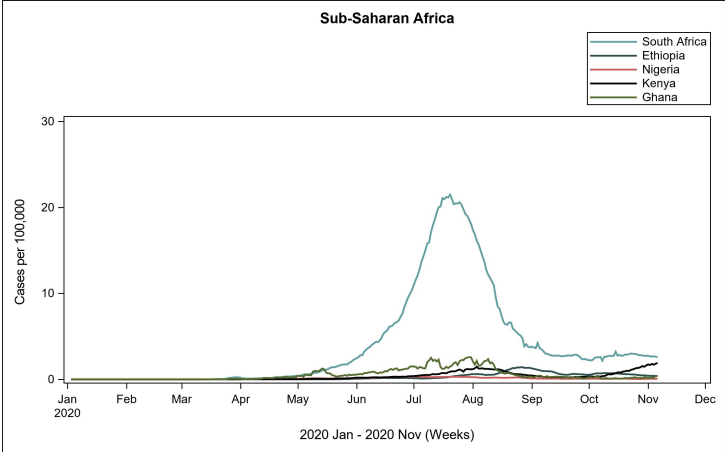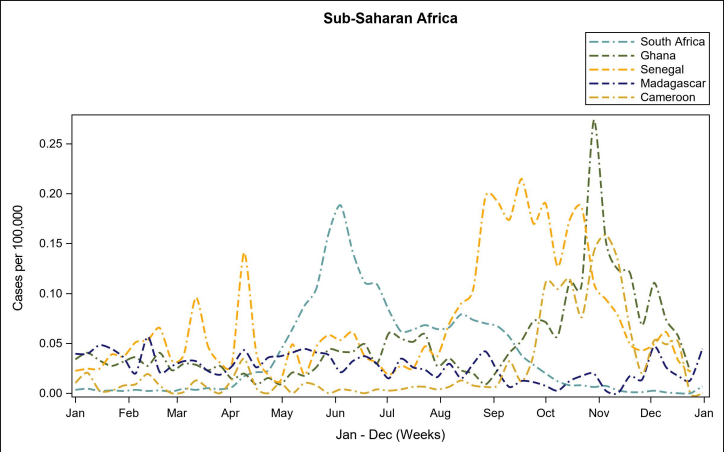

Supplement: Multimedia Appendix 3 [file publichealth_v7i3e24696_app3.pdf]

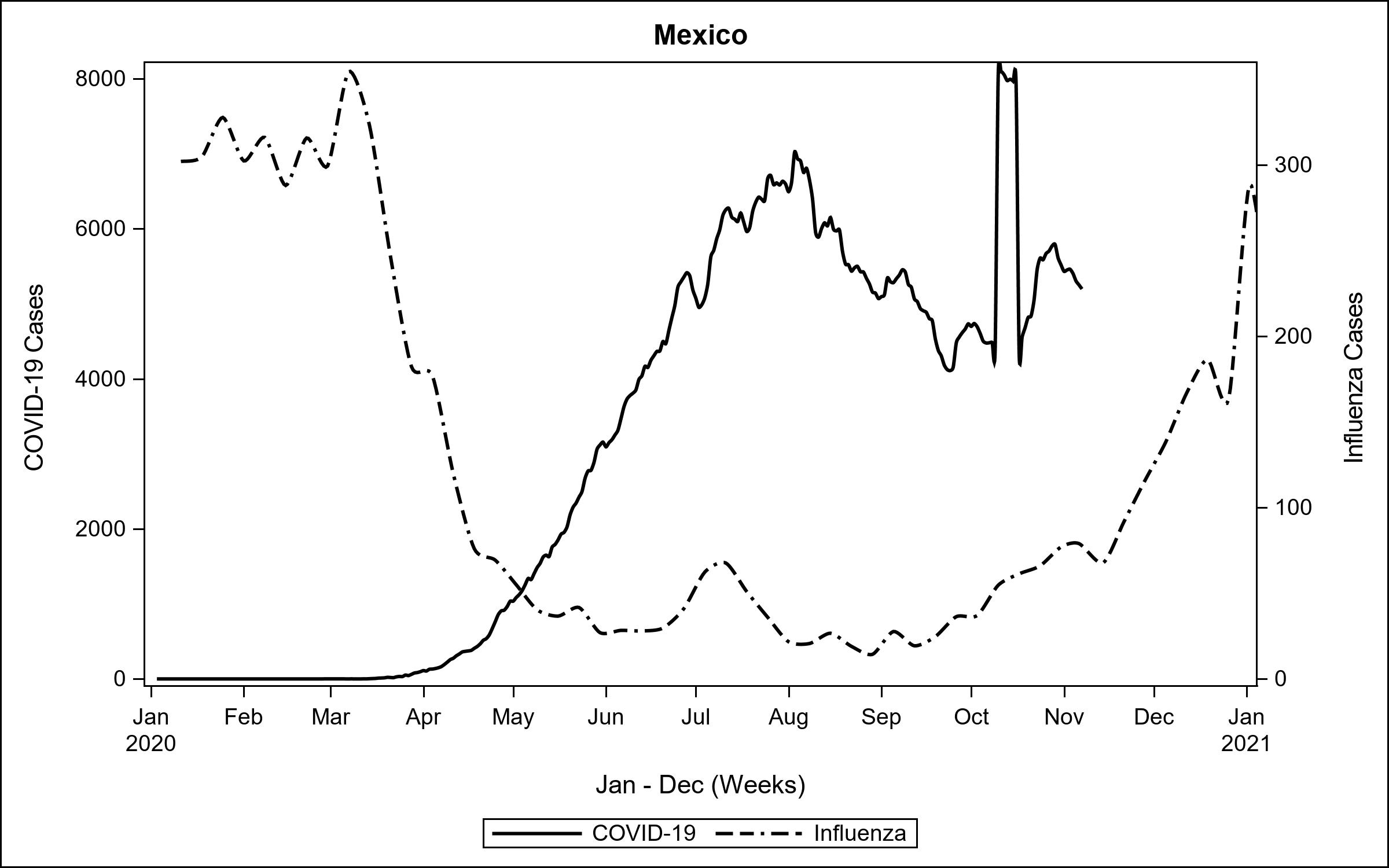

Supplement: Multimedia Appendix 4 [file publichealth_v7i3e24696_app4.zip › Country comparisons_all/Mexico1.jpeg]

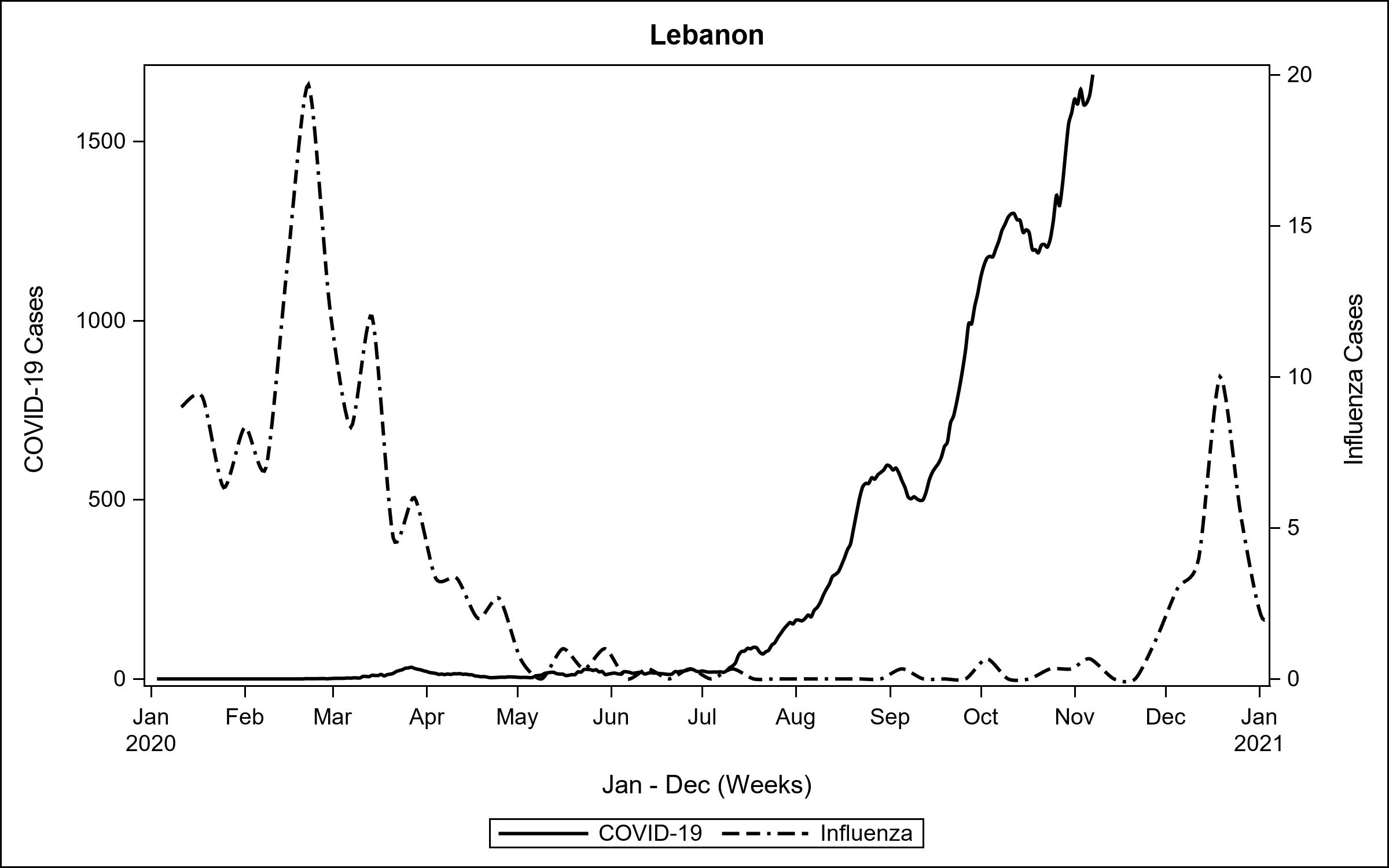

Supplement: Multimedia Appendix 4 [file publichealth_v7i3e24696_app4.zip › Country comparisons_all/Lebanon1.jpeg]

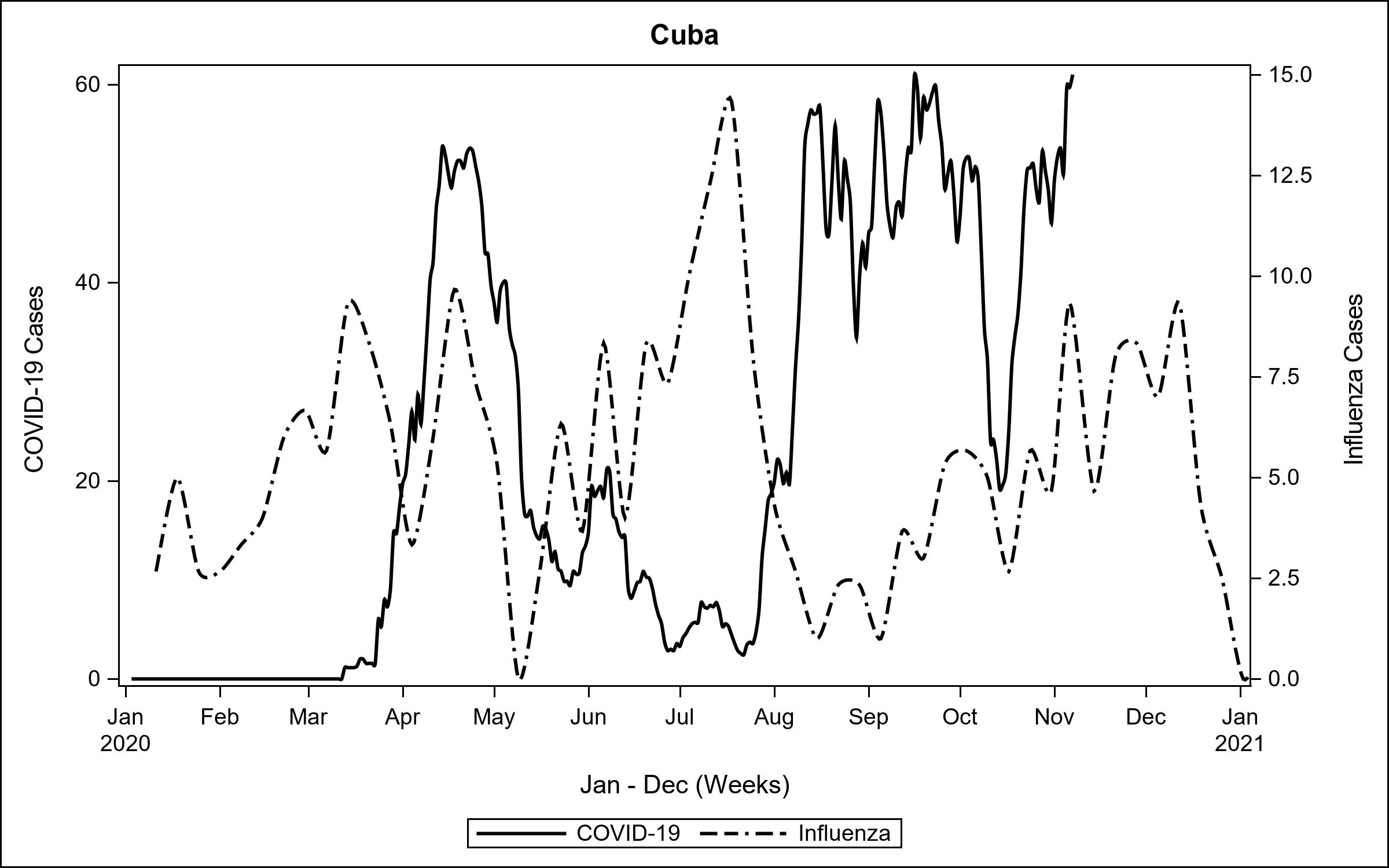

Supplement: Multimedia Appendix 4 [file publichealth_v7i3e24696_app4.zip › Country comparisons_all/Cuba1.jpeg]

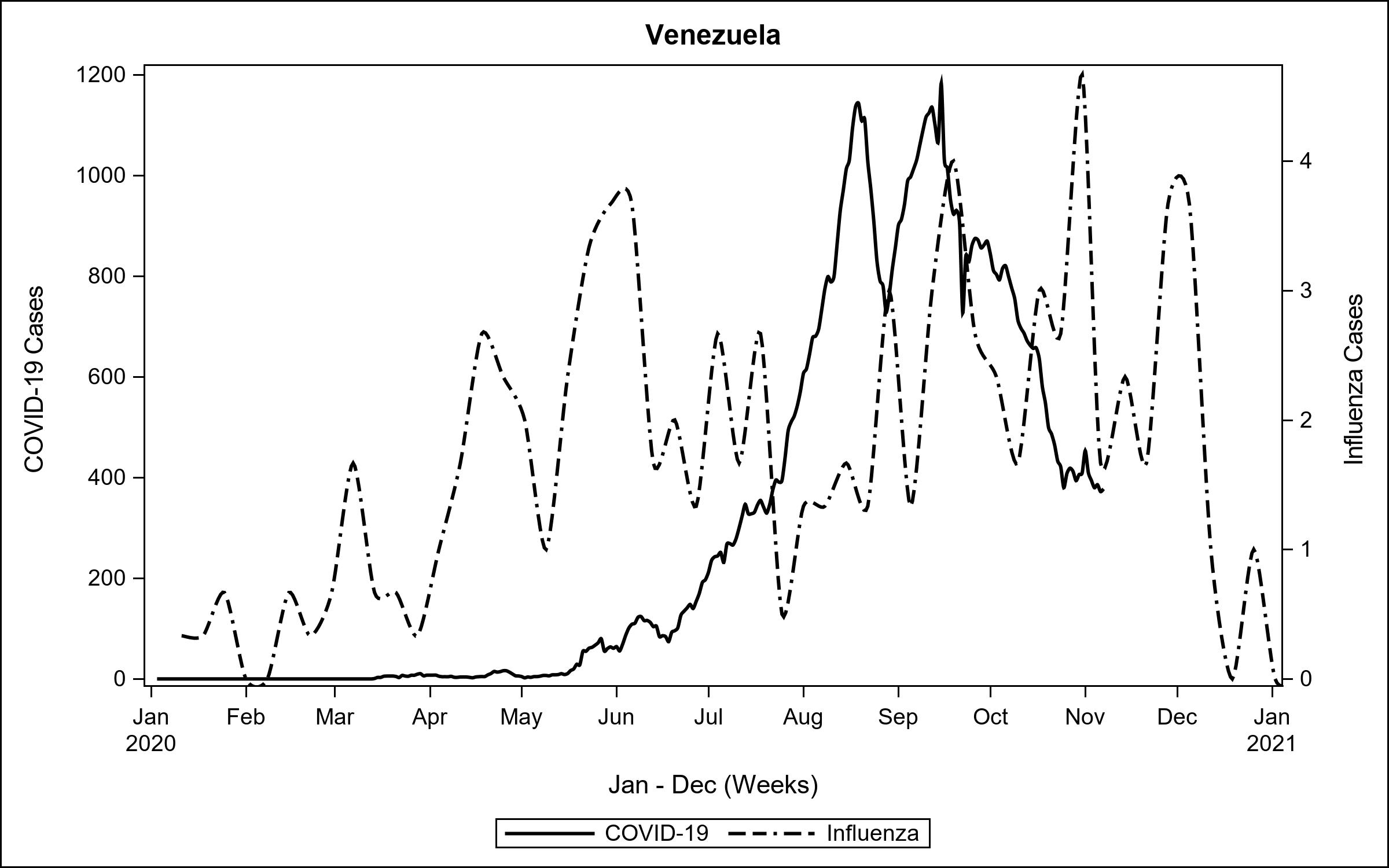

Supplement: Multimedia Appendix 4 [file publichealth_v7i3e24696_app4.zip › Country comparisons_all/Venezuela1.jpeg]

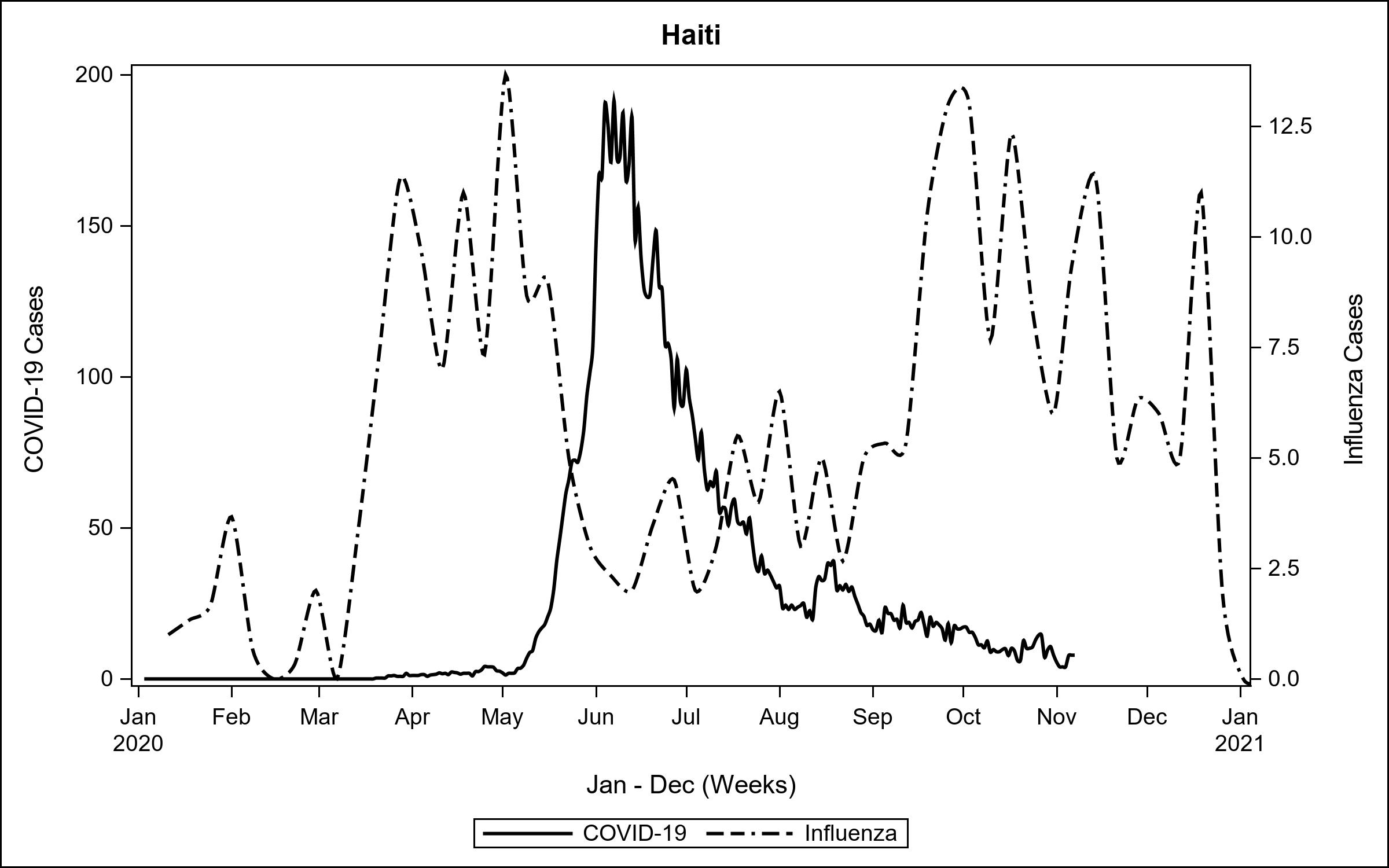

Supplement: Multimedia Appendix 4 [file publichealth_v7i3e24696_app4.zip › Country comparisons_all/Haiti1.jpeg]

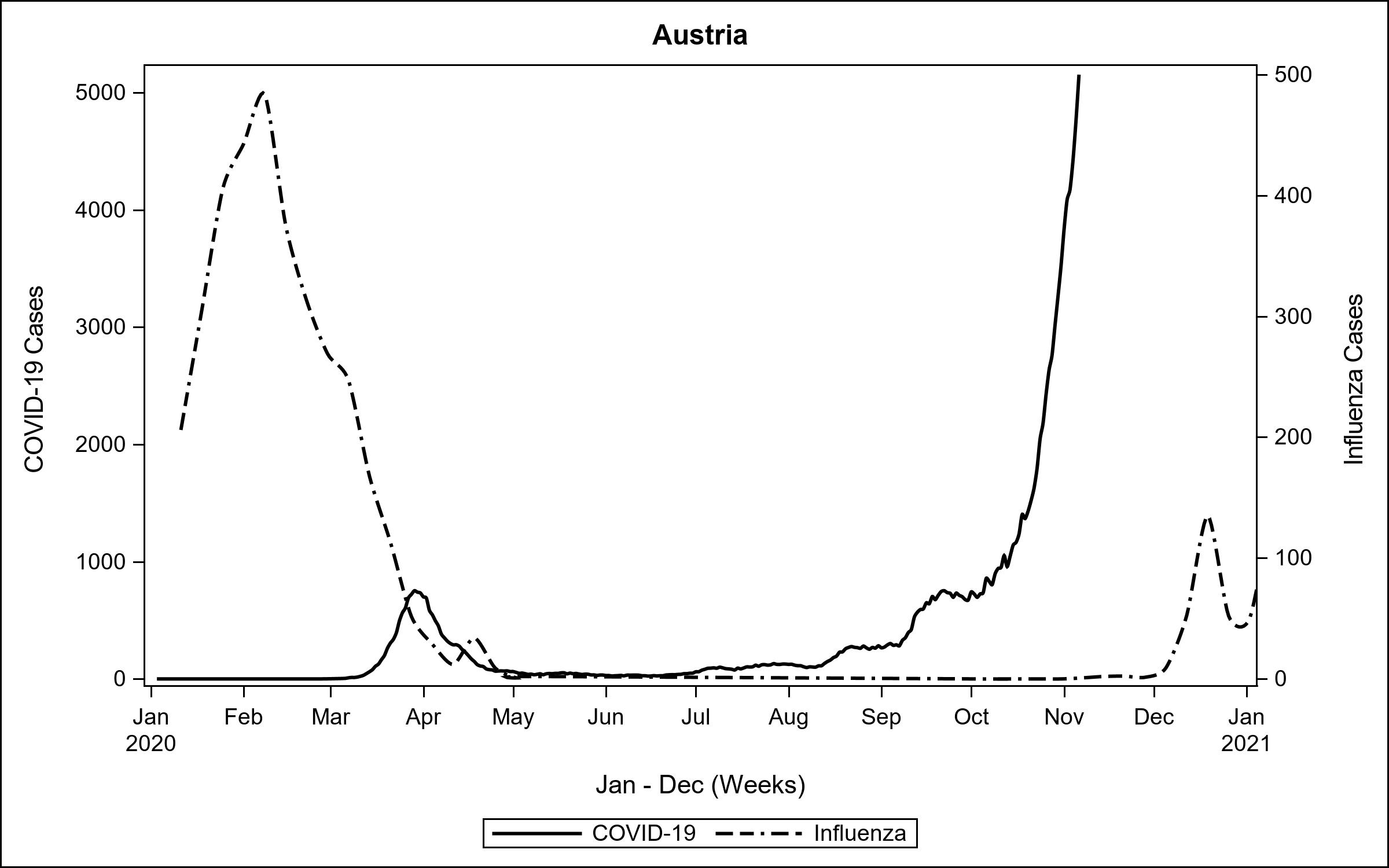

Supplement: Multimedia Appendix 4 [file publichealth_v7i3e24696_app4.zip › Country comparisons_all/Austria1.jpeg]

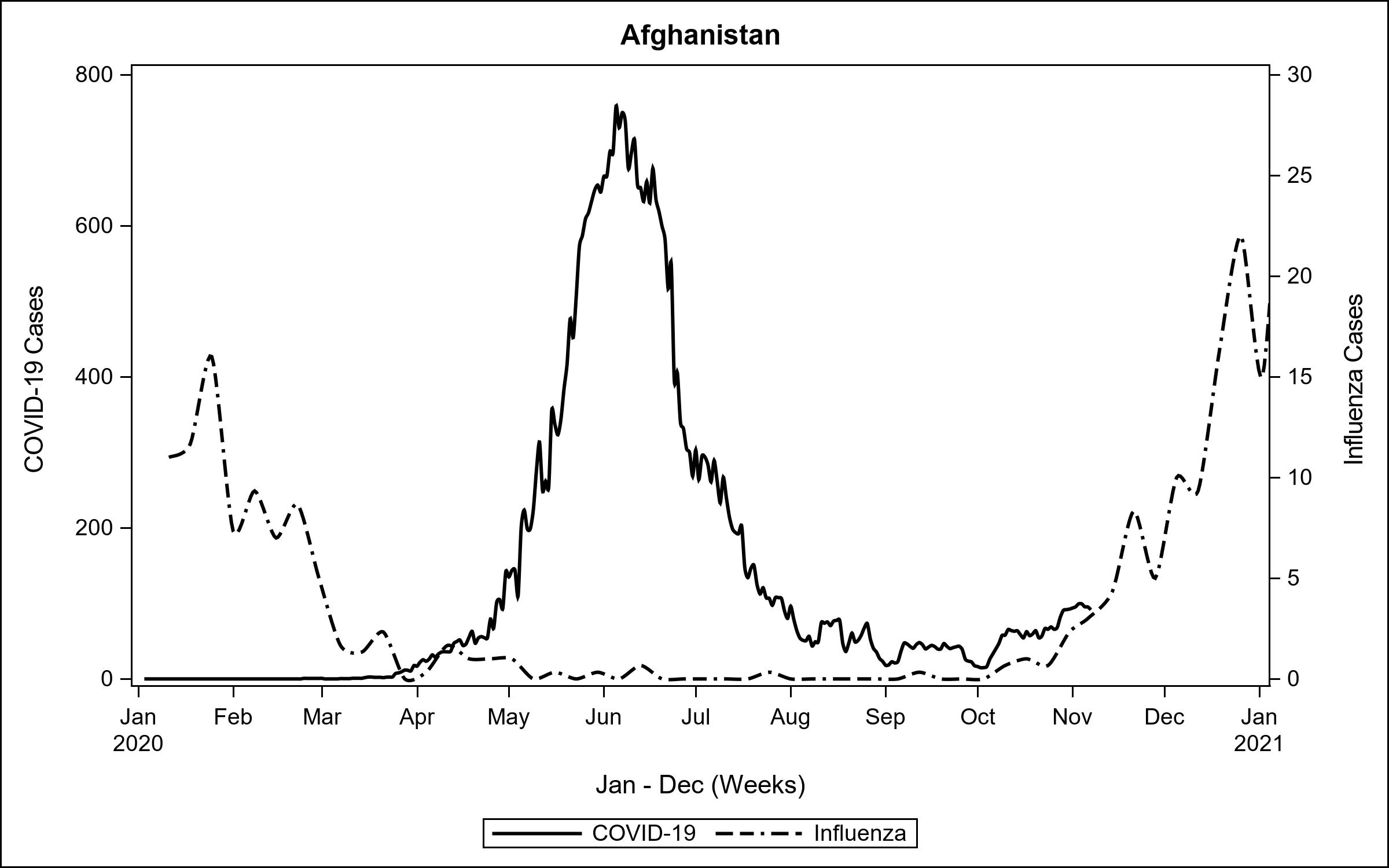

Supplement: Multimedia Appendix 4 [file publichealth_v7i3e24696_app4.zip › Country comparisons_all/Afghanistan1.jpeg]

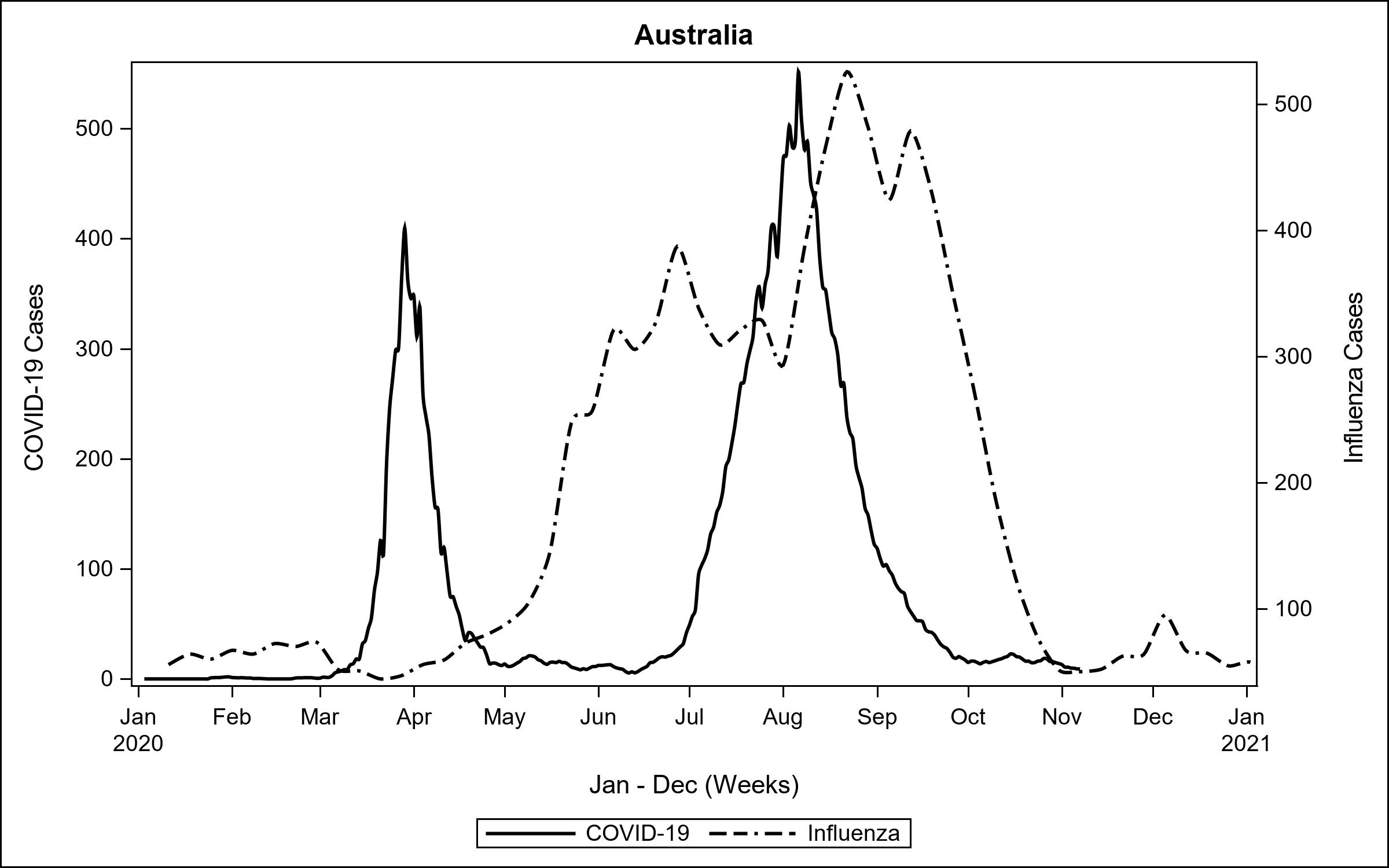

Supplement: Multimedia Appendix 4 [file publichealth_v7i3e24696_app4.zip › Country comparisons_all/Australia3.jpeg]

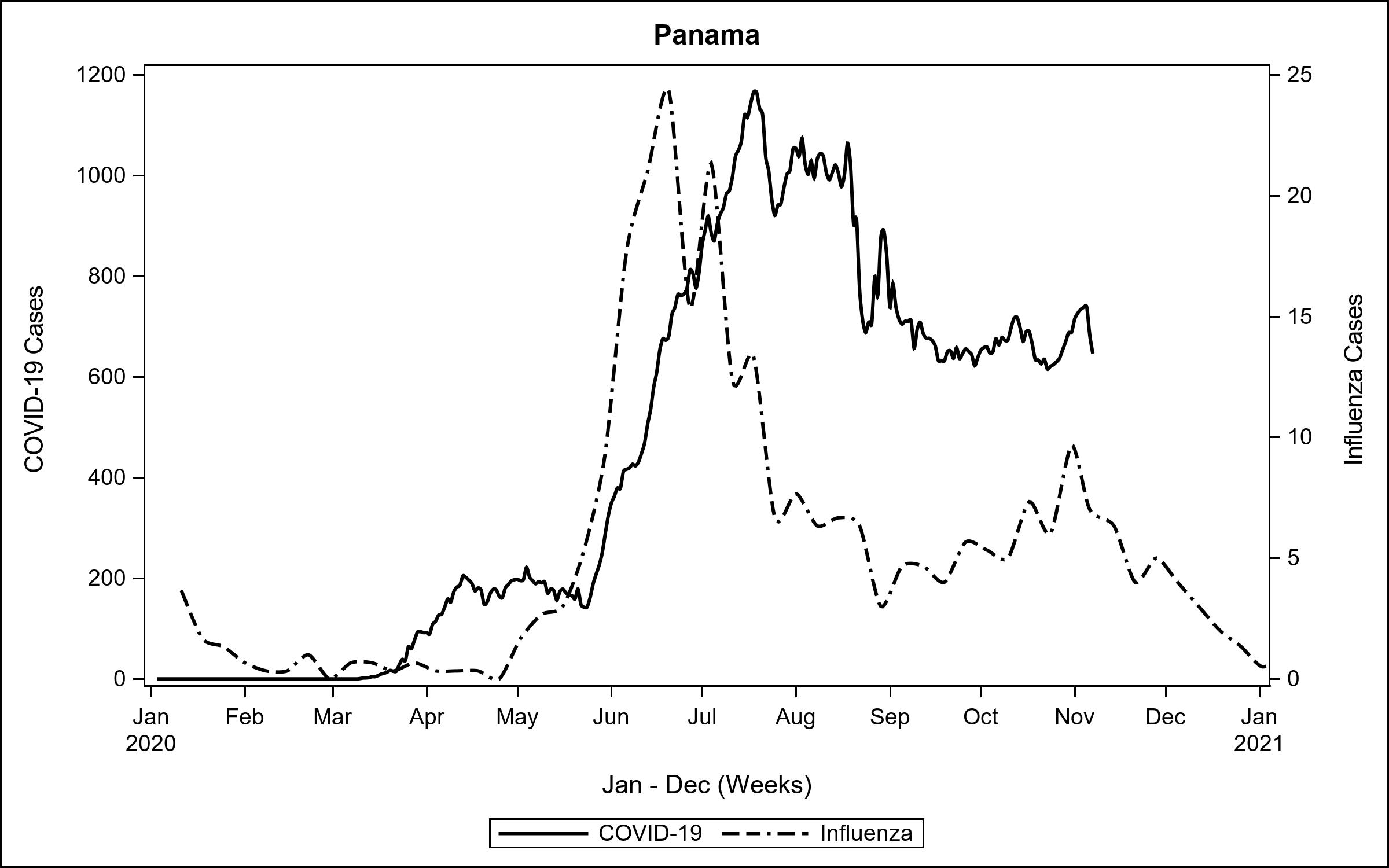

Supplement: Multimedia Appendix 4 [file publichealth_v7i3e24696_app4.zip › Country comparisons_all/Panama1.jpeg]

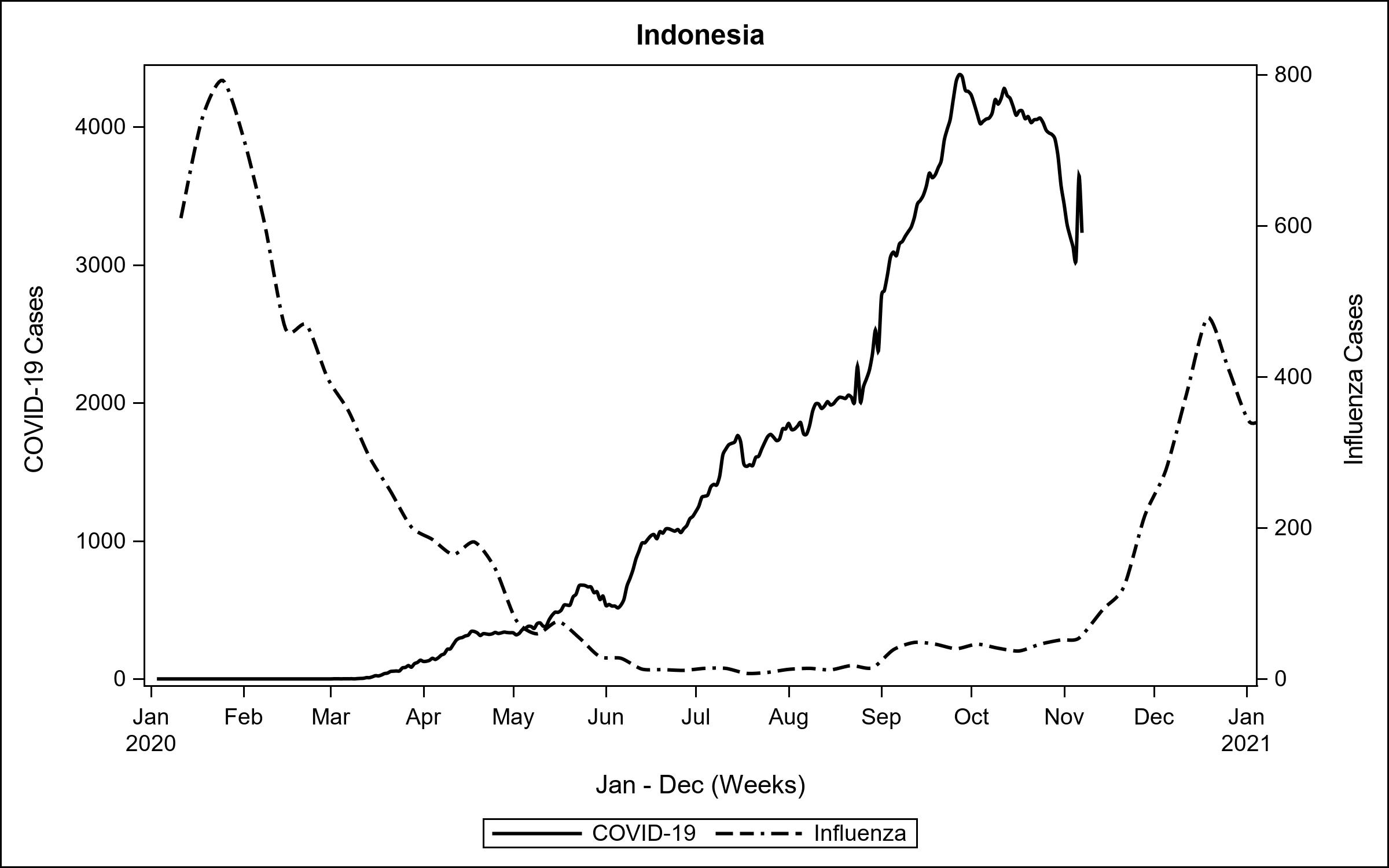

Supplement: Multimedia Appendix 4 [file publichealth_v7i3e24696_app4.zip › Country comparisons_all/Indonesia1.jpeg]

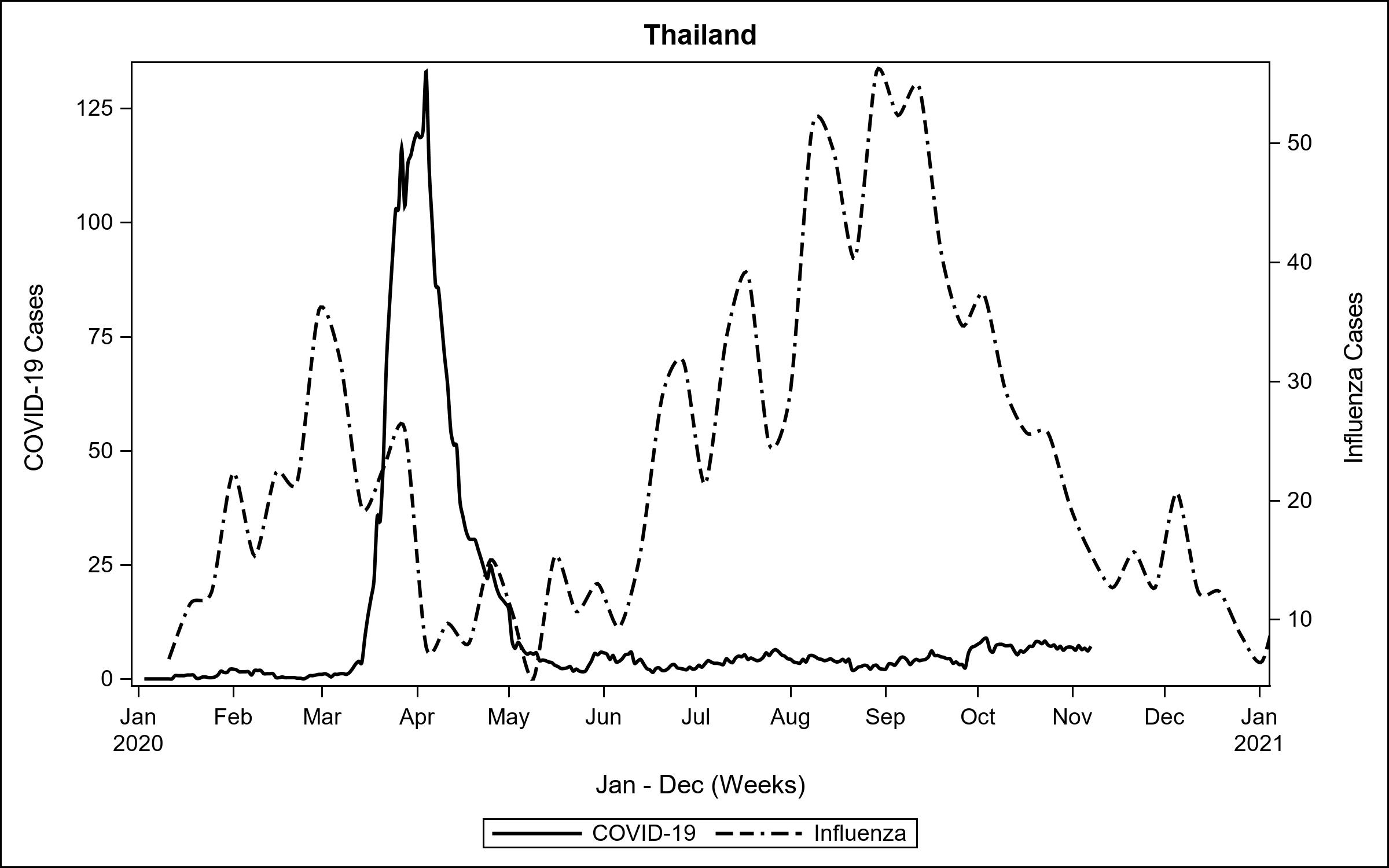

Supplement: Multimedia Appendix 4 [file publichealth_v7i3e24696_app4.zip › Country comparisons_all/Thailand1.jpeg]

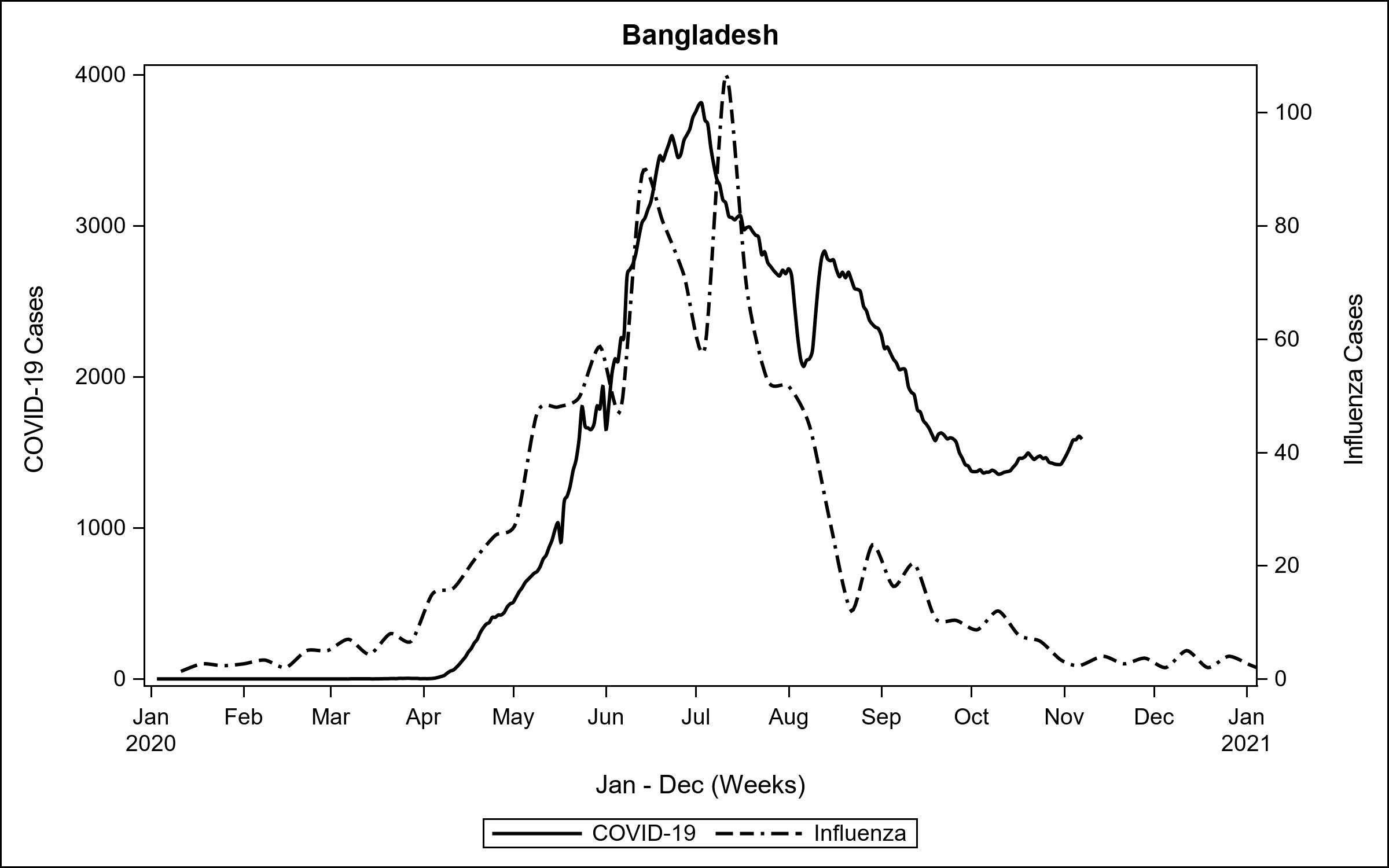

Supplement: Multimedia Appendix 4 [file publichealth_v7i3e24696_app4.zip › Country comparisons_all/Bangladesh1.jpeg]

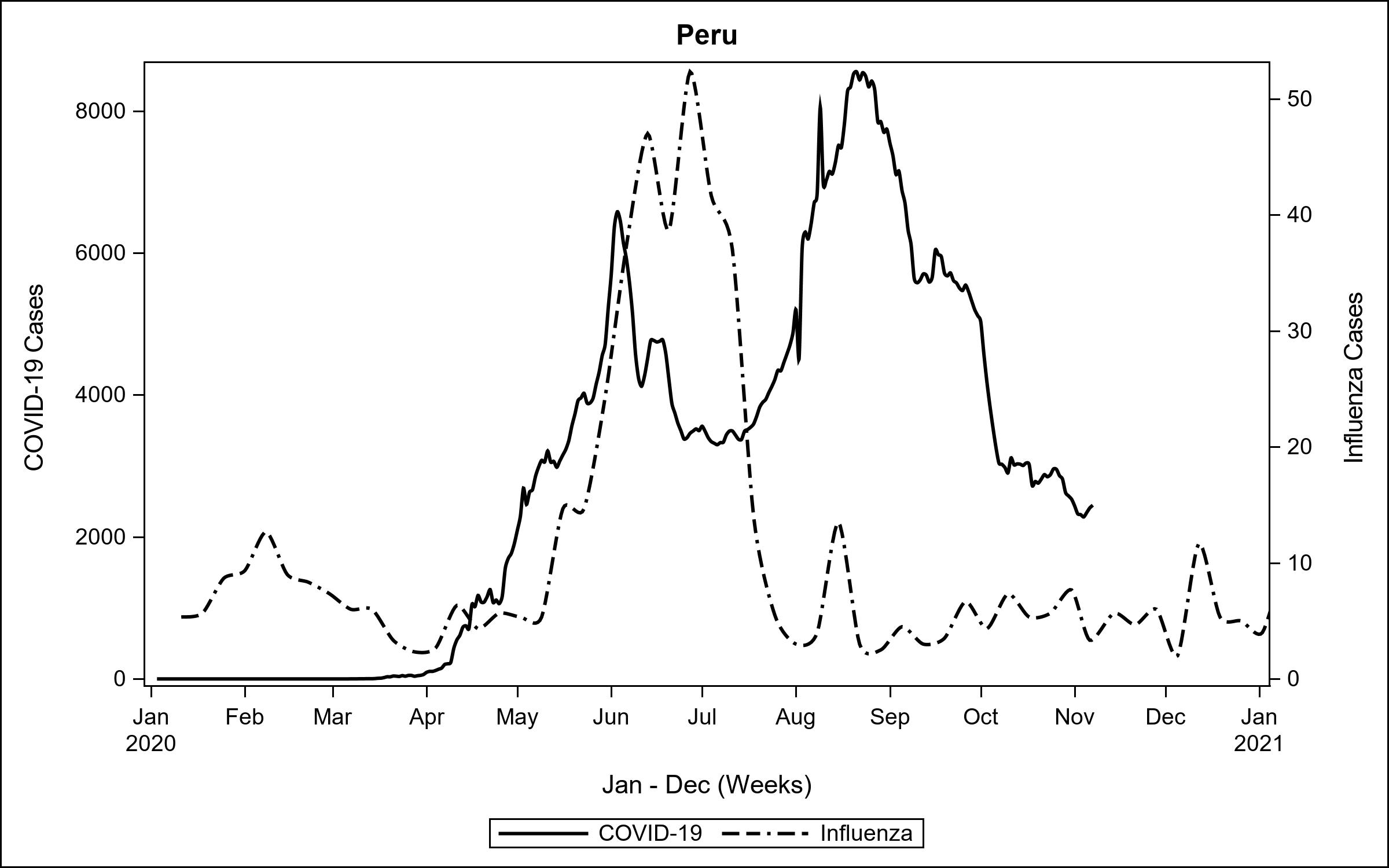

Supplement: Multimedia Appendix 4 [file publichealth_v7i3e24696_app4.zip › Country comparisons_all/Peru1.jpeg]

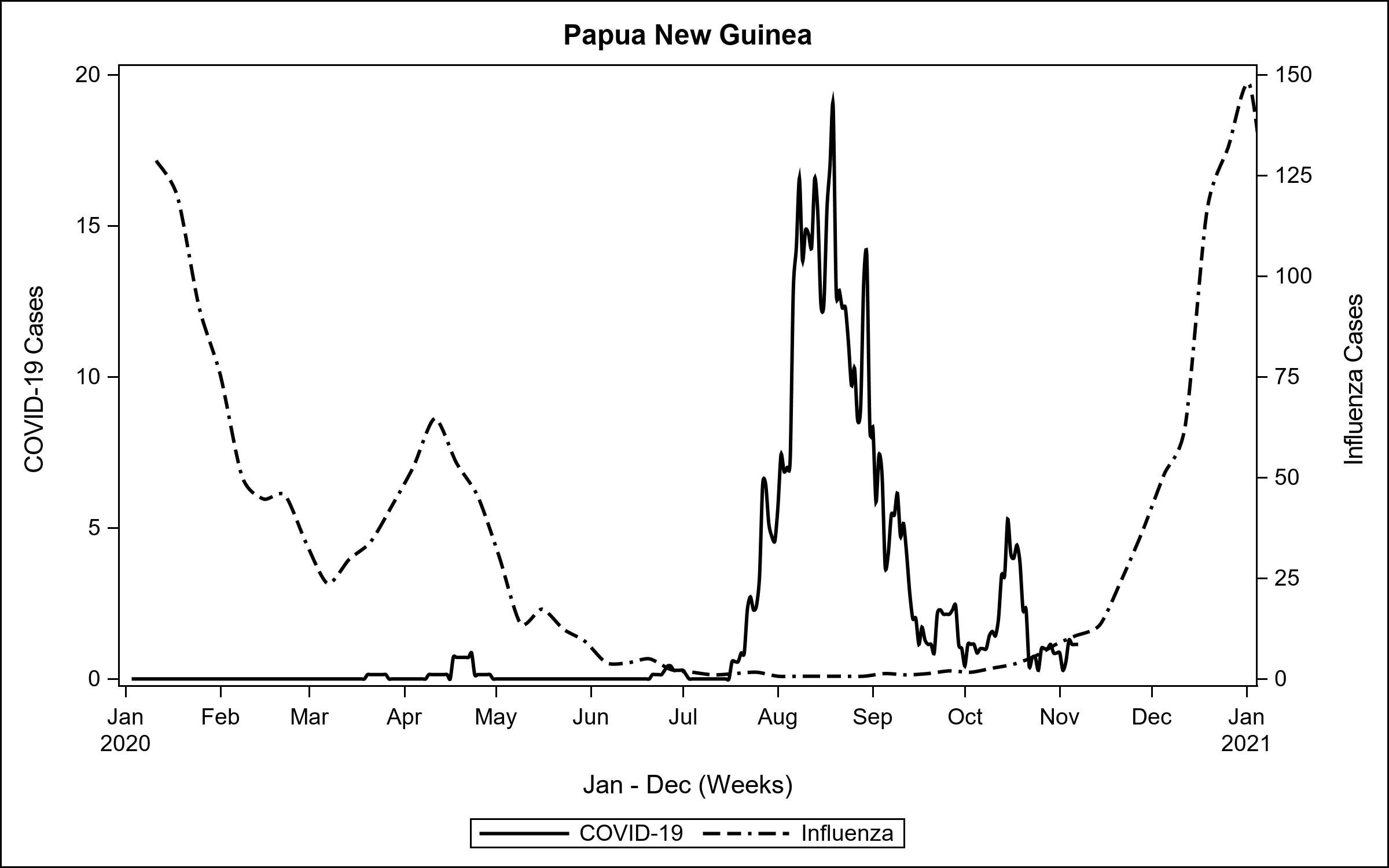

Supplement: Multimedia Appendix 4 [file publichealth_v7i3e24696_app4.zip › Country comparisons_all/Papua New Guinea1.jpeg]

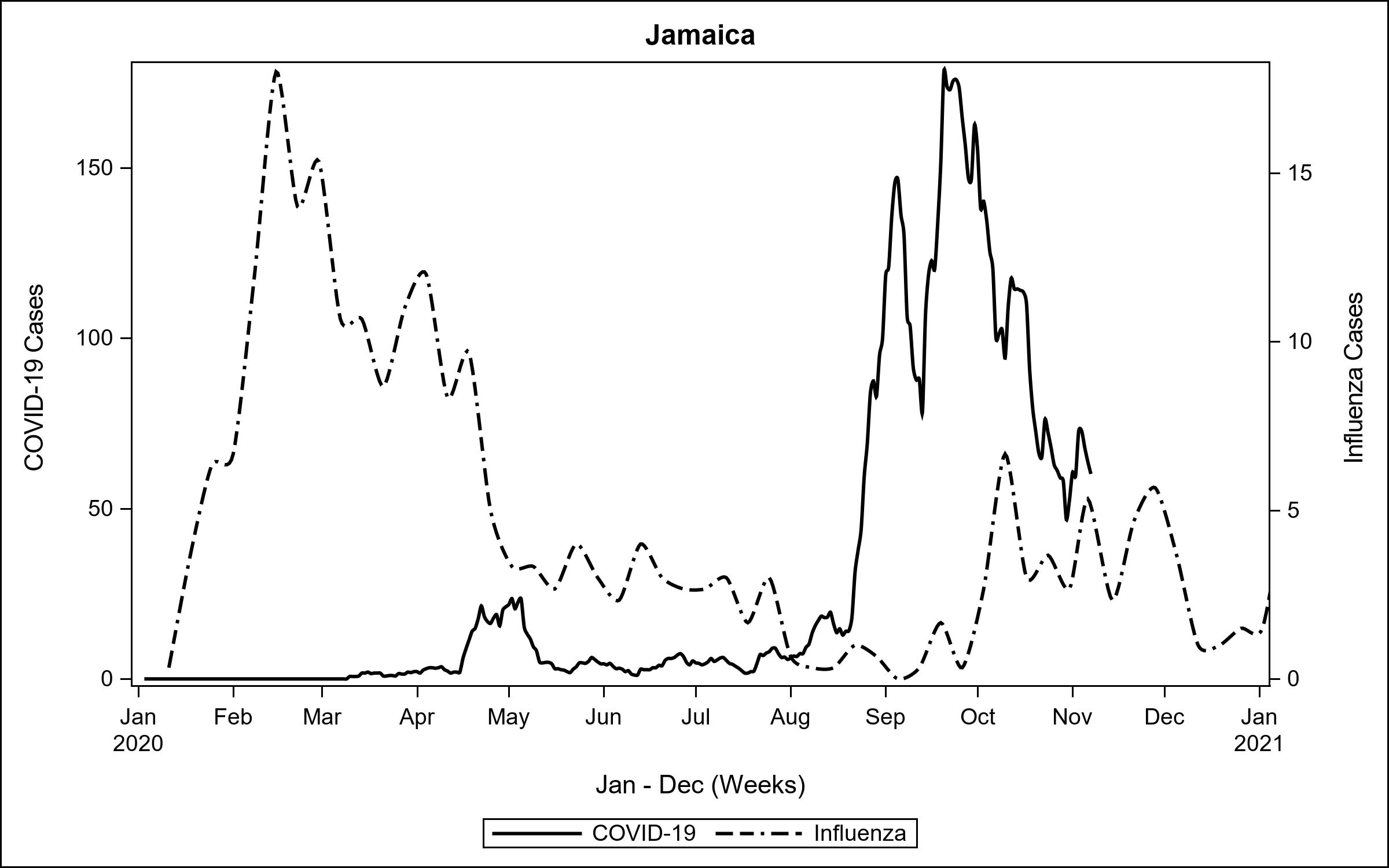

Supplement: Multimedia Appendix 4 [file publichealth_v7i3e24696_app4.zip › Country comparisons_all/Jamaica1.jpeg]

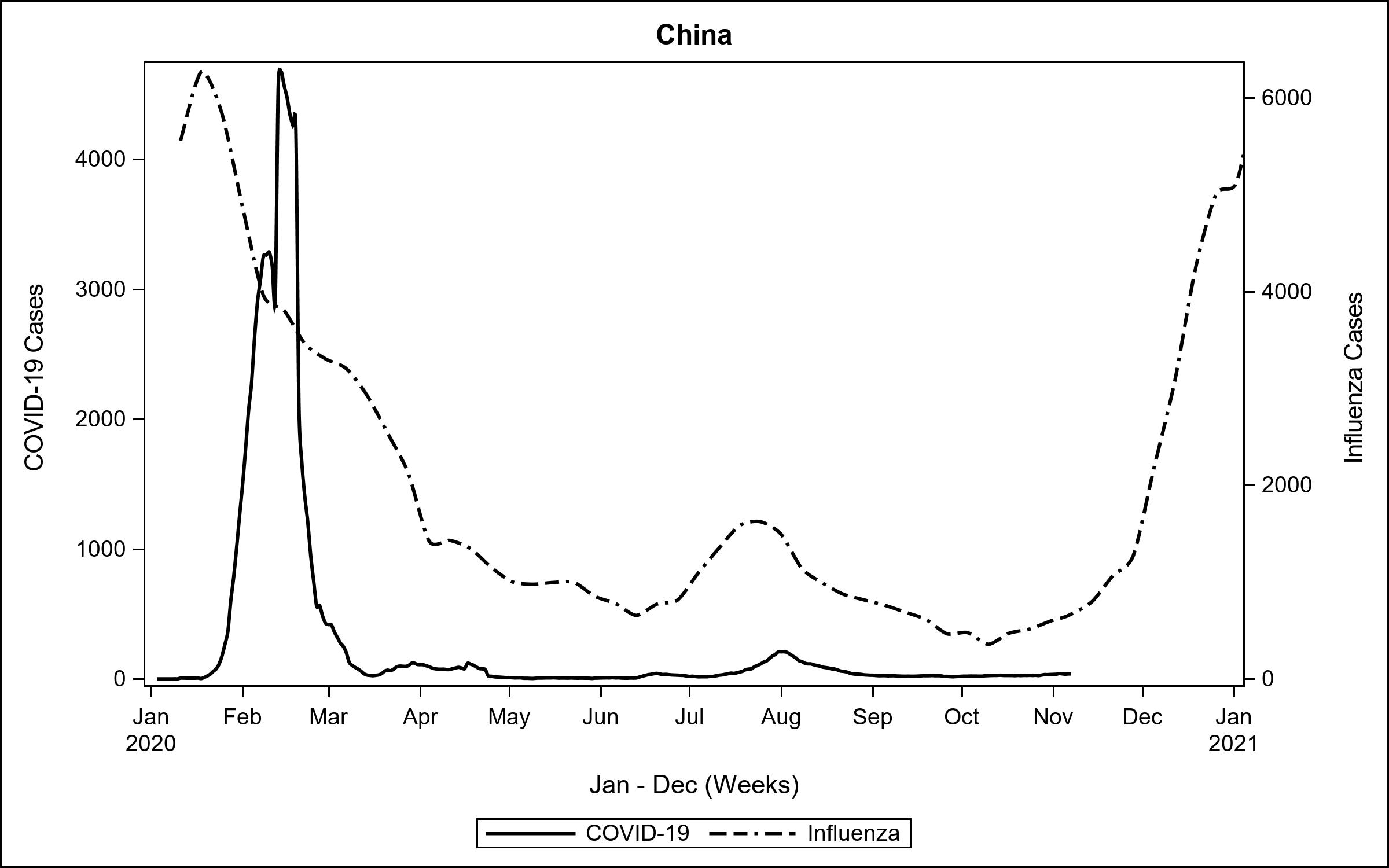

Supplement: Multimedia Appendix 4 [file publichealth_v7i3e24696_app4.zip › Country comparisons_all/China1.jpeg]

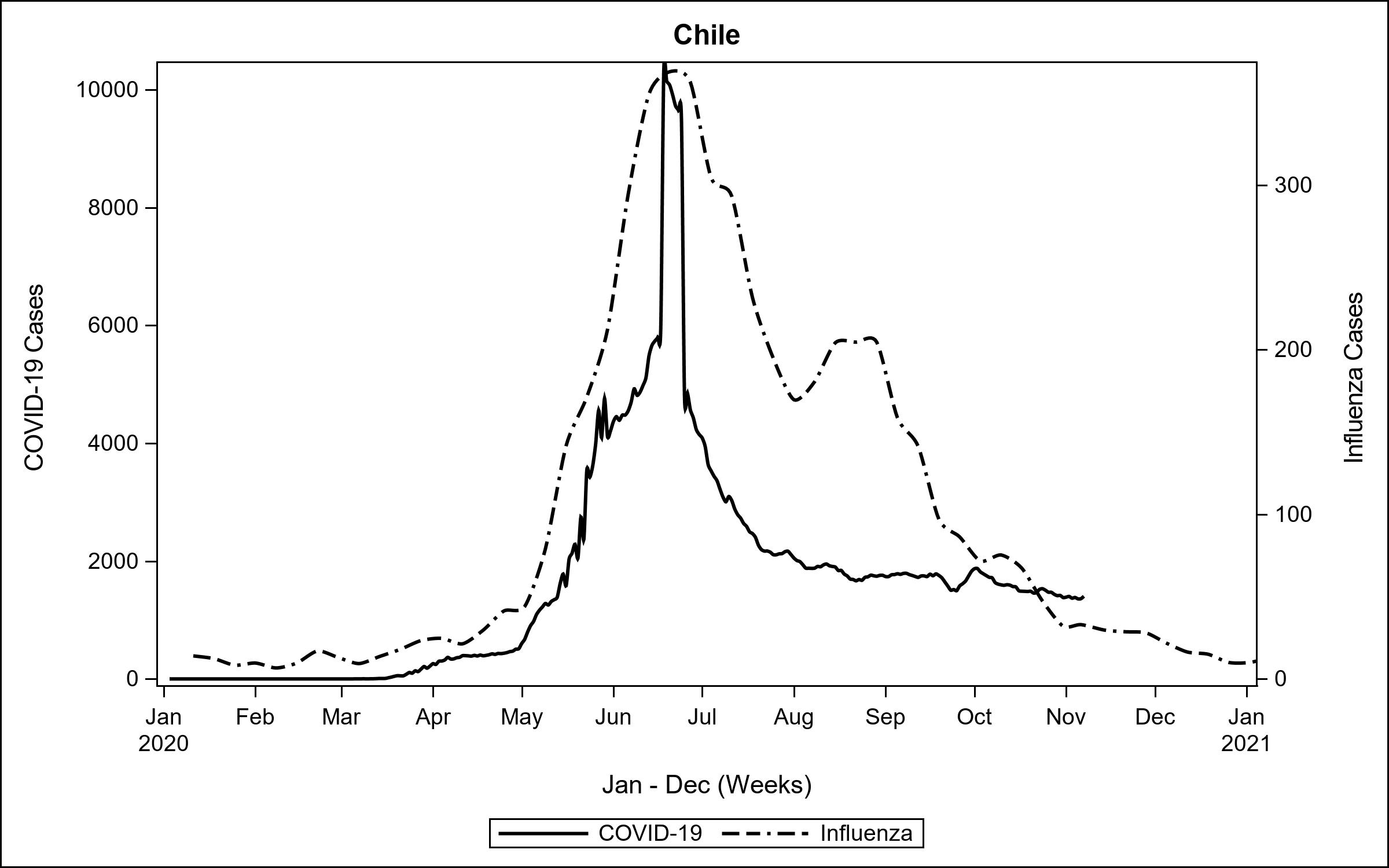

Supplement: Multimedia Appendix 4 [file publichealth_v7i3e24696_app4.zip › Country comparisons_all/Chile1.jpeg]

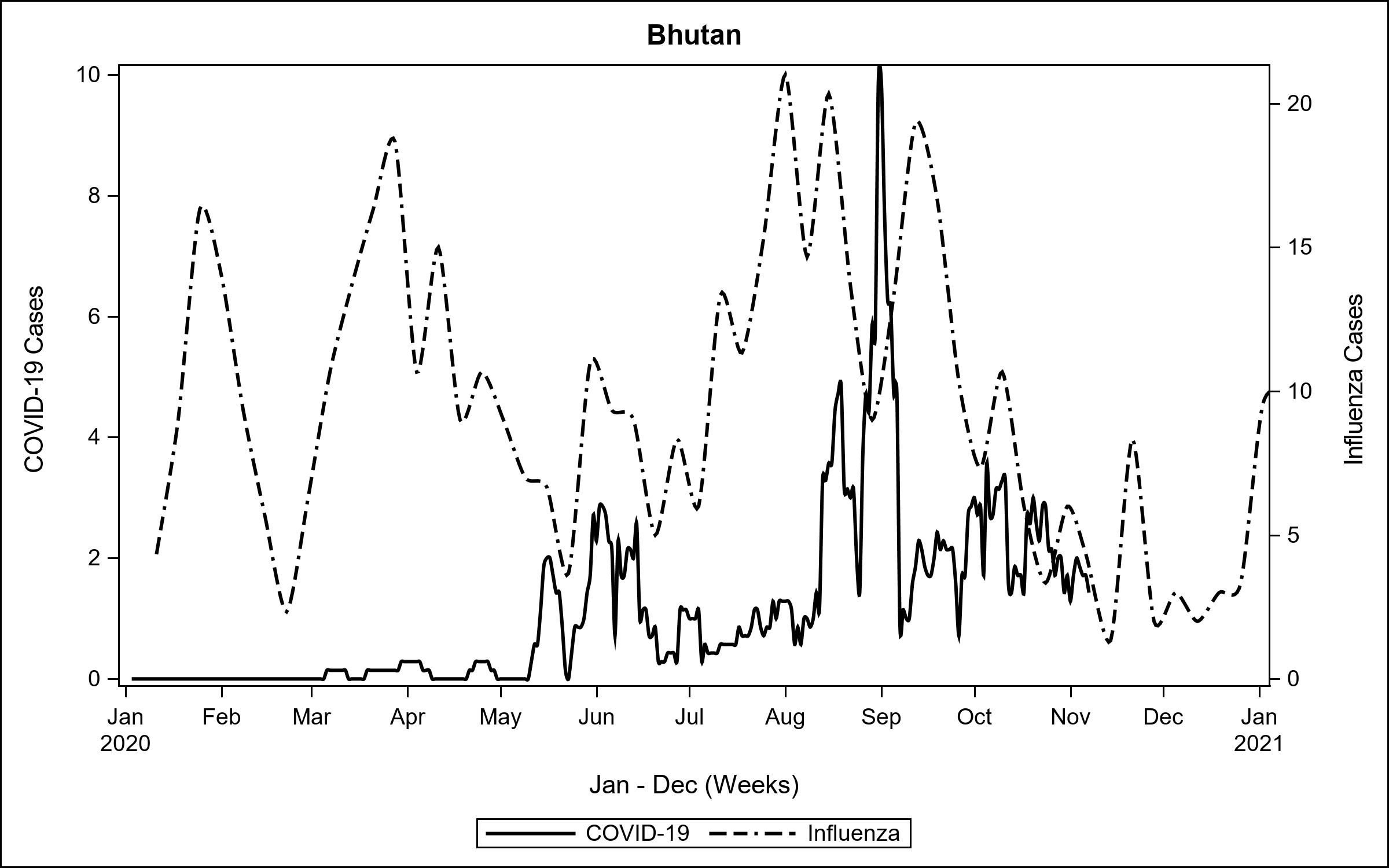

Supplement: Multimedia Appendix 4 [file publichealth_v7i3e24696_app4.zip › Country comparisons_all/Bhutan1.jpeg]

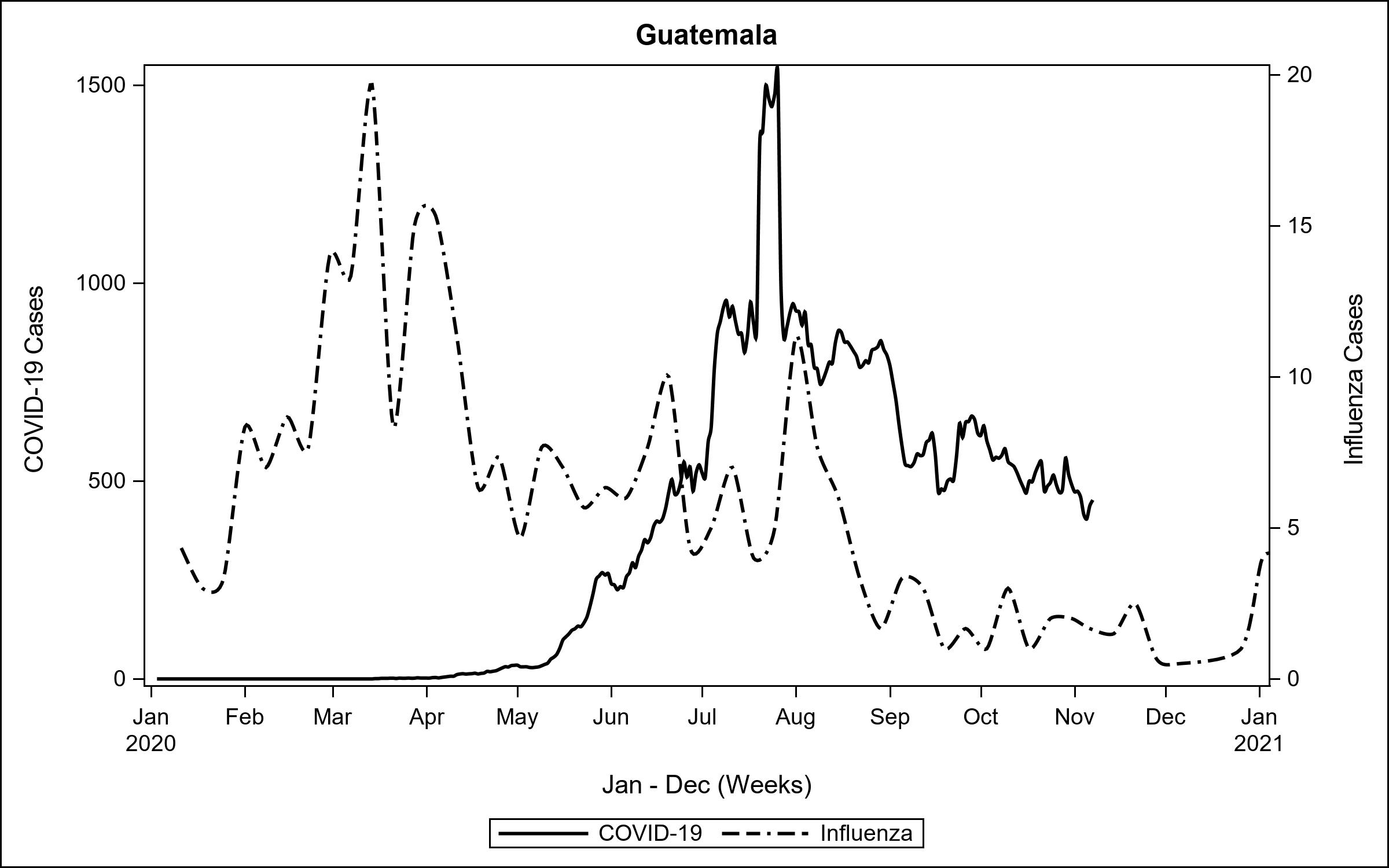

Supplement: Multimedia Appendix 4 [file publichealth_v7i3e24696_app4.zip › Country comparisons_all/Guatemala1.jpeg]

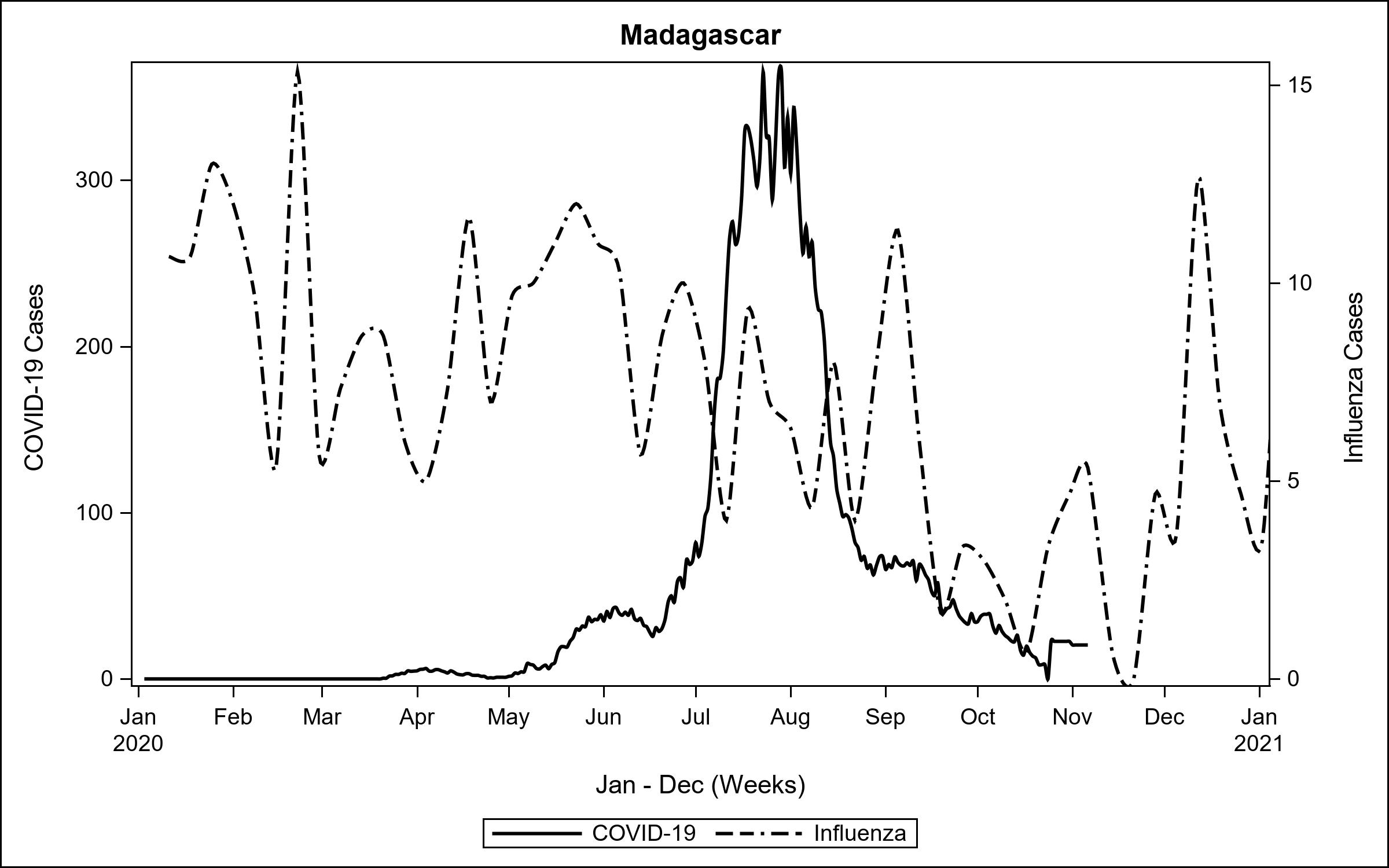

Supplement: Multimedia Appendix 4 [file publichealth_v7i3e24696_app4.zip › Country comparisons_all/Madagascar1.jpeg]

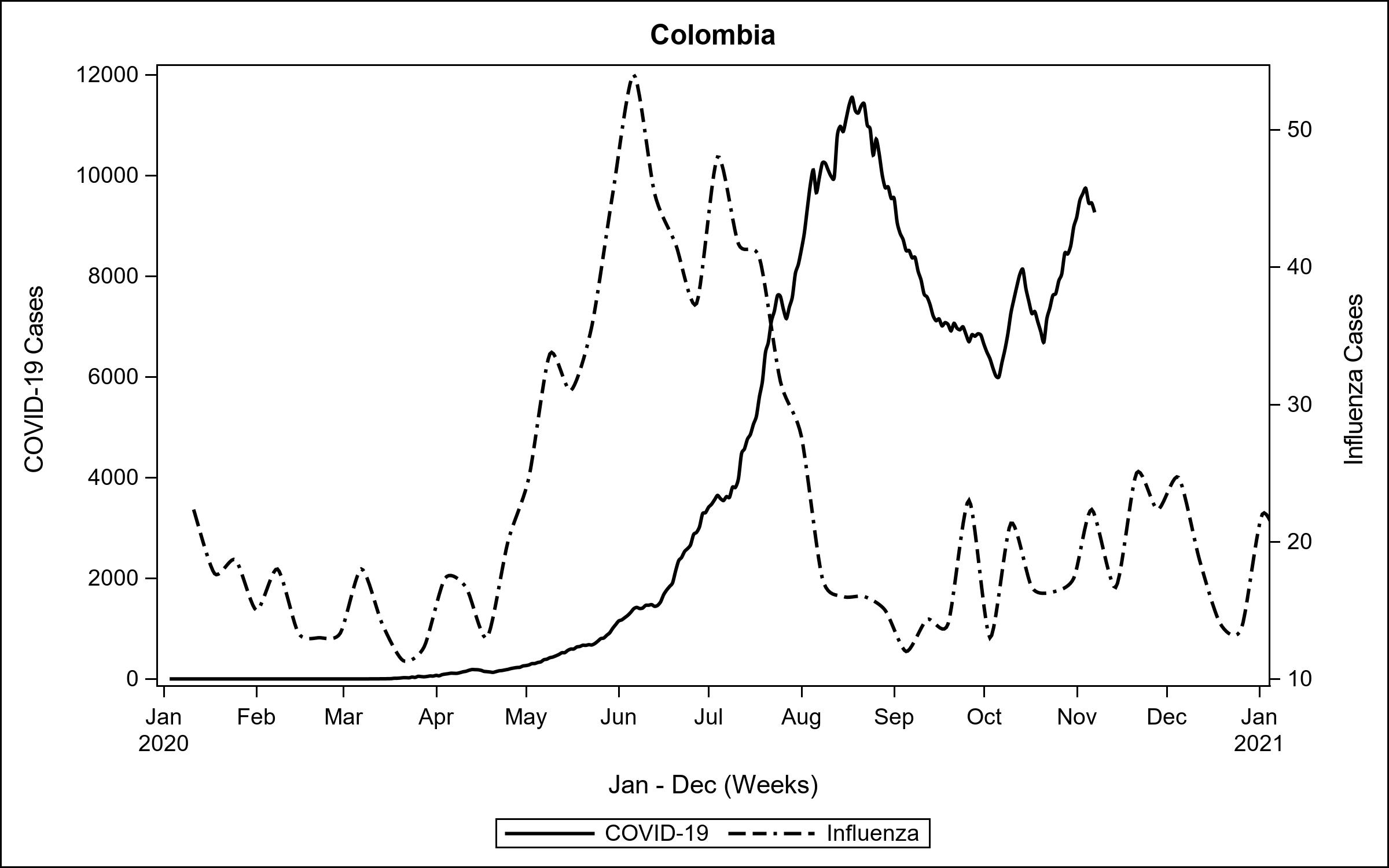

Supplement: Multimedia Appendix 4 [file publichealth_v7i3e24696_app4.zip › Country comparisons_all/Colombia1.jpeg]

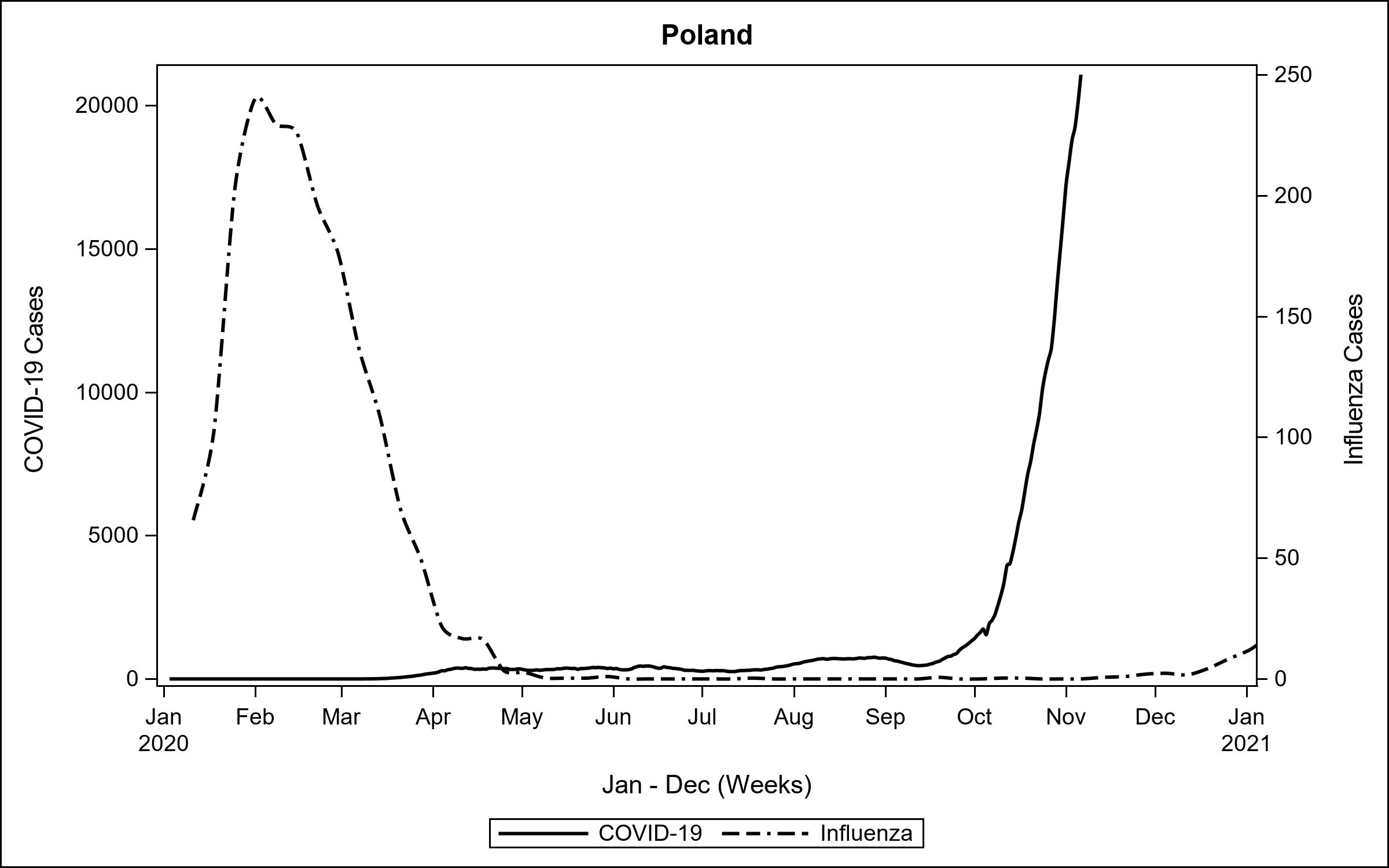

Supplement: Multimedia Appendix 4 [file publichealth_v7i3e24696_app4.zip › Country comparisons_all/Poland1.jpeg]

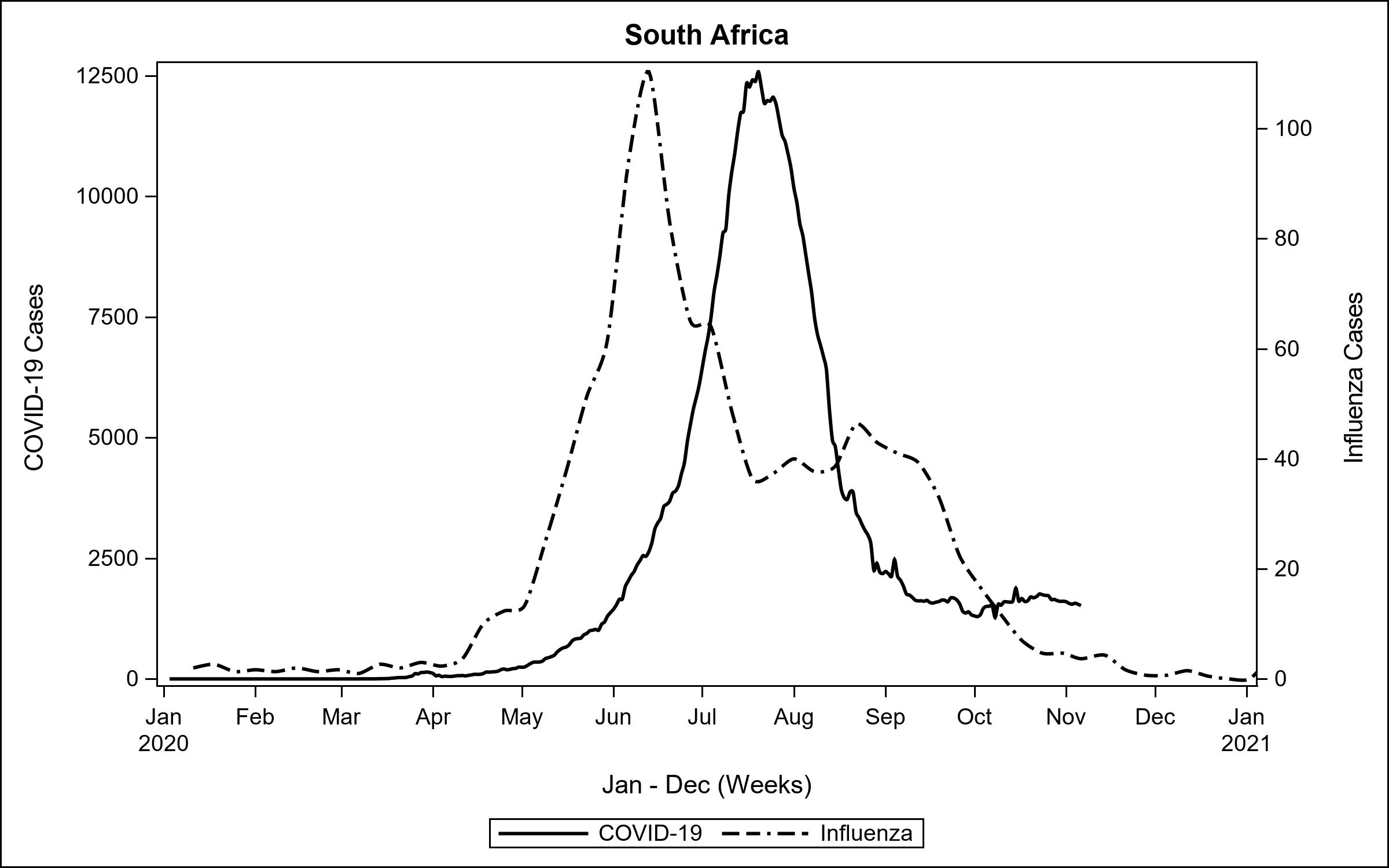

Supplement: Multimedia Appendix 4 [file publichealth_v7i3e24696_app4.zip › Country comparisons_all/South Africa3.jpeg]

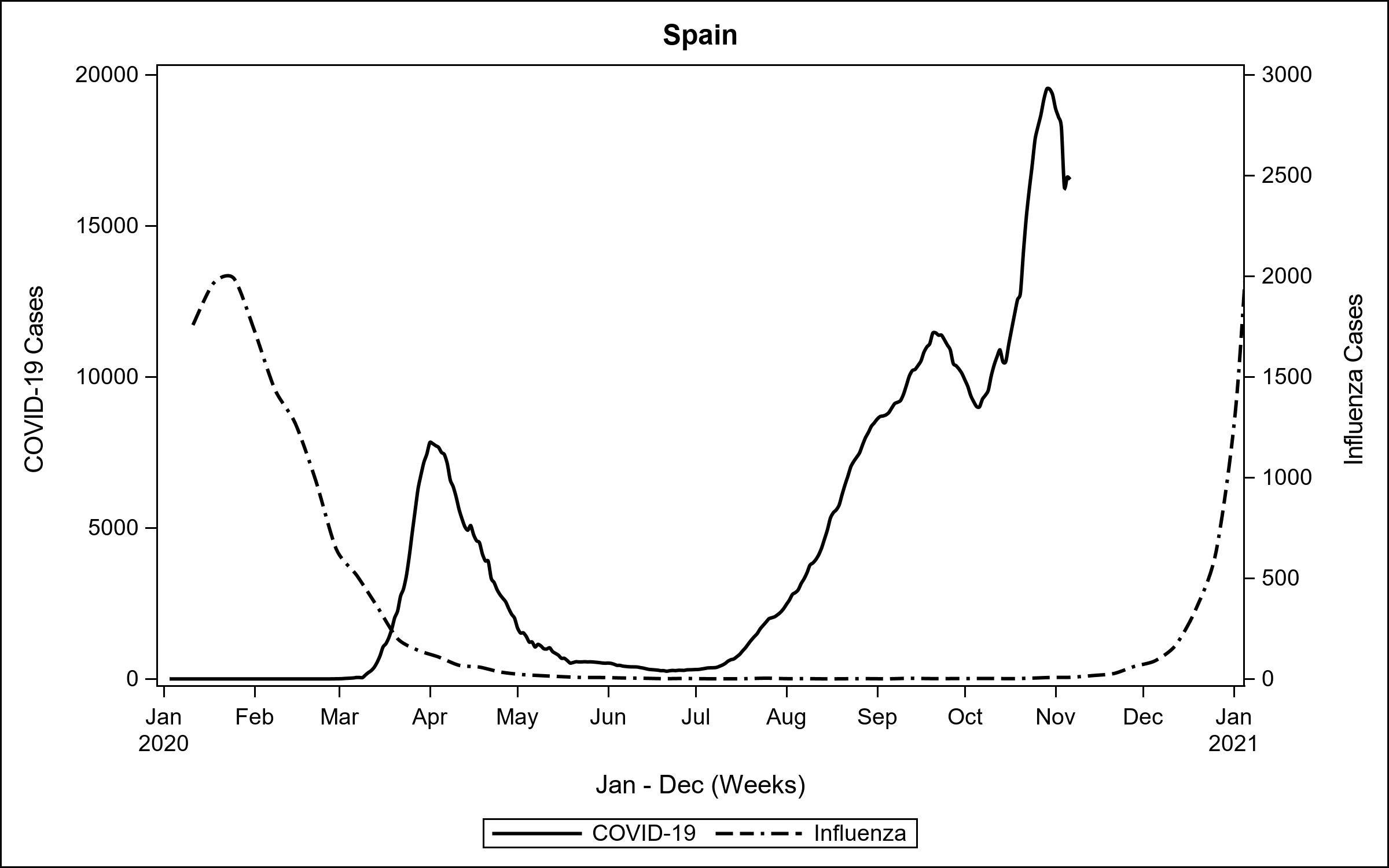

Supplement: Multimedia Appendix 4 [file publichealth_v7i3e24696_app4.zip › Country comparisons_all/Spain1.jpeg]

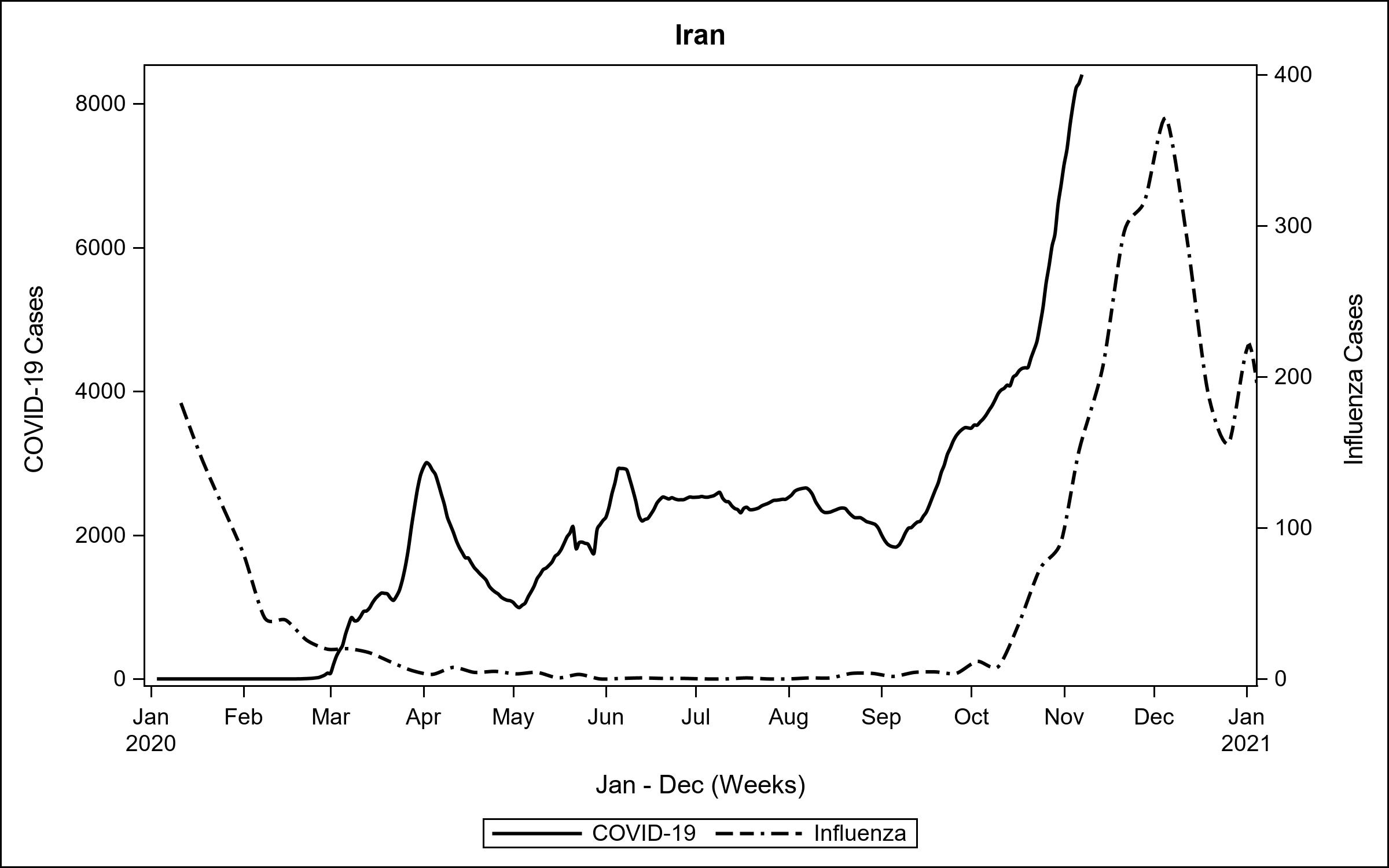

Supplement: Multimedia Appendix 4 [file publichealth_v7i3e24696_app4.zip › Country comparisons_all/Iran1.jpeg]

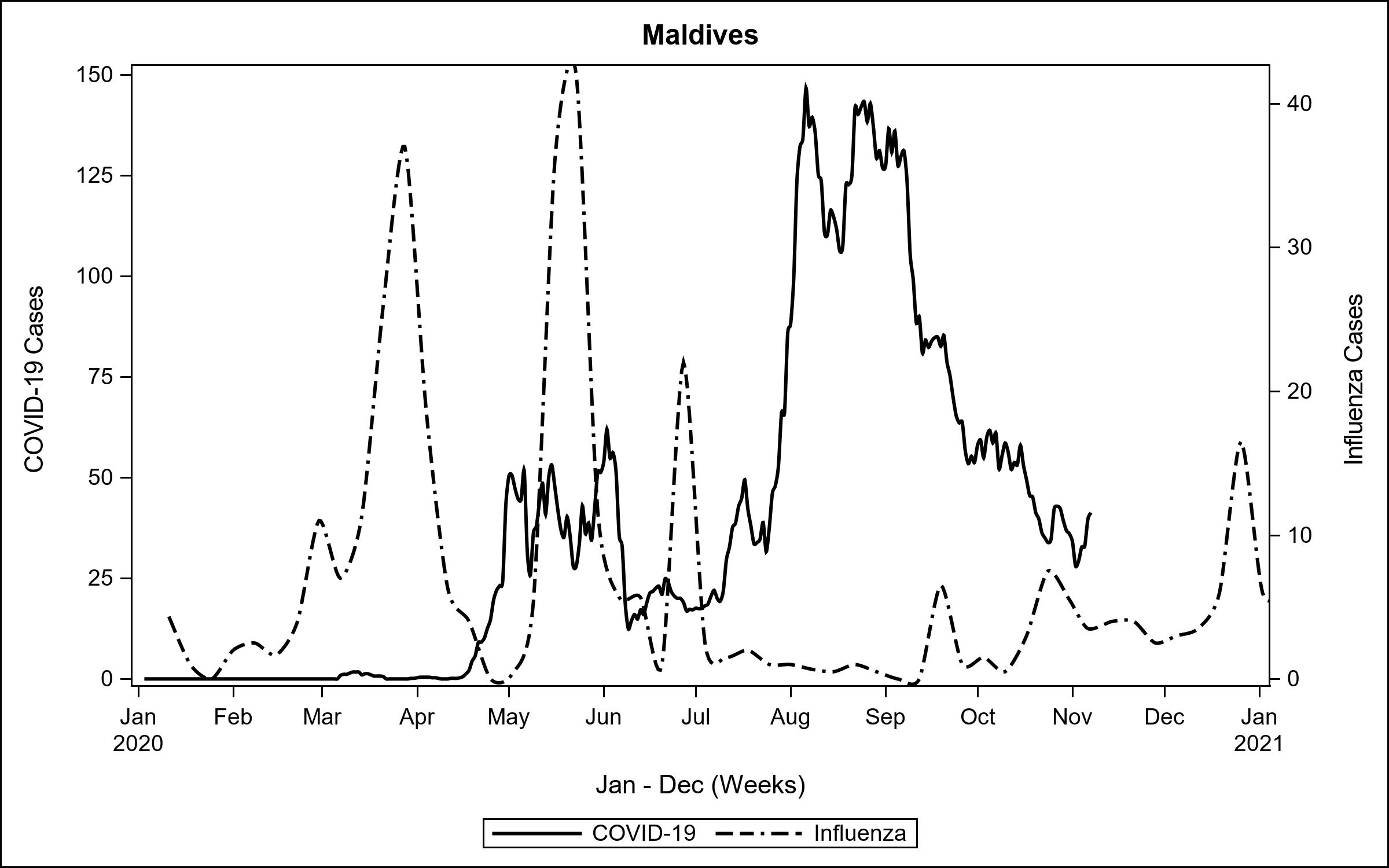

Supplement: Multimedia Appendix 4 [file publichealth_v7i3e24696_app4.zip › Country comparisons_all/Maldives1.jpeg]

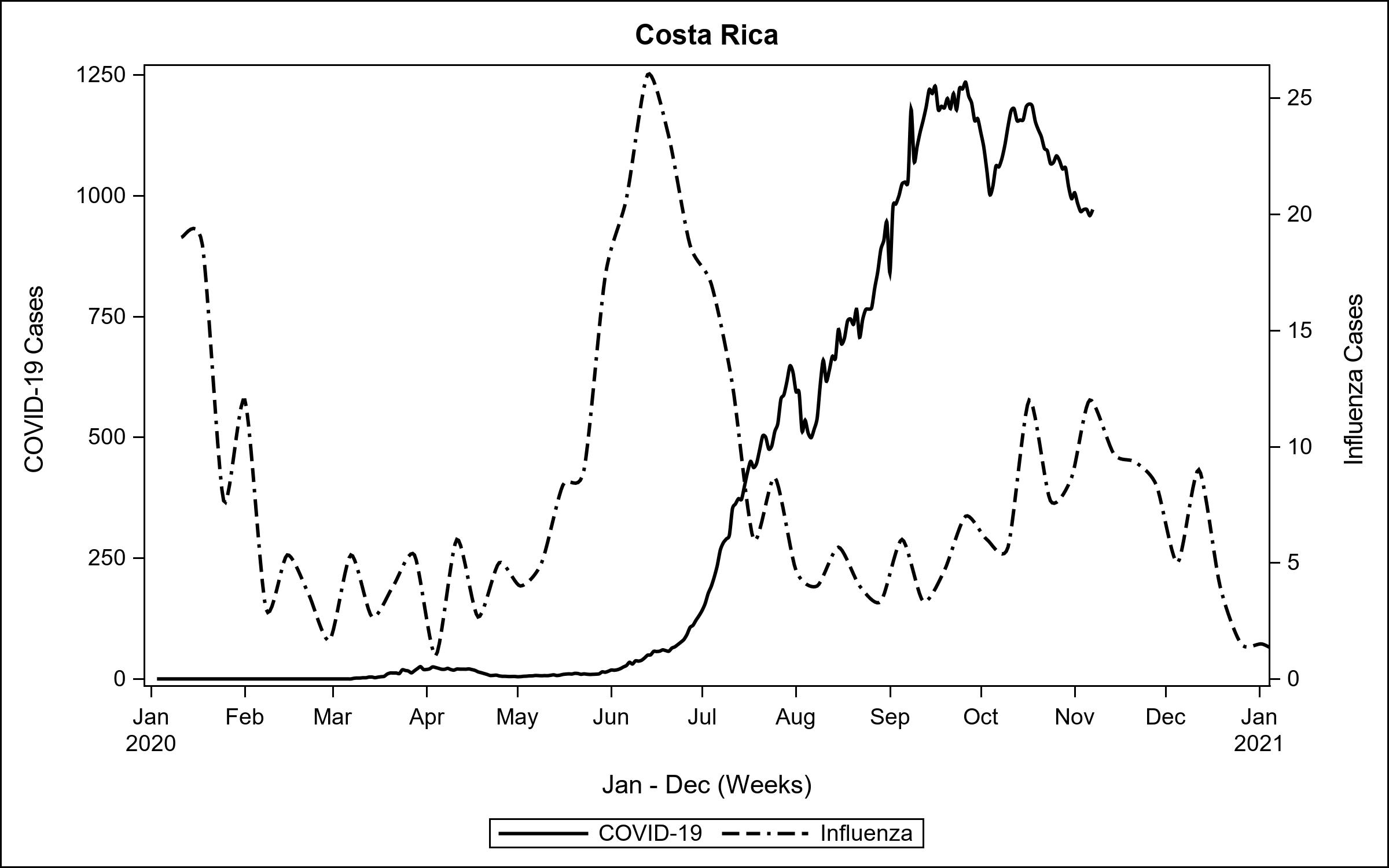

Supplement: Multimedia Appendix 4 [file publichealth_v7i3e24696_app4.zip › Country comparisons_all/Costa Rica1.jpeg]

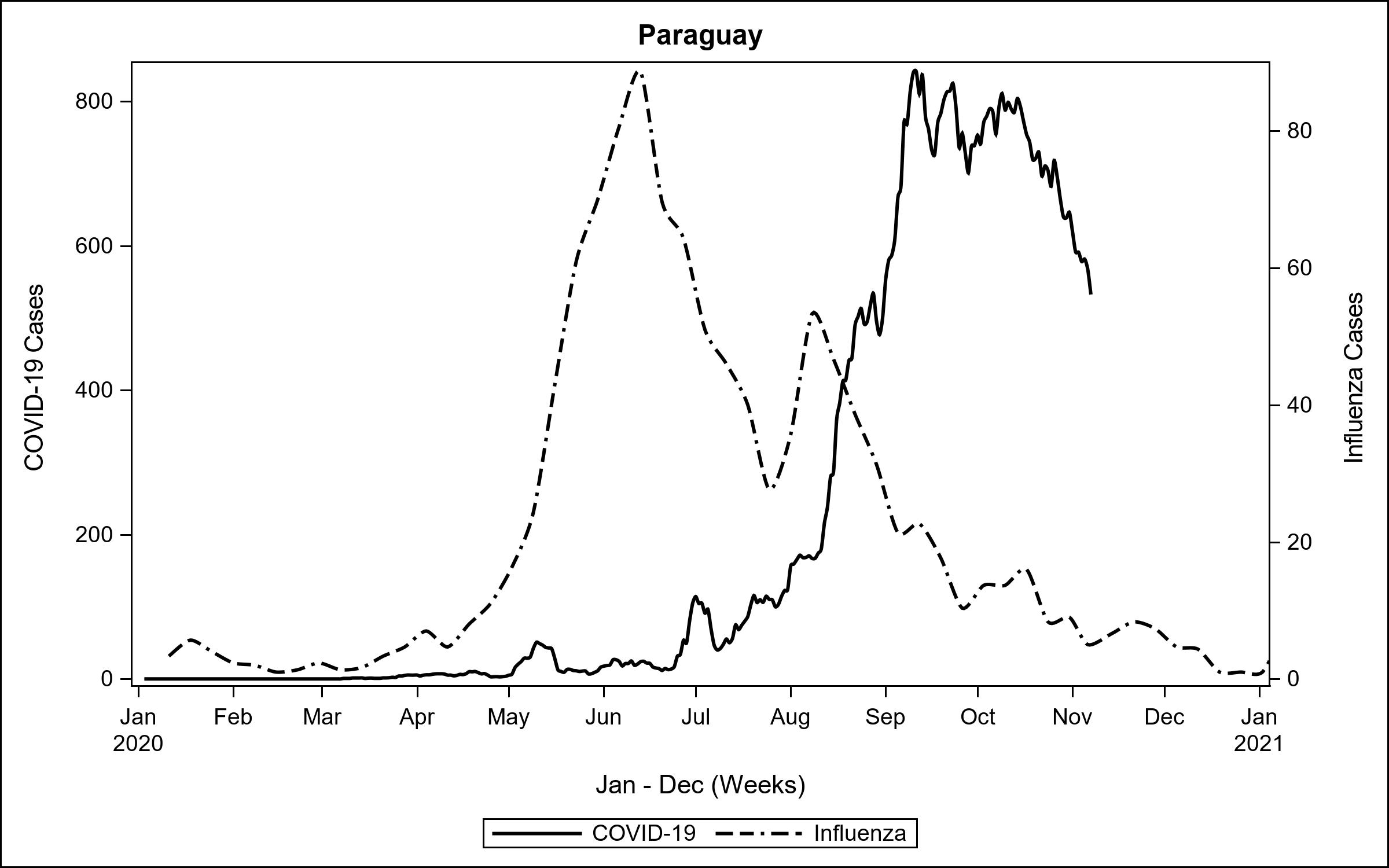

Supplement: Multimedia Appendix 4 [file publichealth_v7i3e24696_app4.zip › Country comparisons_all/Paraguay1.jpeg]

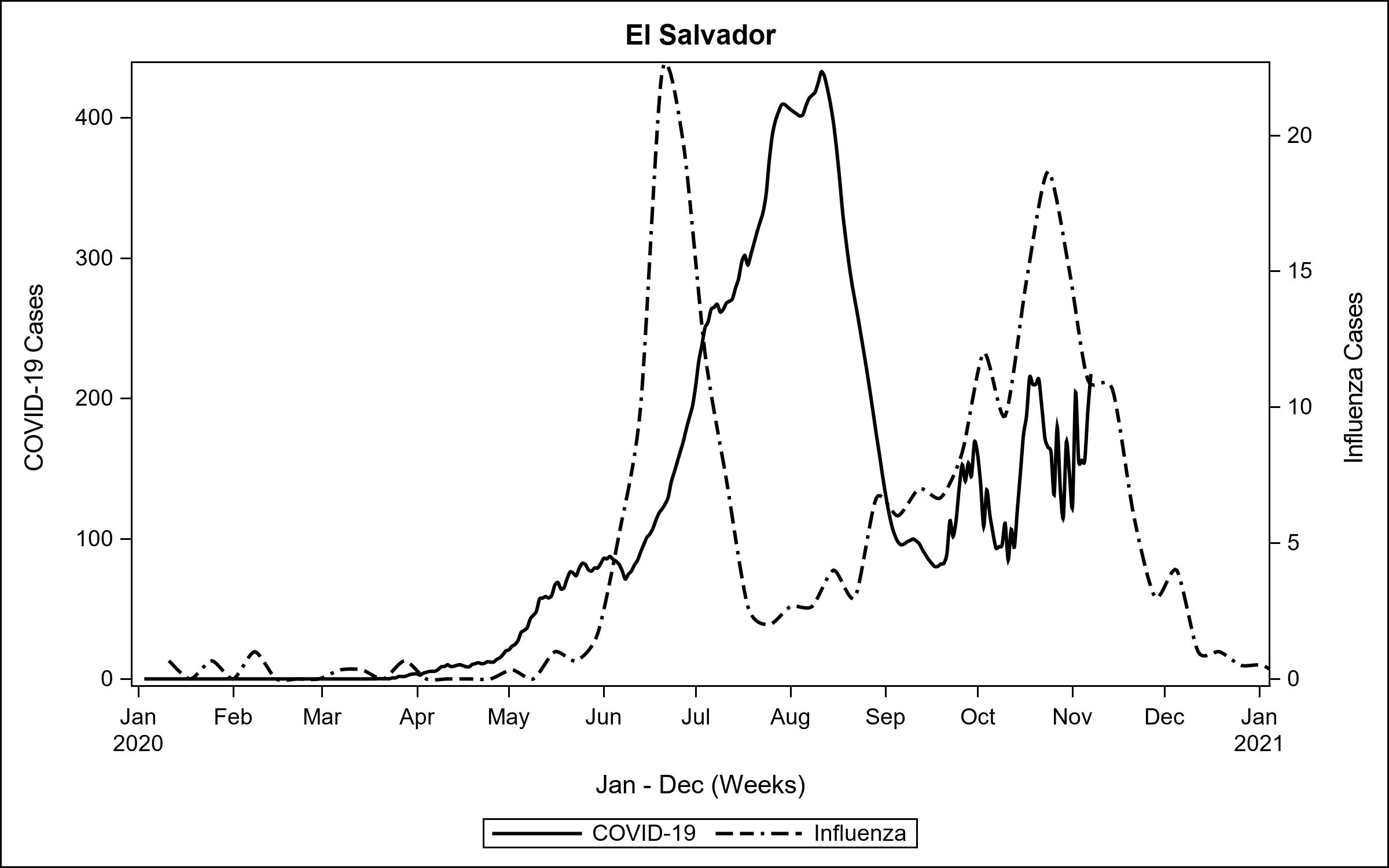

Supplement: Multimedia Appendix 4 [file publichealth_v7i3e24696_app4.zip › Country comparisons_all/El Salvador1.jpeg]

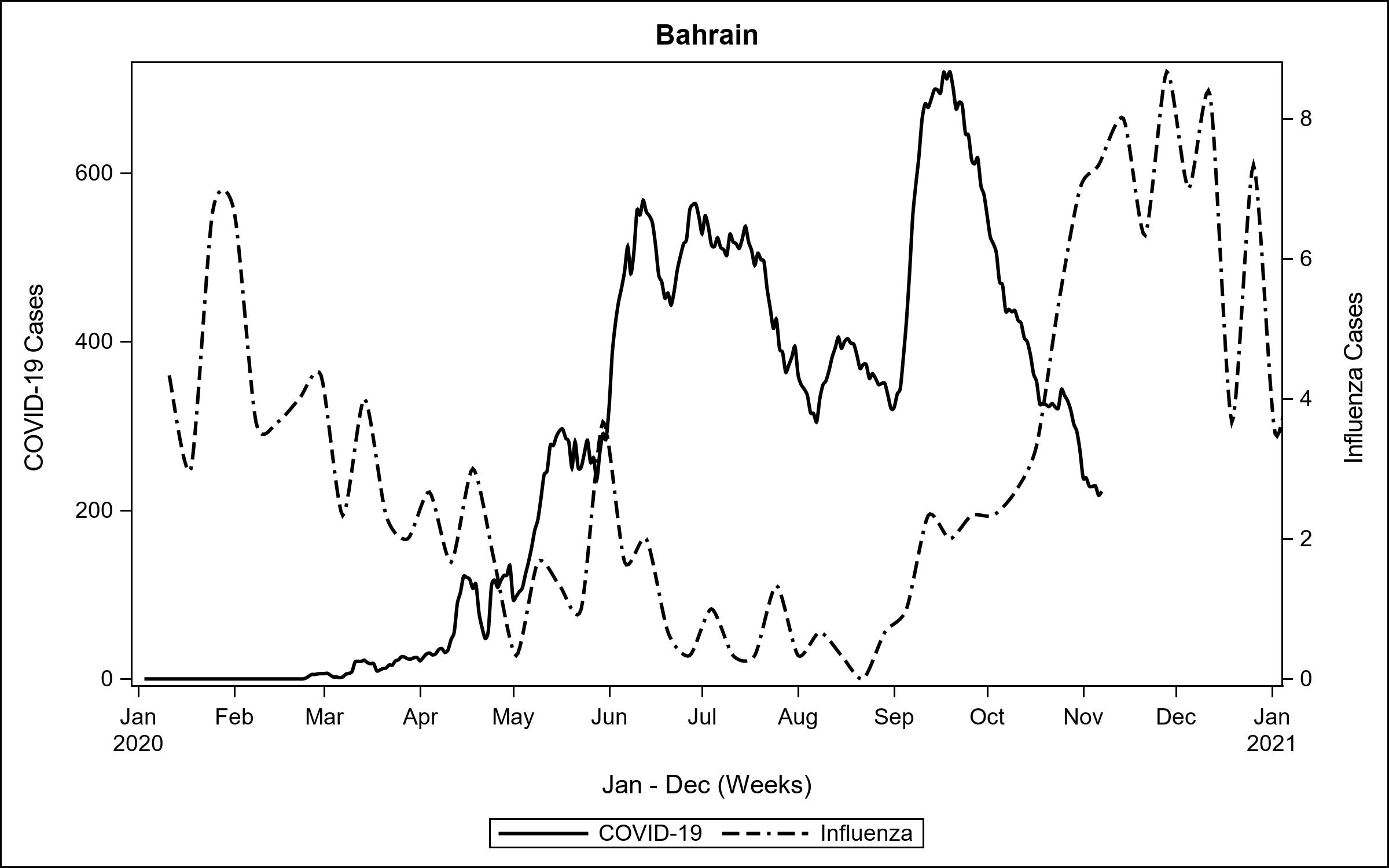

Supplement: Multimedia Appendix 4 [file publichealth_v7i3e24696_app4.zip › Country comparisons_all/Bahrain1.jpeg]

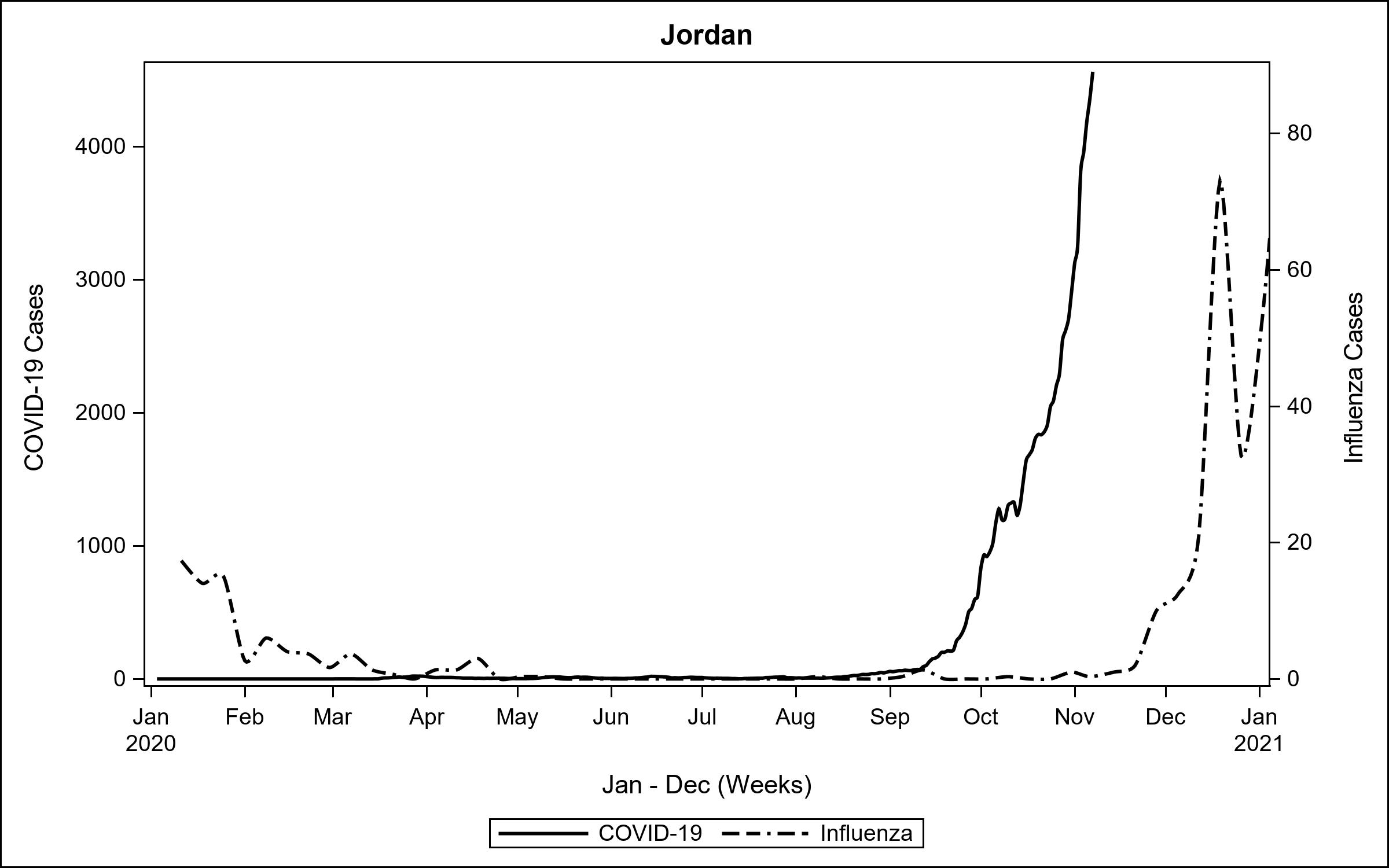

Supplement: Multimedia Appendix 4 [file publichealth_v7i3e24696_app4.zip › Country comparisons_all/Jordan1.jpeg]

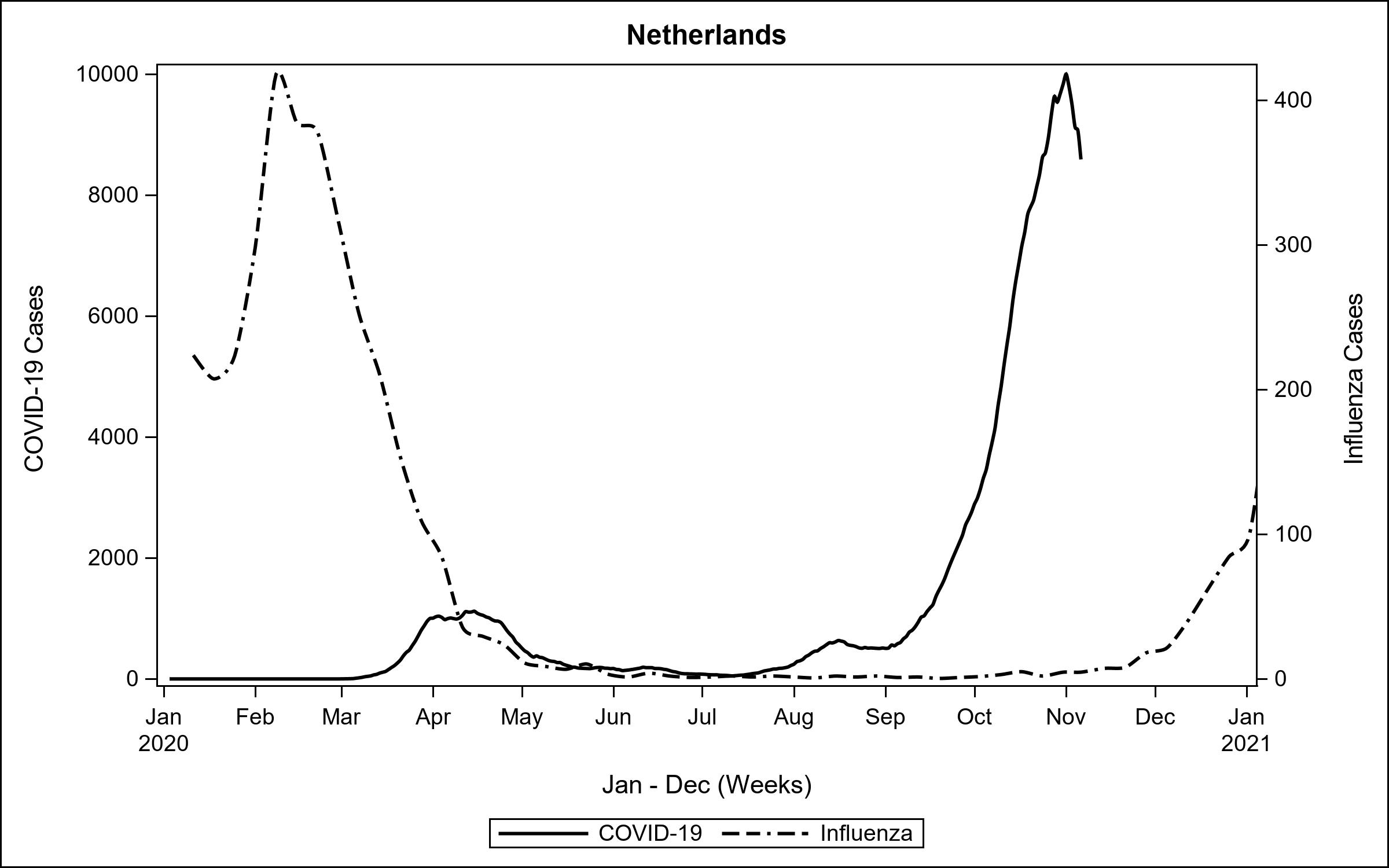

Supplement: Multimedia Appendix 4 [file publichealth_v7i3e24696_app4.zip › Country comparisons_all/Netherlands1.jpeg]

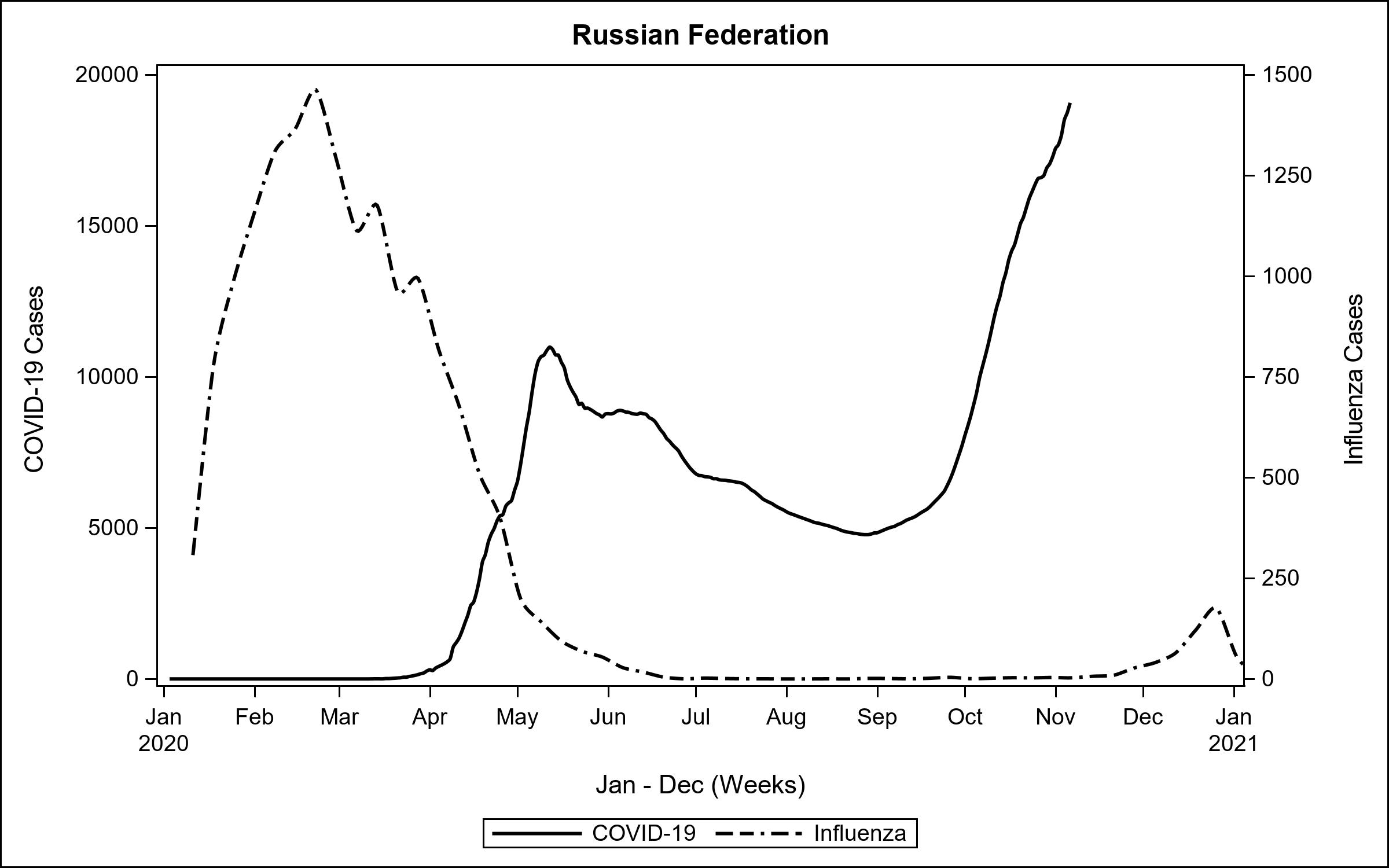

Supplement: Multimedia Appendix 4 [file publichealth_v7i3e24696_app4.zip › Country comparisons_all/Russian Federation1.jpeg]

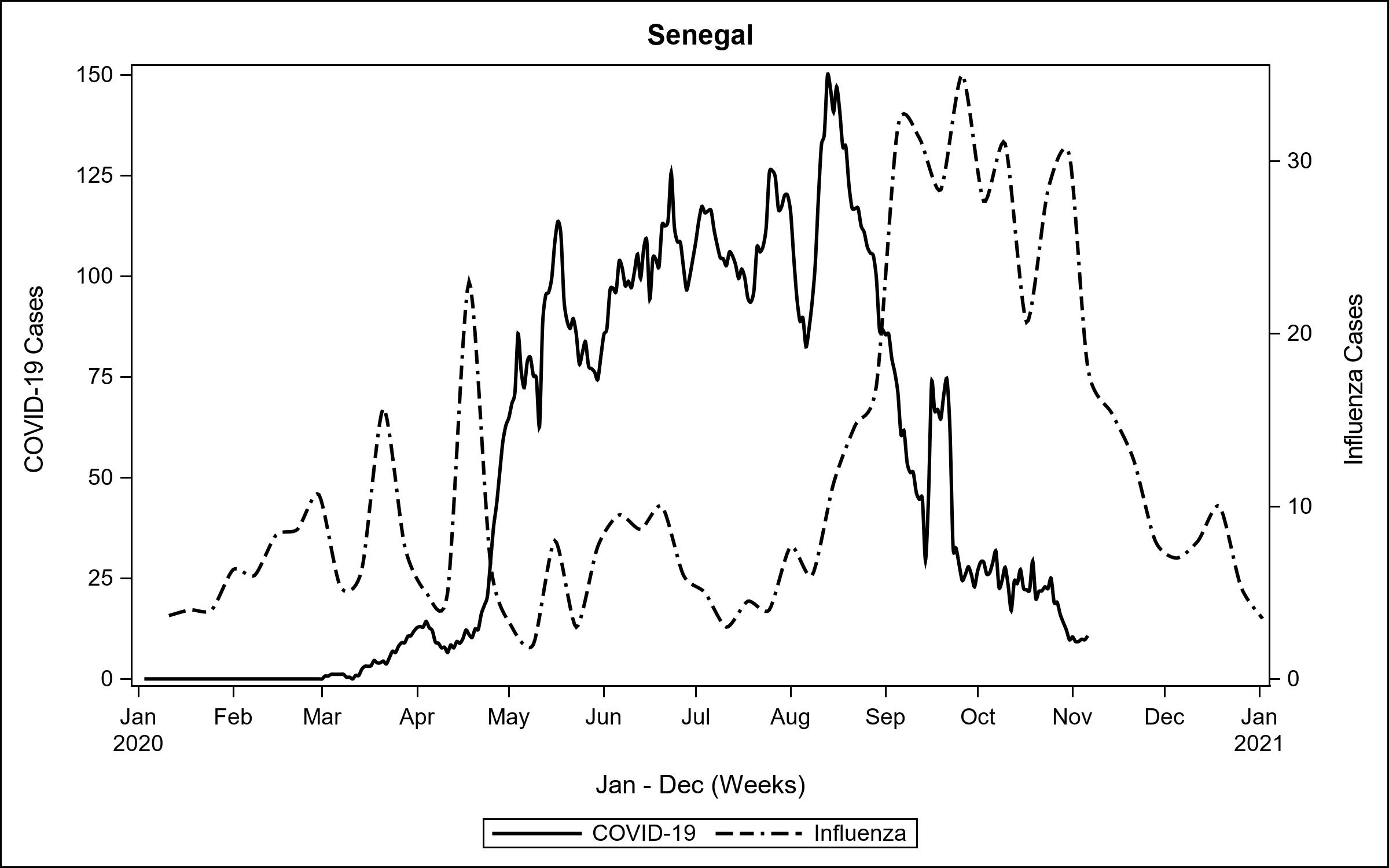

Supplement: Multimedia Appendix 4 [file publichealth_v7i3e24696_app4.zip › Country comparisons_all/Senegal1.jpeg]

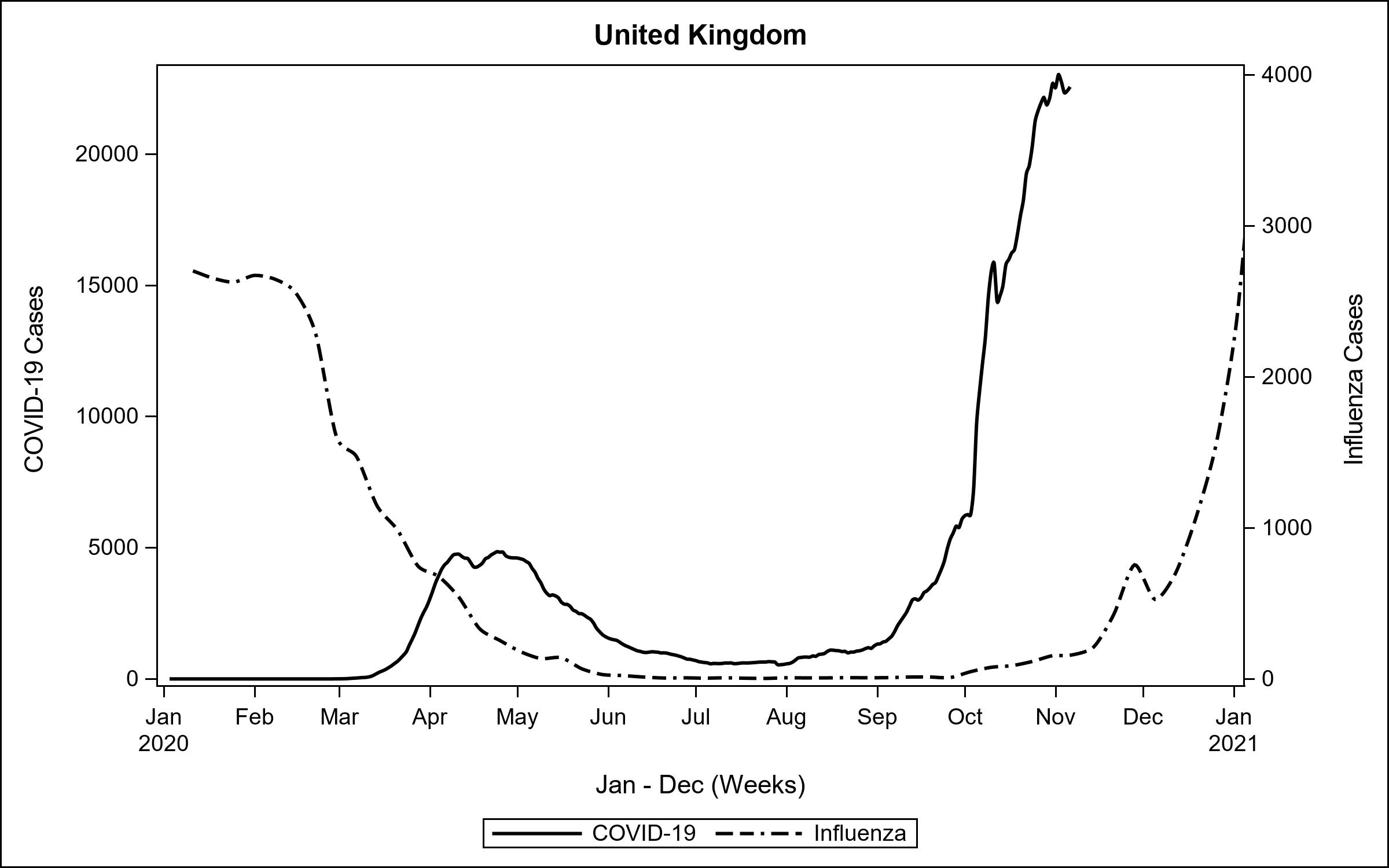

Supplement: Multimedia Appendix 4 [file publichealth_v7i3e24696_app4.zip › Country comparisons_all/United Kingdom1.jpeg]

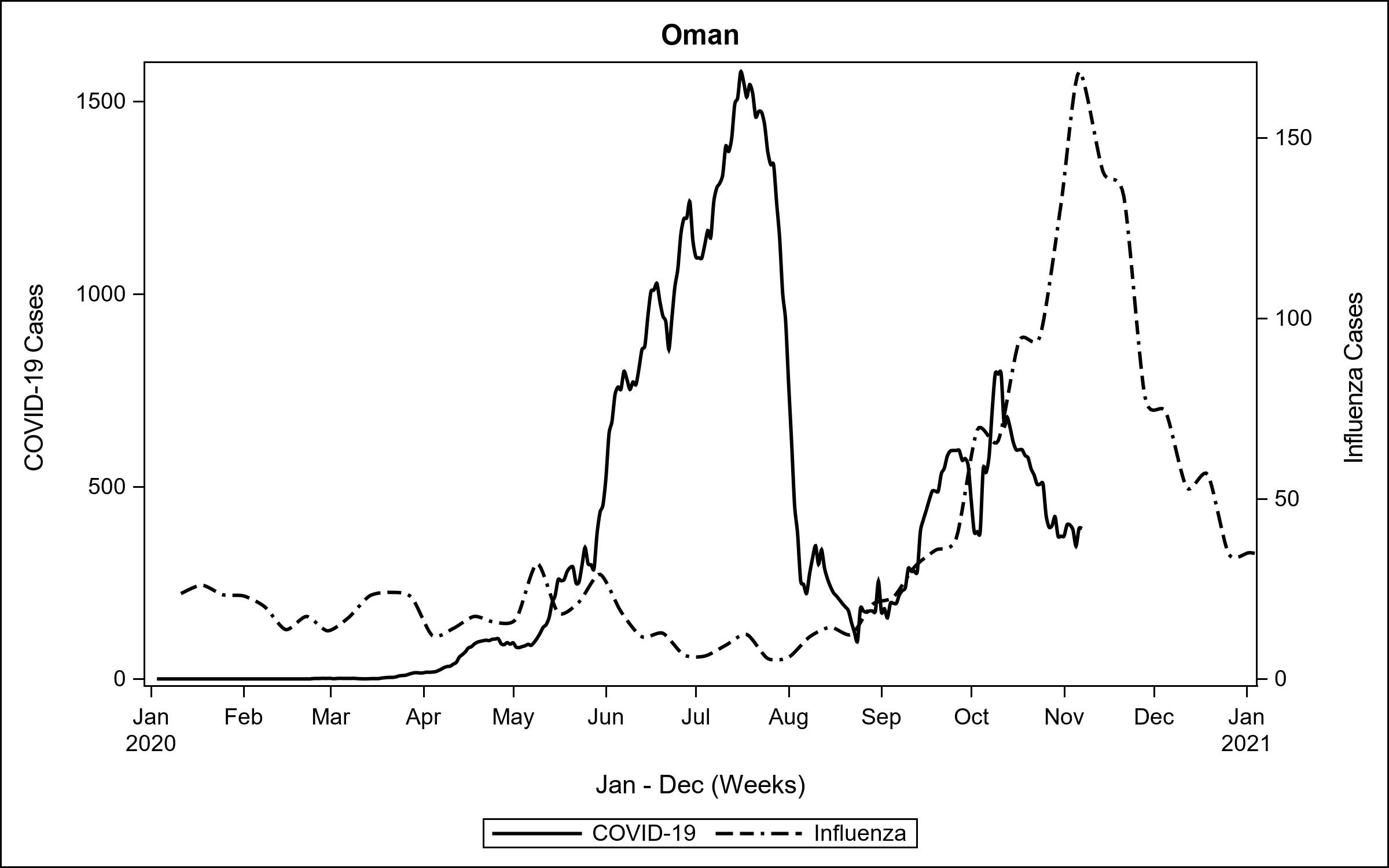

Supplement: Multimedia Appendix 4 [file publichealth_v7i3e24696_app4.zip › Country comparisons_all/Oman1.jpeg]

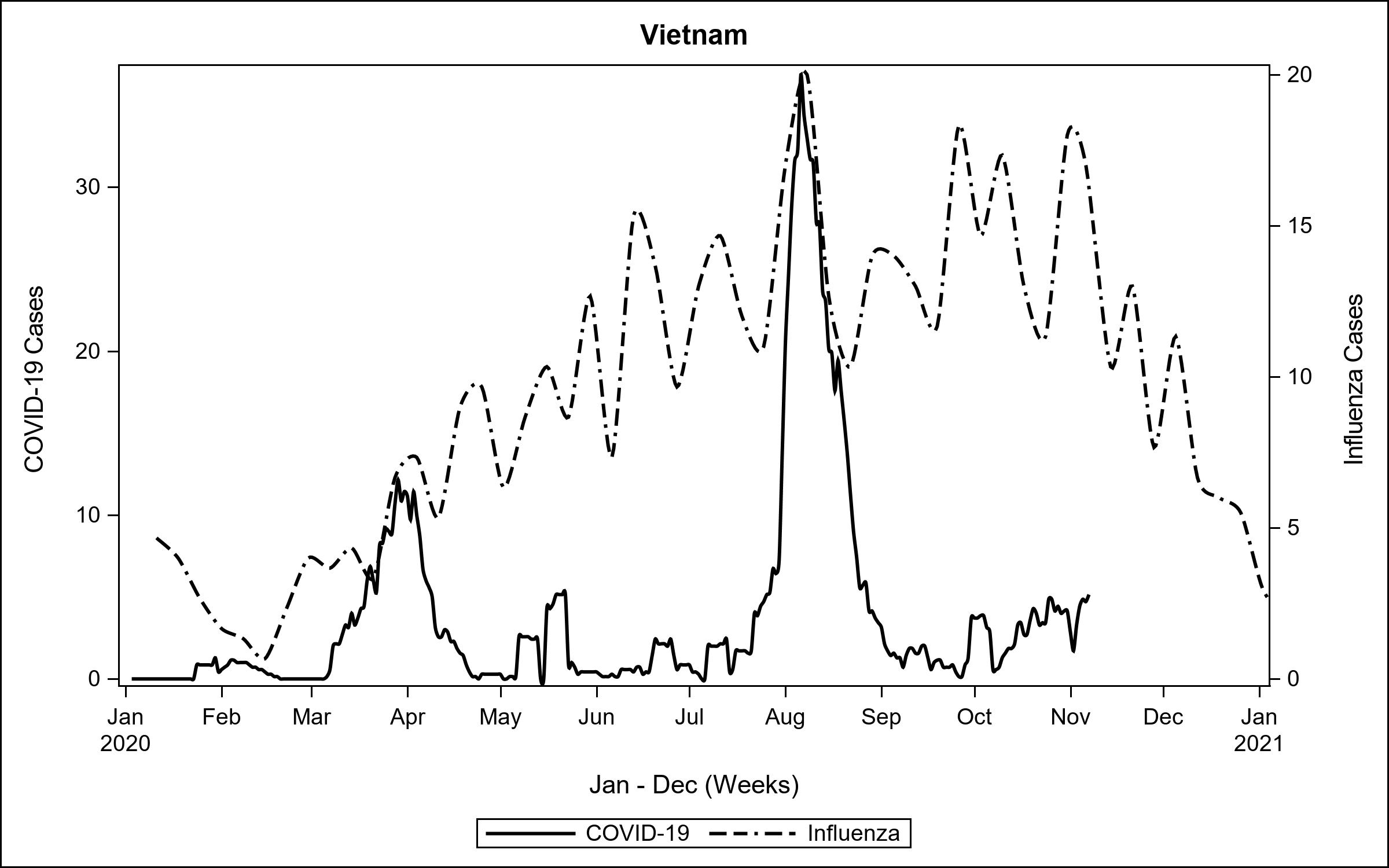

Supplement: Multimedia Appendix 4 [file publichealth_v7i3e24696_app4.zip › Country comparisons_all/Vietnam1.jpeg]

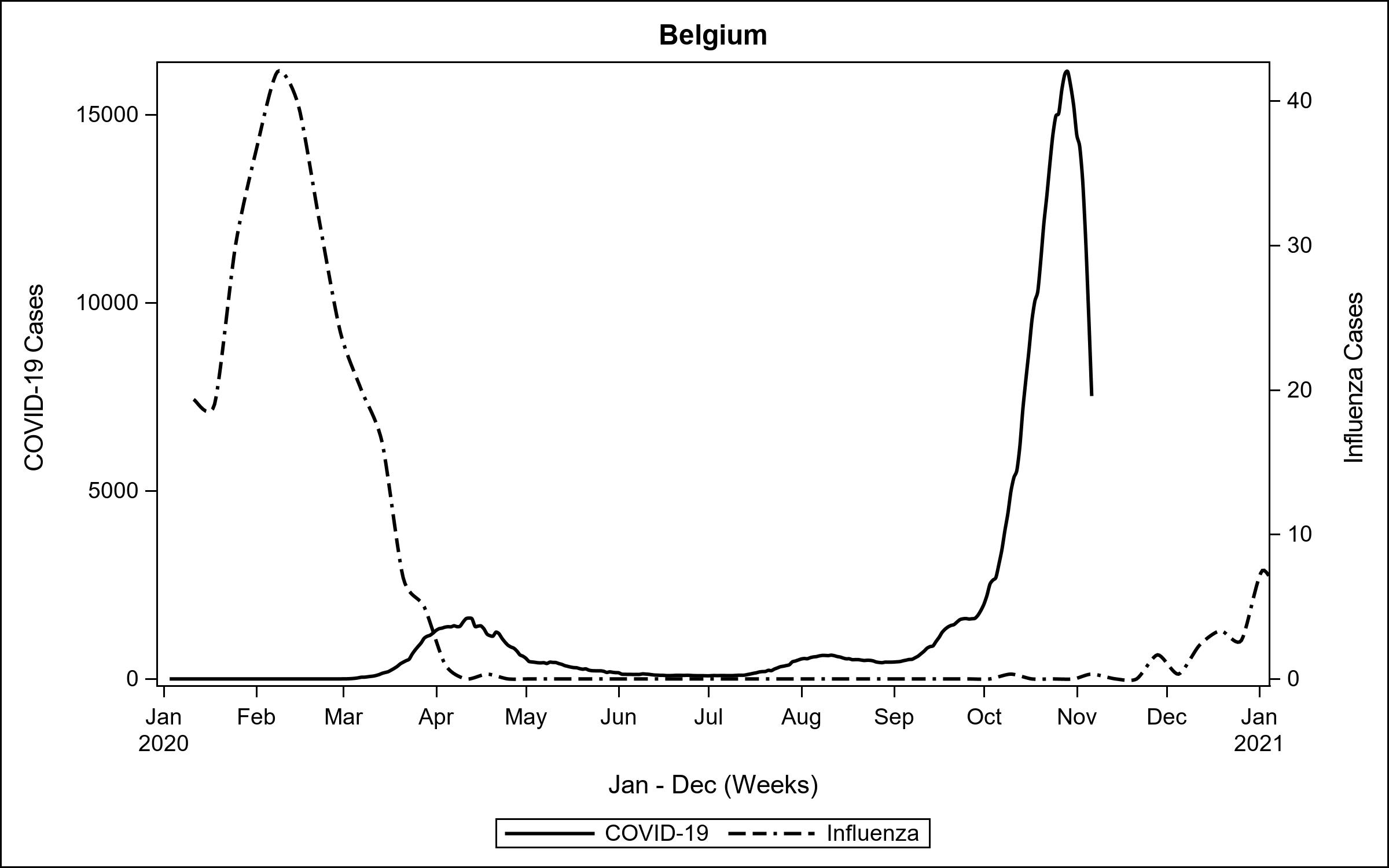

Supplement: Multimedia Appendix 4 [file publichealth_v7i3e24696_app4.zip › Country comparisons_all/Belgium1.jpeg]

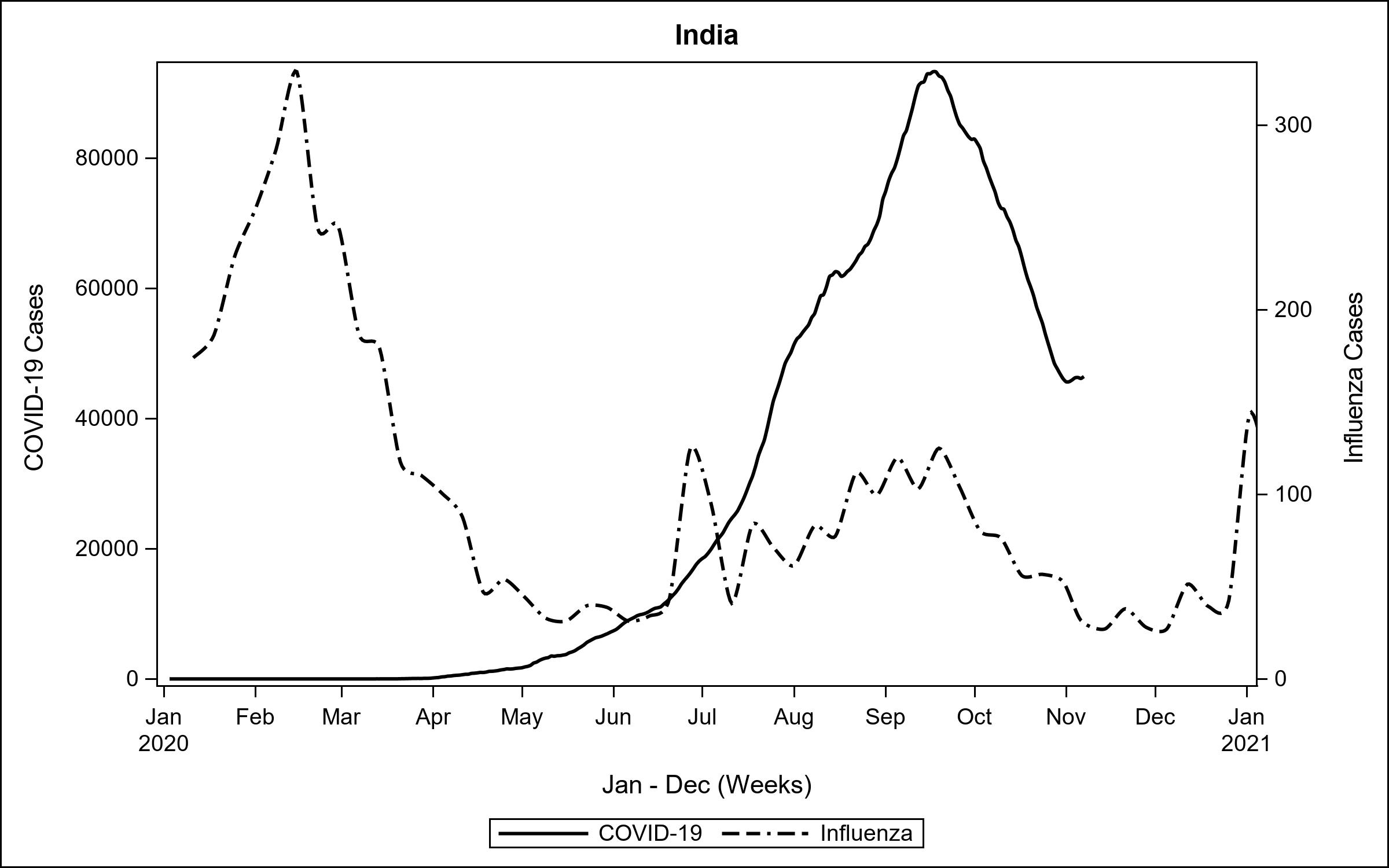

Supplement: Multimedia Appendix 4 [file publichealth_v7i3e24696_app4.zip › Country comparisons_all/India3.jpeg]

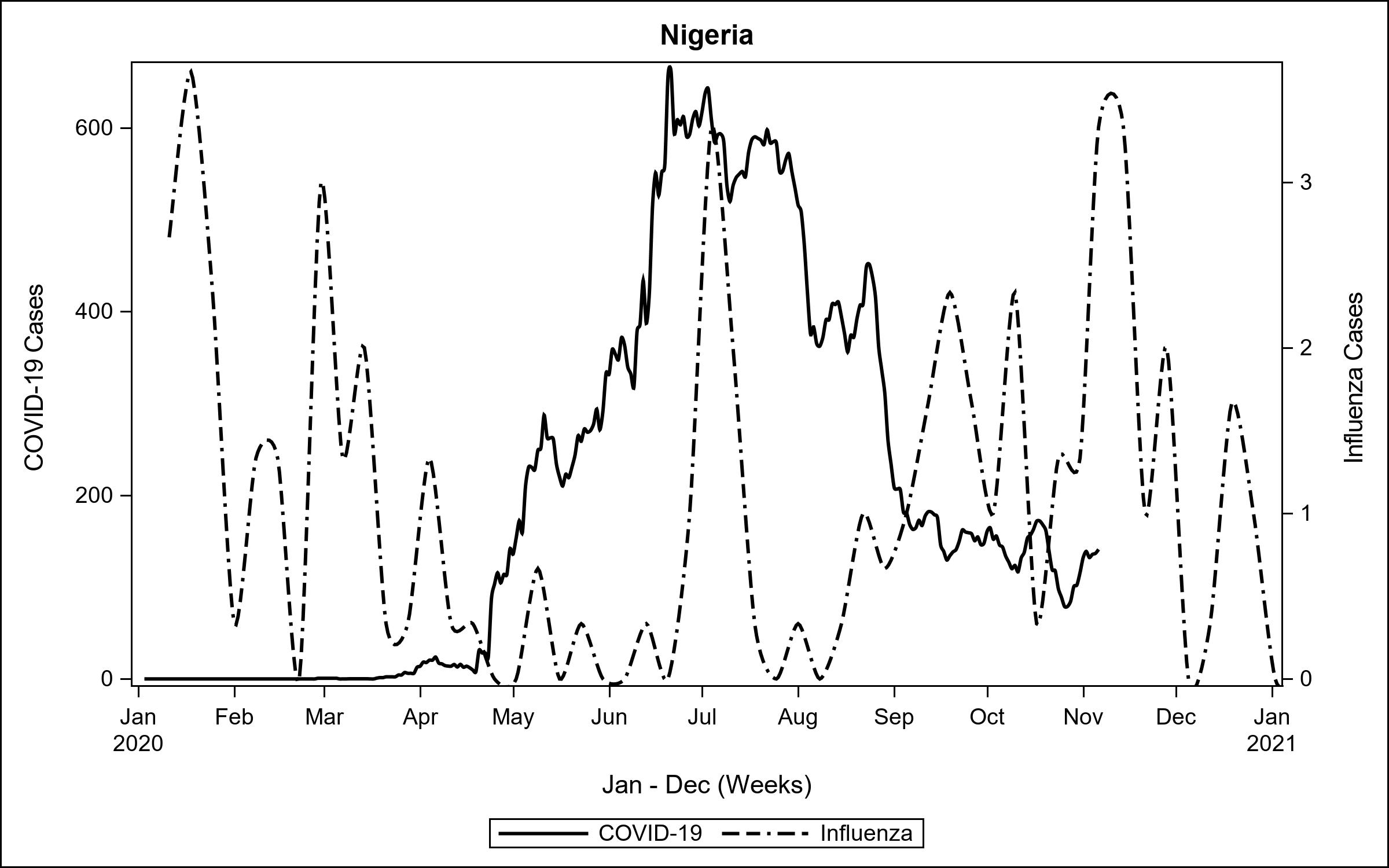

Supplement: Multimedia Appendix 4 [file publichealth_v7i3e24696_app4.zip › Country comparisons_all/Nigeria1.jpeg]

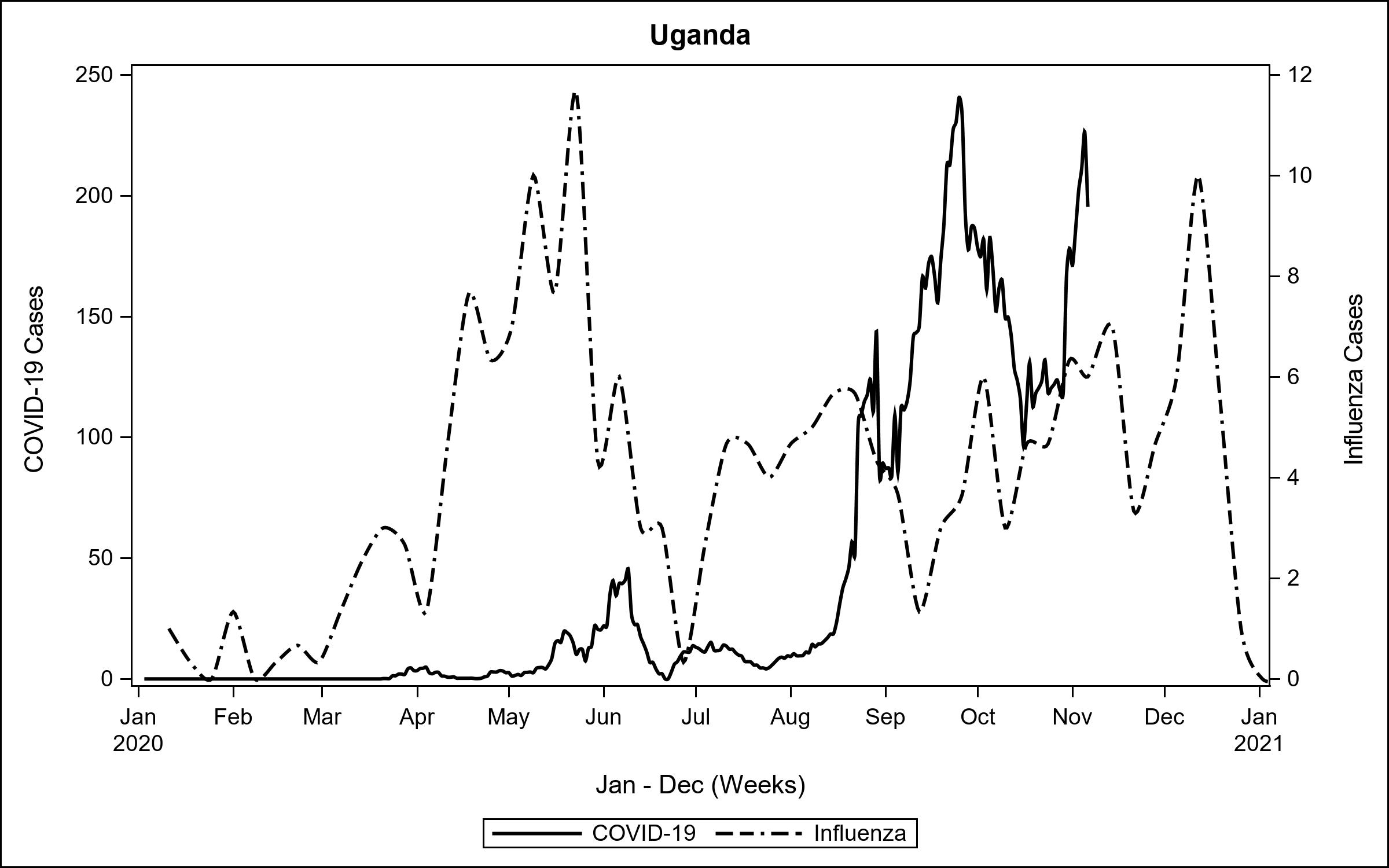

Supplement: Multimedia Appendix 4 [file publichealth_v7i3e24696_app4.zip › Country comparisons_all/Uganda1.jpeg]

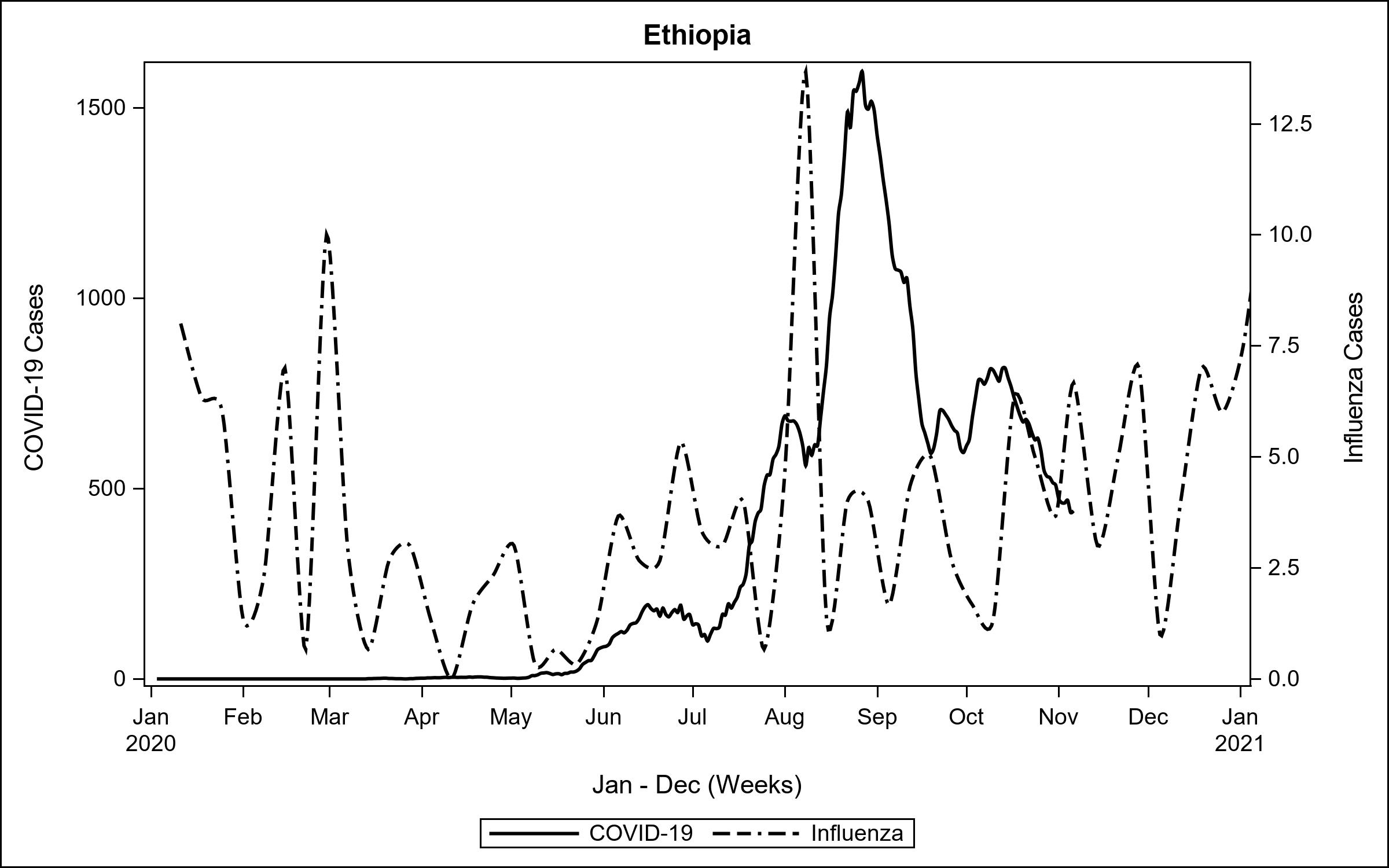

Supplement: Multimedia Appendix 4 [file publichealth_v7i3e24696_app4.zip › Country comparisons_all/Ethiopia1.jpeg]

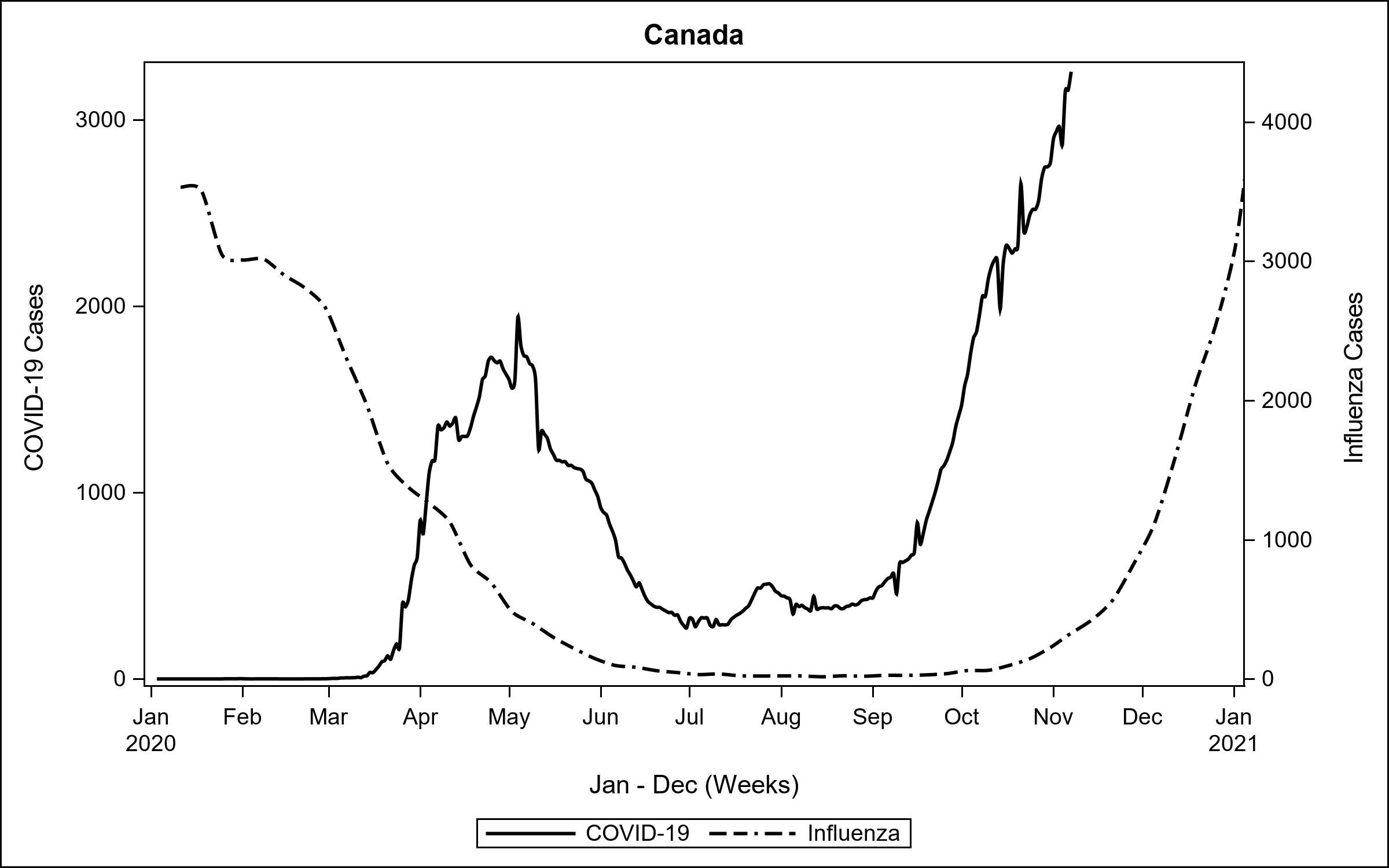

Supplement: Multimedia Appendix 4 [file publichealth_v7i3e24696_app4.zip › Country comparisons_all/Canada1.jpeg]

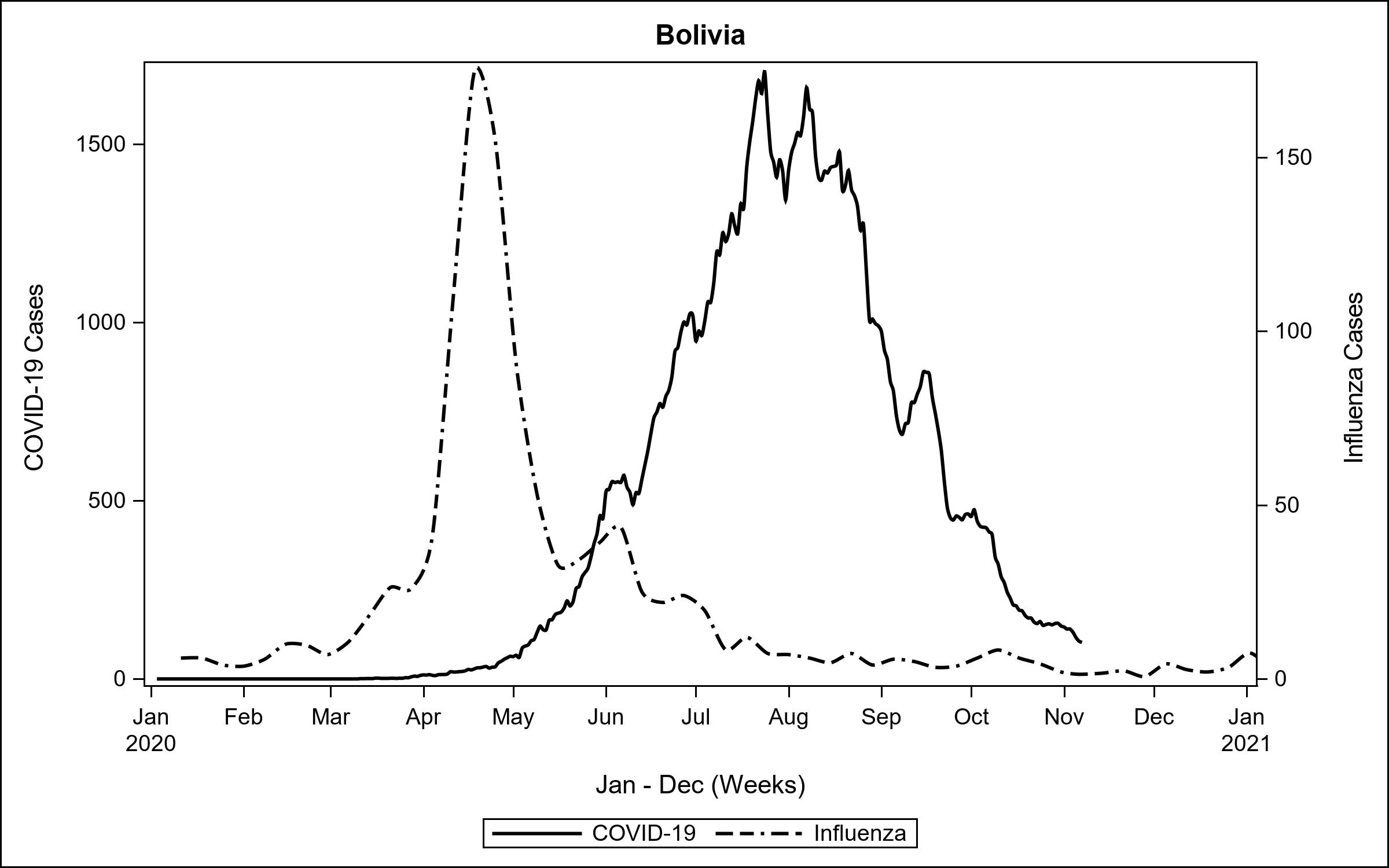

Supplement: Multimedia Appendix 4 [file publichealth_v7i3e24696_app4.zip › Country comparisons_all/Bolivia1.jpeg]

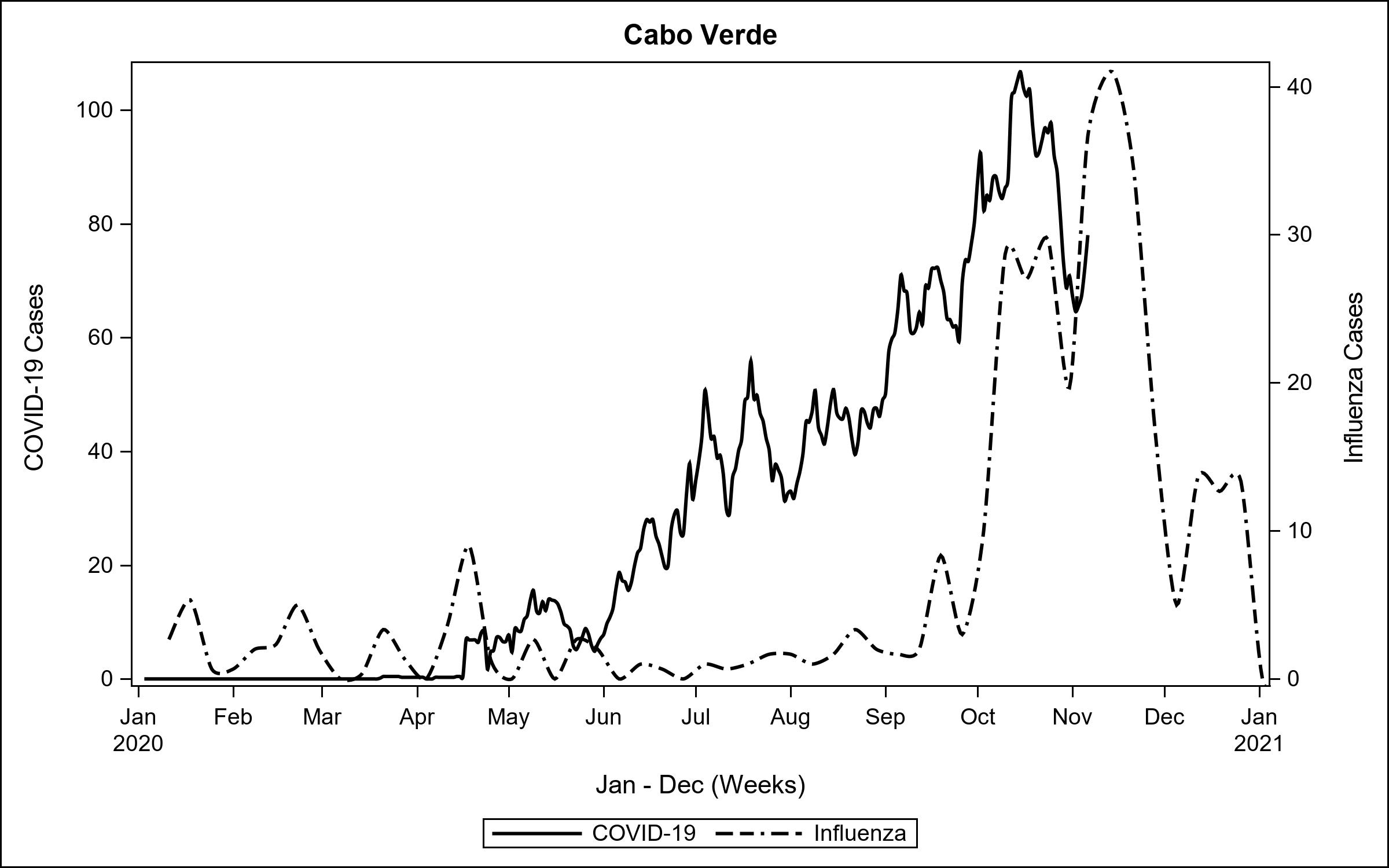

Supplement: Multimedia Appendix 4 [file publichealth_v7i3e24696_app4.zip › Country comparisons_all/Cabo Verde1.jpeg]

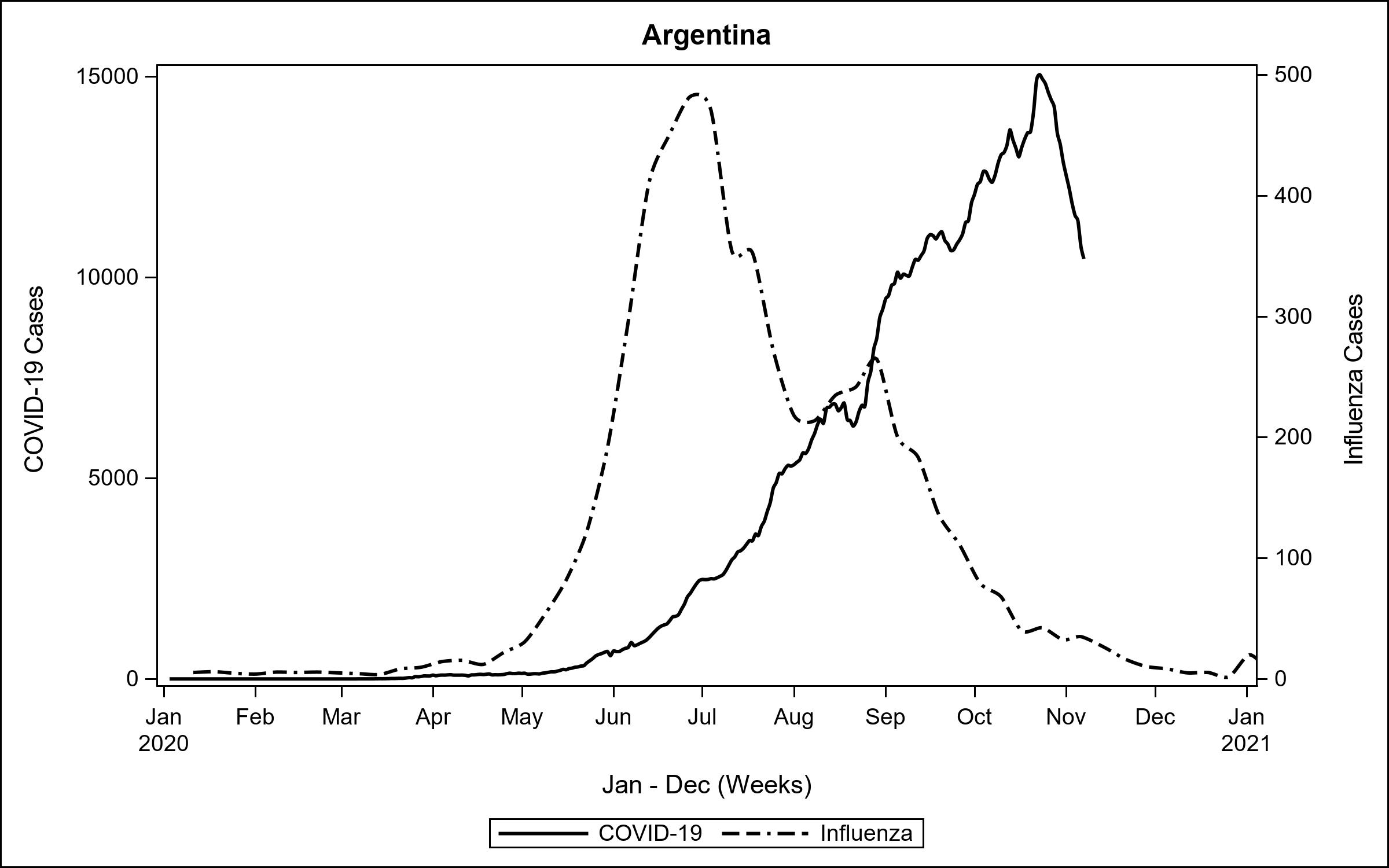

Supplement: Multimedia Appendix 4 [file publichealth_v7i3e24696_app4.zip › Country comparisons_all/Argentina1.jpeg]

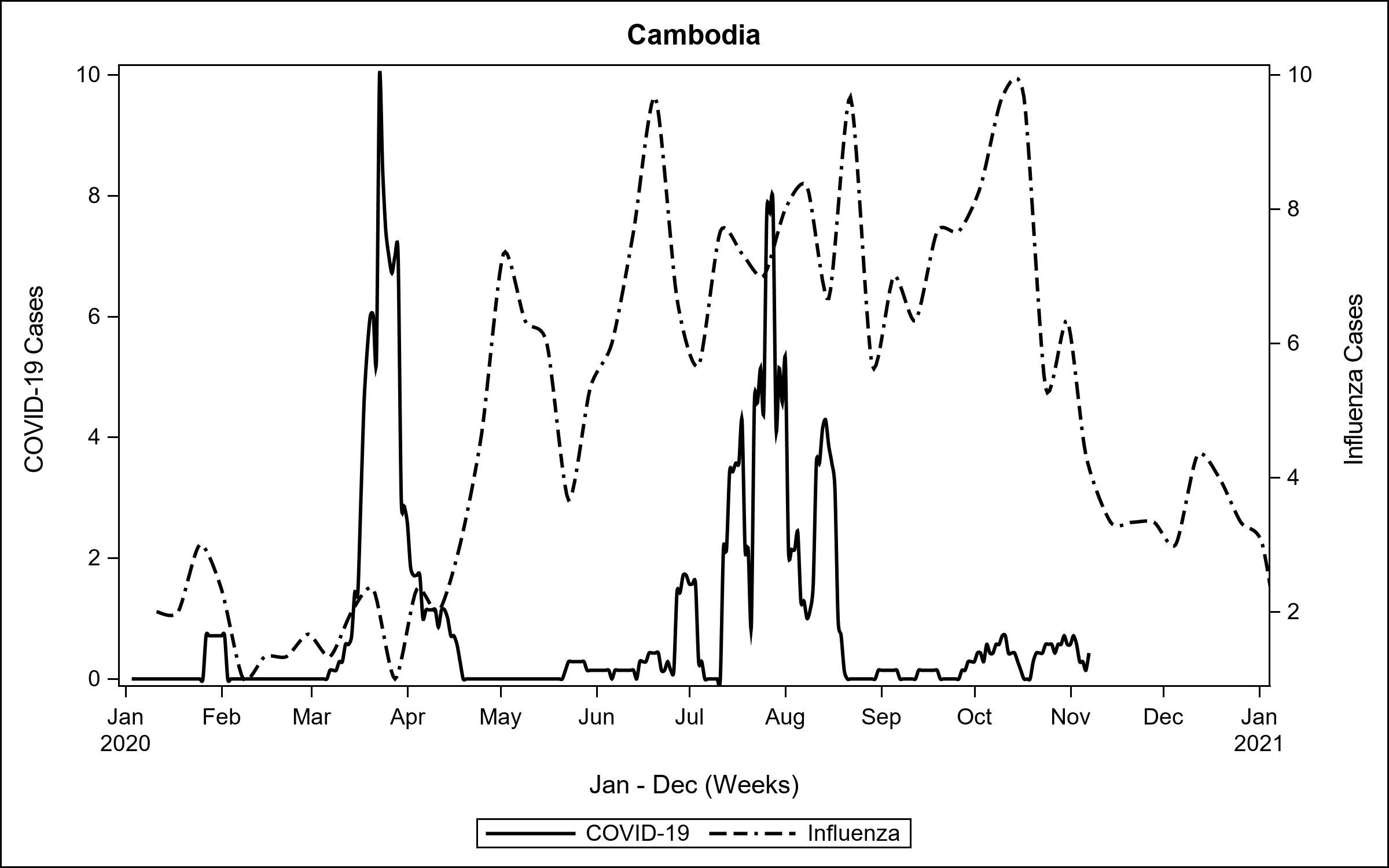

Supplement: Multimedia Appendix 4 [file publichealth_v7i3e24696_app4.zip › Country comparisons_all/Cambodia1.jpeg]

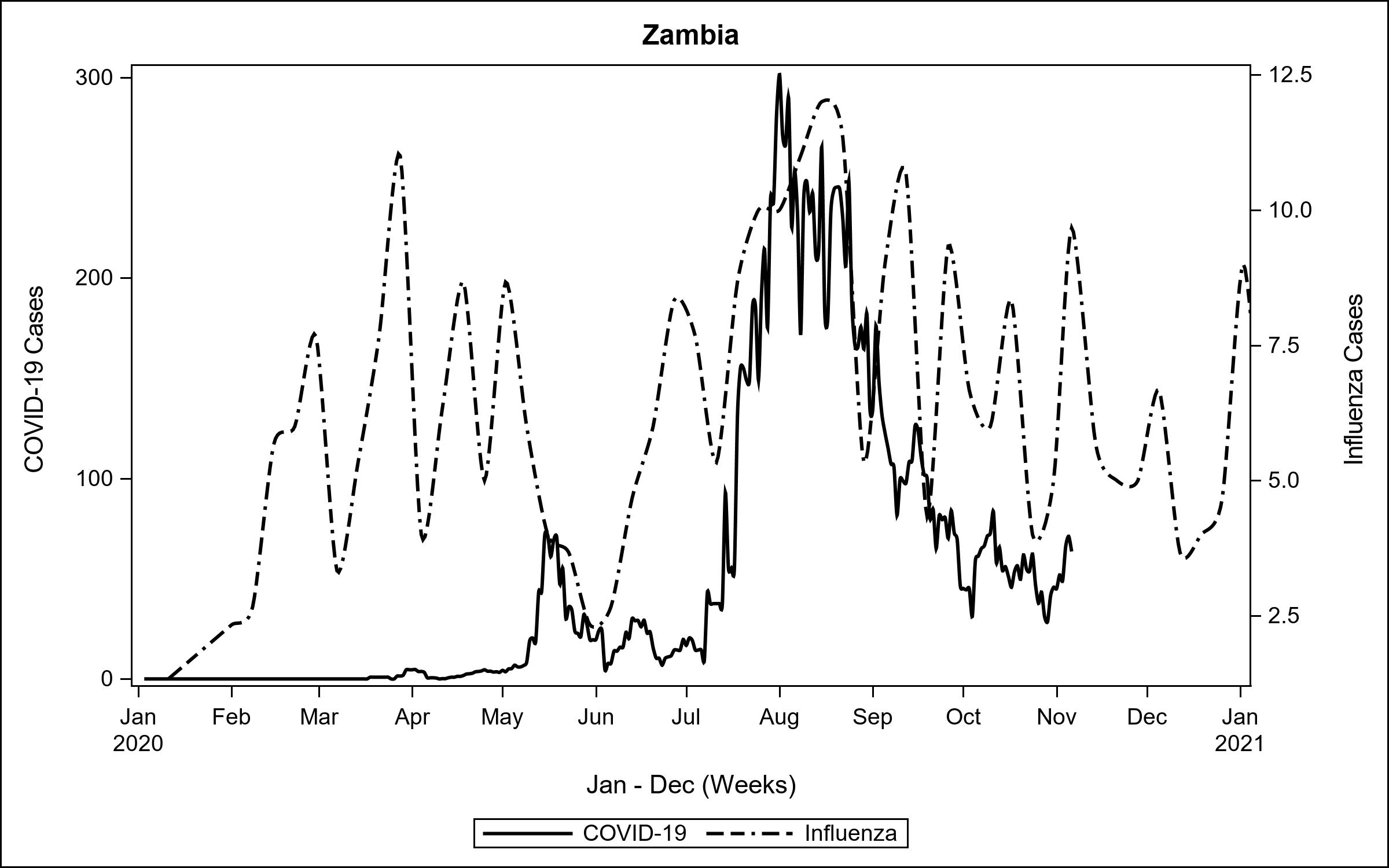

Supplement: Multimedia Appendix 4 [file publichealth_v7i3e24696_app4.zip › Country comparisons_all/Zambia1.jpeg]

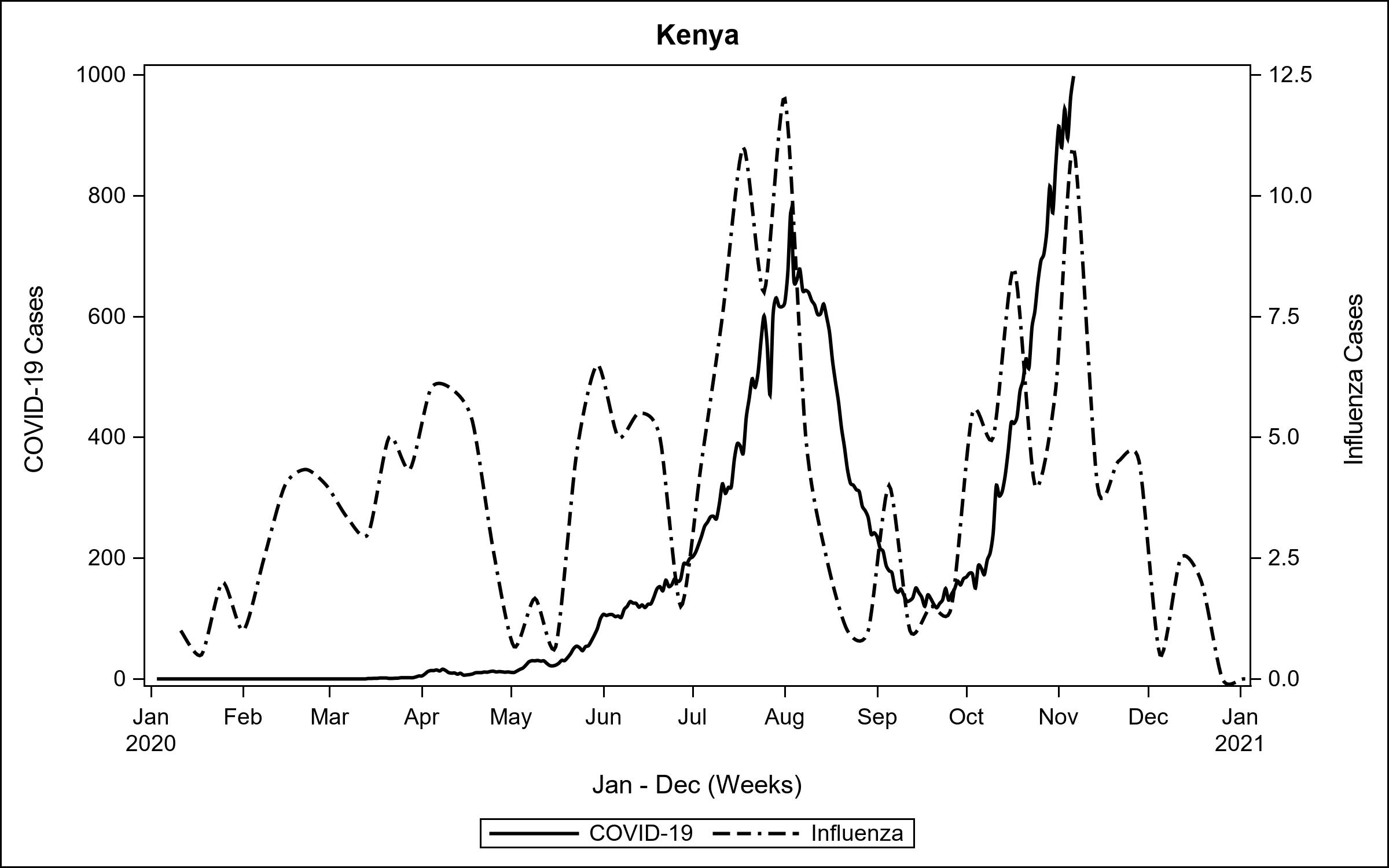

Supplement: Multimedia Appendix 4 [file publichealth_v7i3e24696_app4.zip › Country comparisons_all/Kenya1.jpeg]

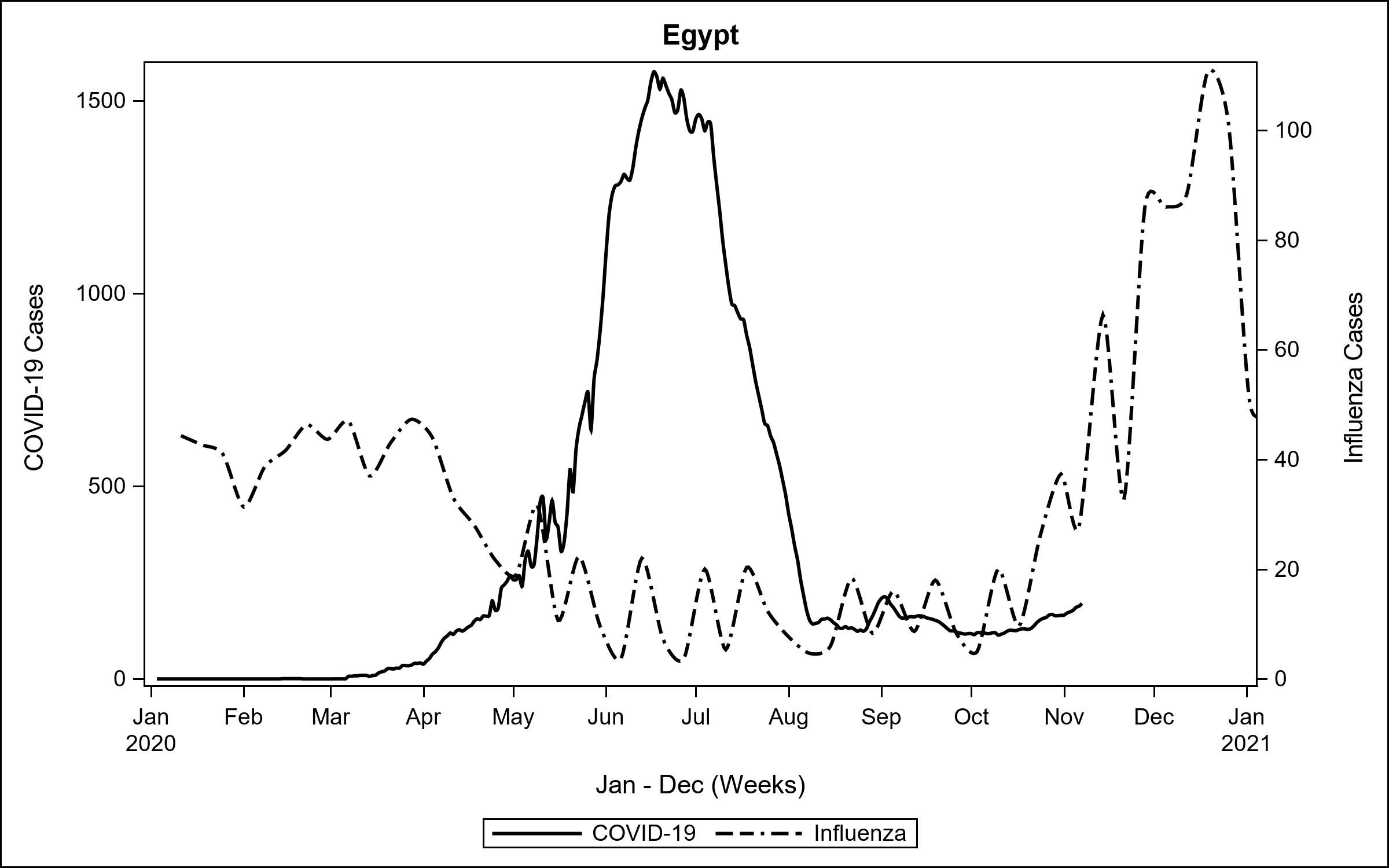

Supplement: Multimedia Appendix 4 [file publichealth_v7i3e24696_app4.zip › Country comparisons_all/Egypt1.jpeg]

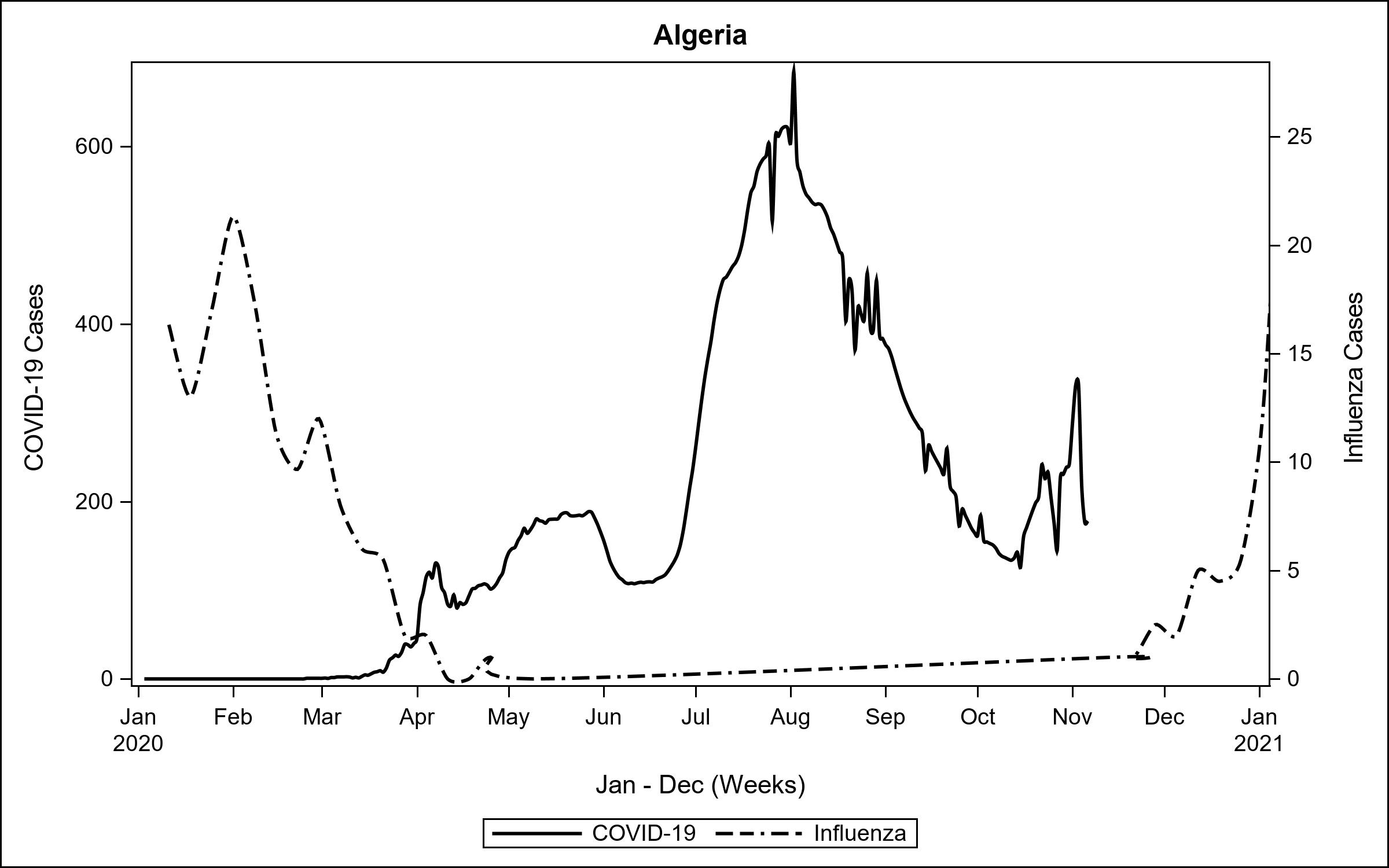

Supplement: Multimedia Appendix 4 [file publichealth_v7i3e24696_app4.zip › Country comparisons_all/Algeria1.jpeg]

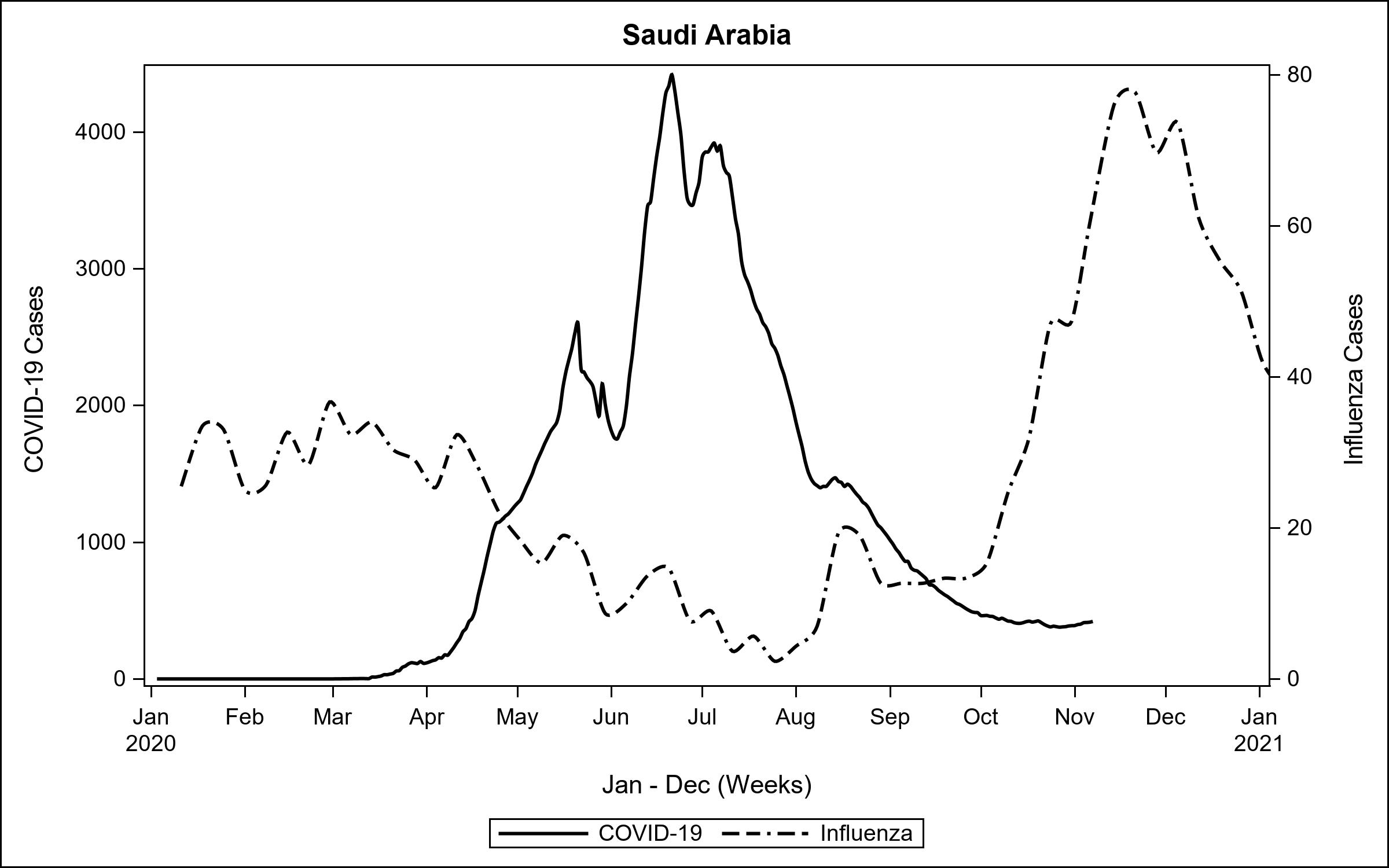

Supplement: Multimedia Appendix 4 [file publichealth_v7i3e24696_app4.zip › Country comparisons_all/Saudi Arabia1.jpeg]

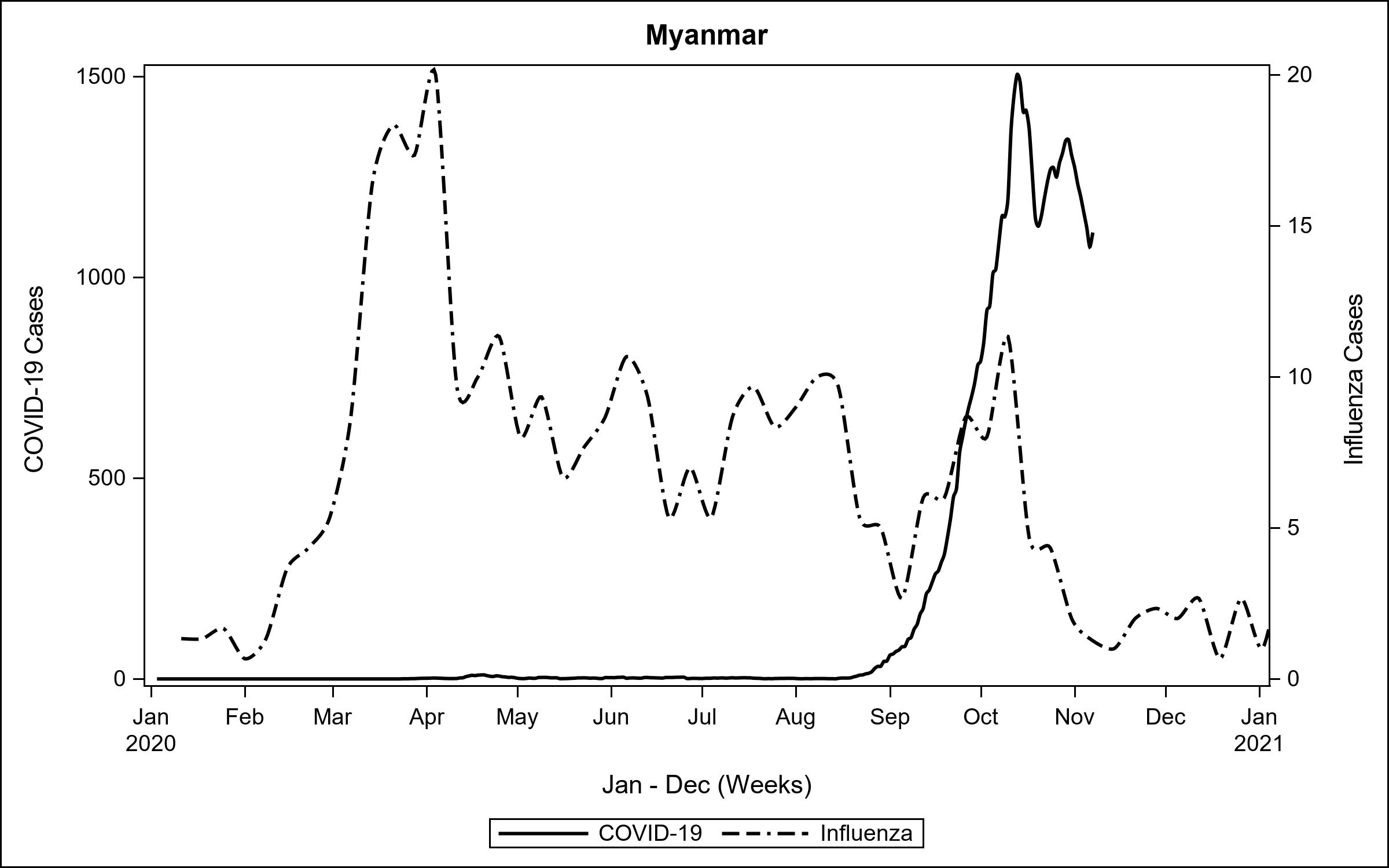

Supplement: Multimedia Appendix 4 [file publichealth_v7i3e24696_app4.zip › Country comparisons_all/Myanmar1.jpeg]

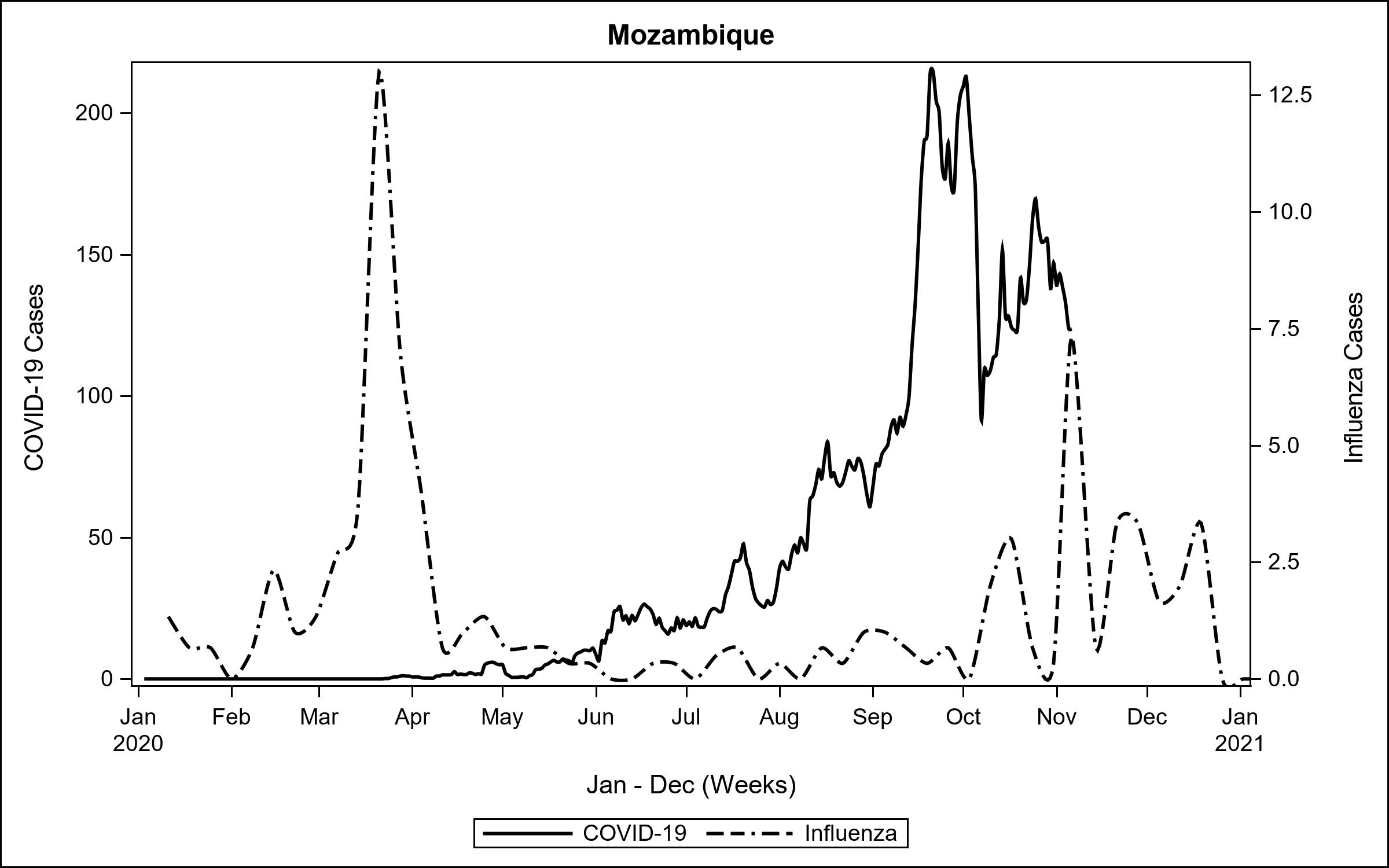

Supplement: Multimedia Appendix 4 [file publichealth_v7i3e24696_app4.zip › Country comparisons_all/Mozambique1.jpeg]

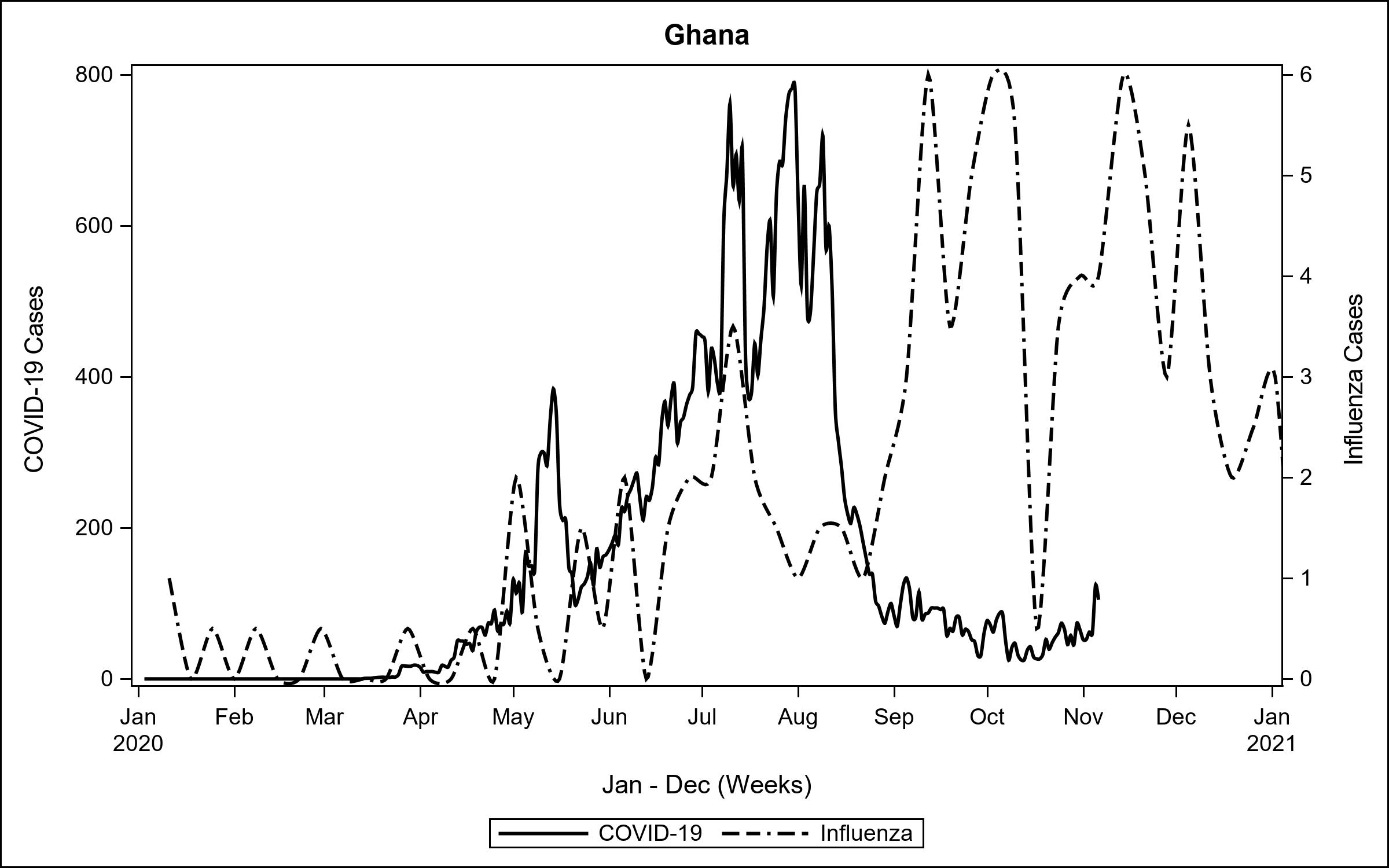

Supplement: Multimedia Appendix 4 [file publichealth_v7i3e24696_app4.zip › Country comparisons_all/Ghana1.jpeg]

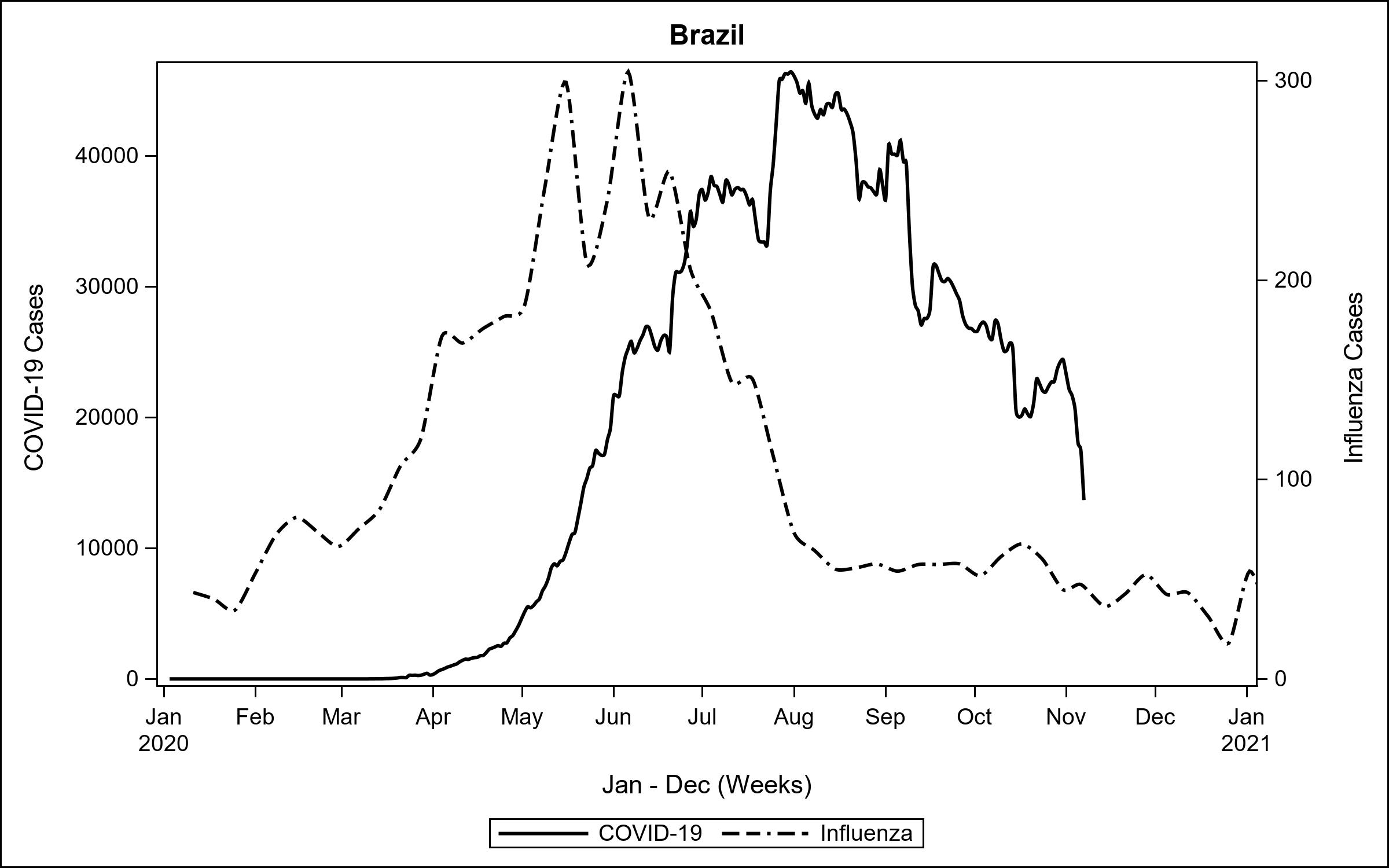

Supplement: Multimedia Appendix 4 [file publichealth_v7i3e24696_app4.zip › Country comparisons_all/Brazil3.jpeg]

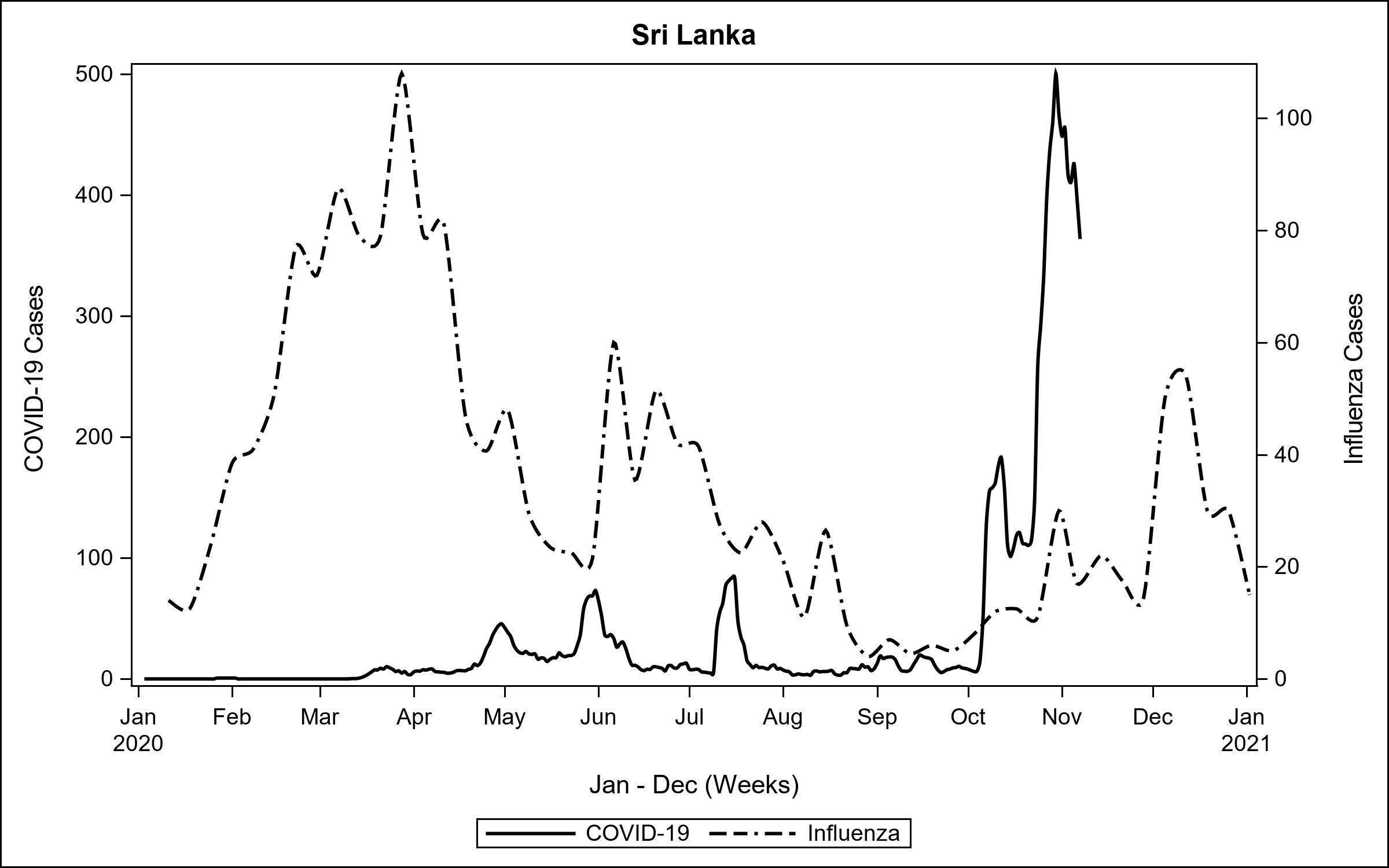

Supplement: Multimedia Appendix 4 [file publichealth_v7i3e24696_app4.zip › Country comparisons_all/Sri Lanka1.jpeg]

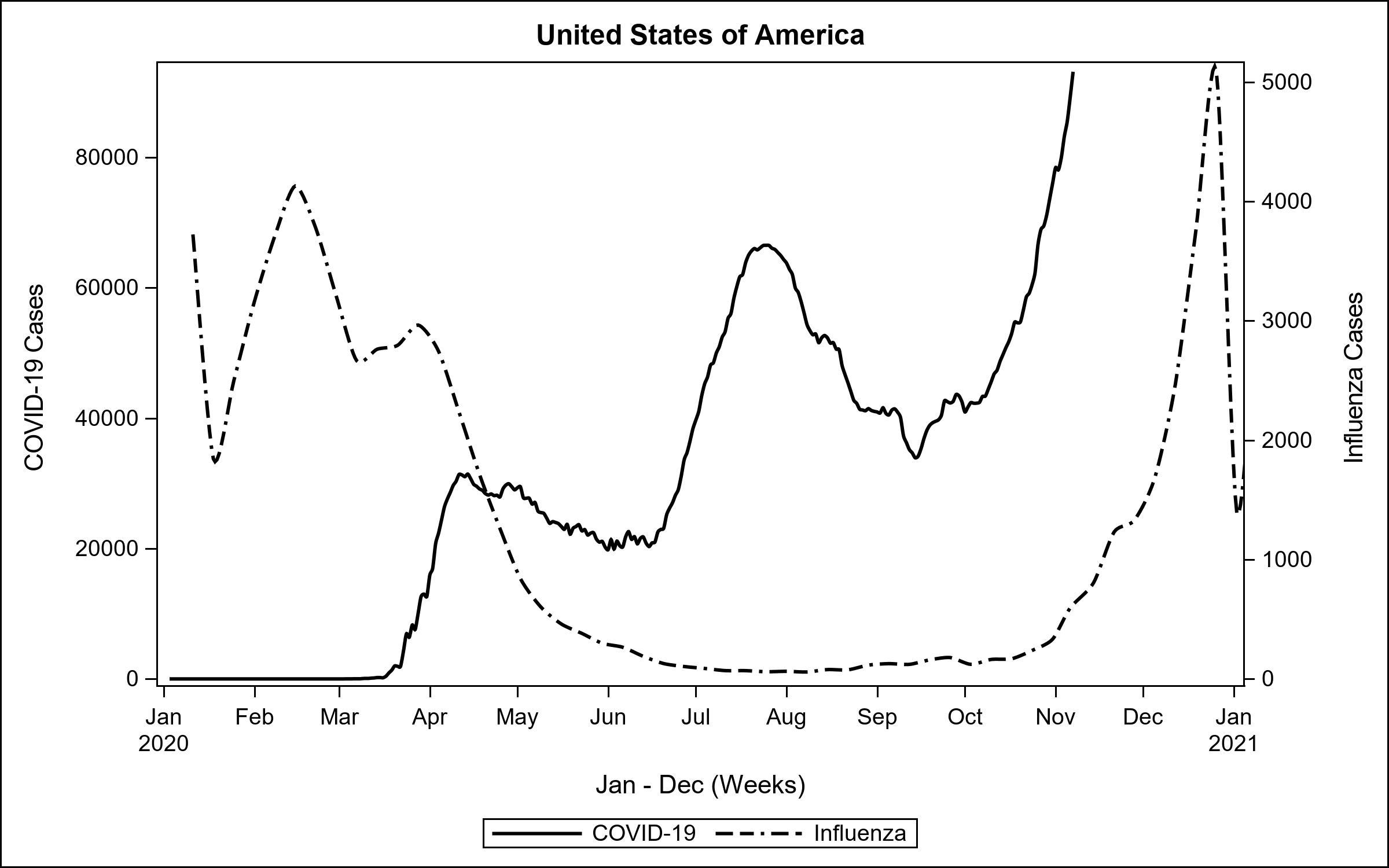

Supplement: Multimedia Appendix 4 [file publichealth_v7i3e24696_app4.zip › Country comparisons_all/United States of America3.jpeg]

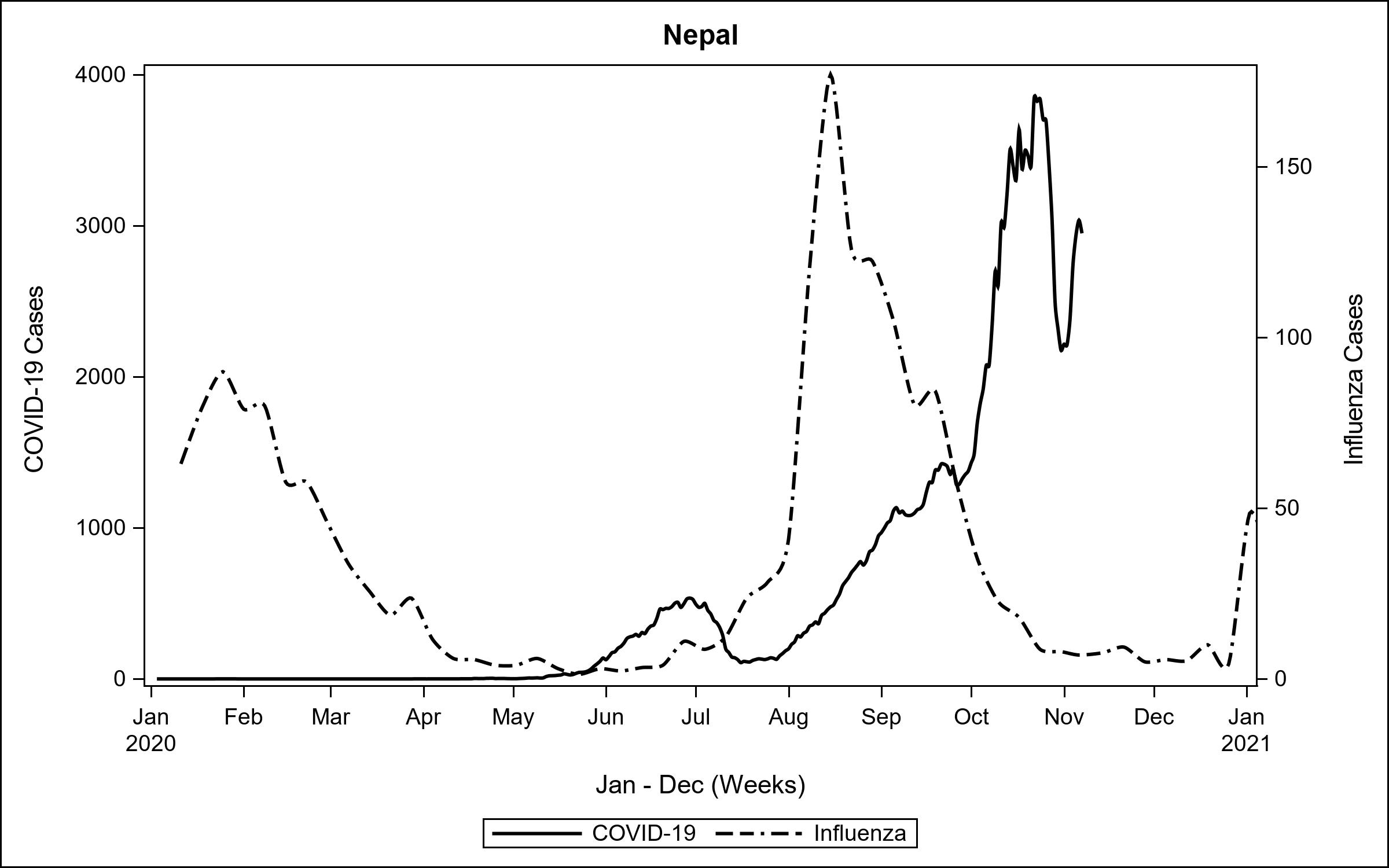

Supplement: Multimedia Appendix 4 [file publichealth_v7i3e24696_app4.zip › Country comparisons_all/Nepal1.jpeg]

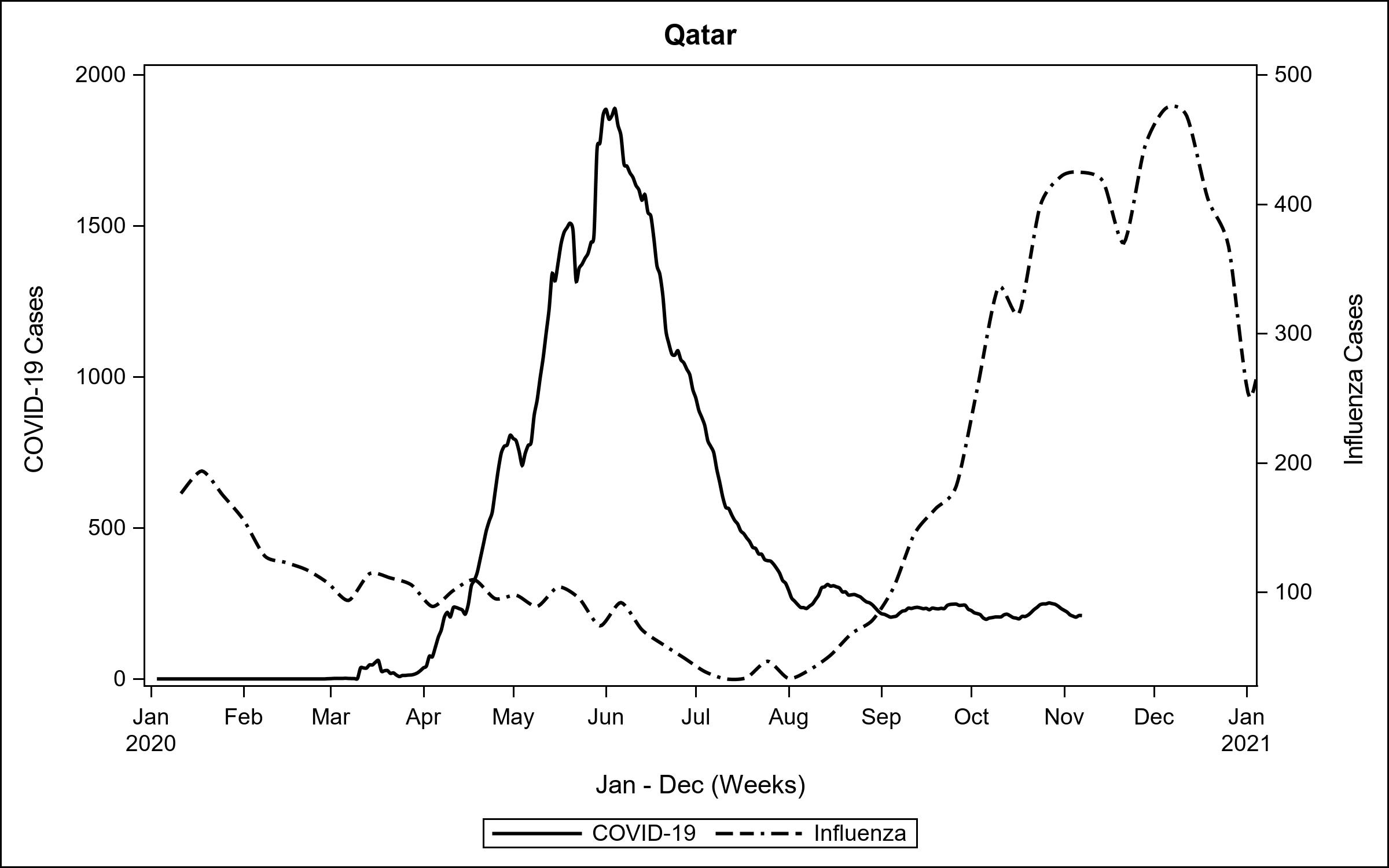

Supplement: Multimedia Appendix 4 [file publichealth_v7i3e24696_app4.zip › Country comparisons_all/Qatar1.jpeg]

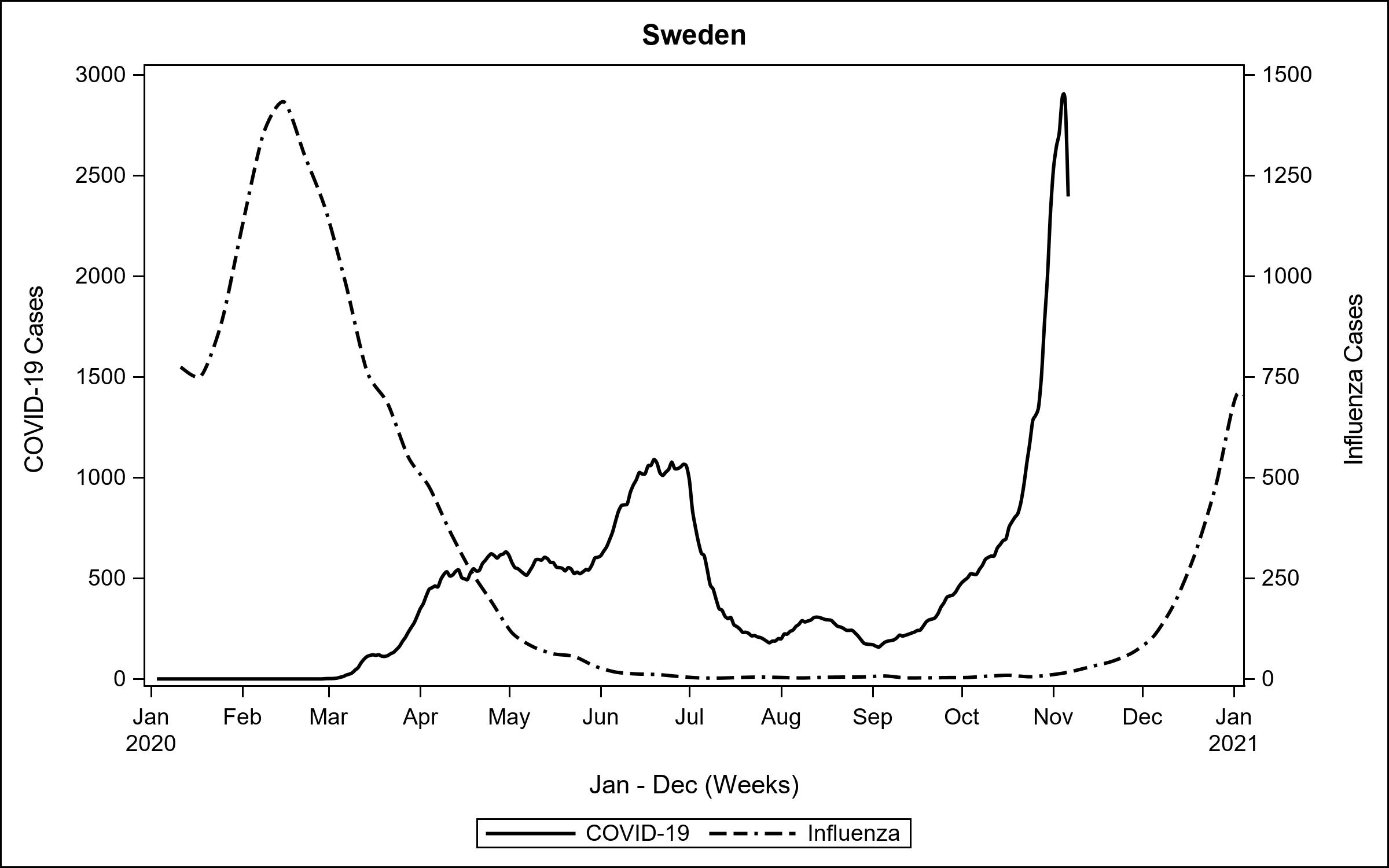

Supplement: Multimedia Appendix 4 [file publichealth_v7i3e24696_app4.zip › Country comparisons_all/Sweden3.jpeg]

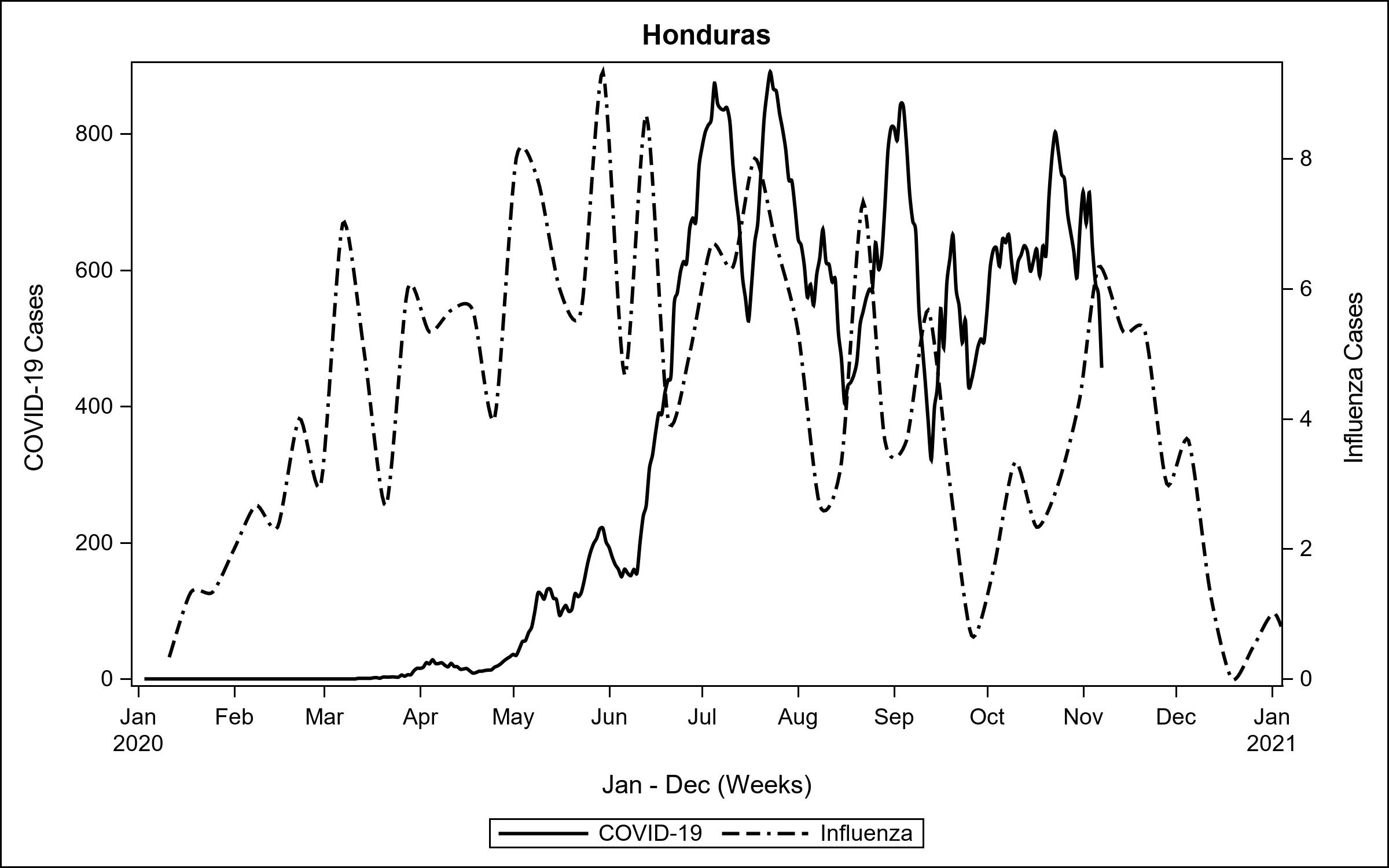

Supplement: Multimedia Appendix 4 [file publichealth_v7i3e24696_app4.zip › Country comparisons_all/Honduras1.jpeg]

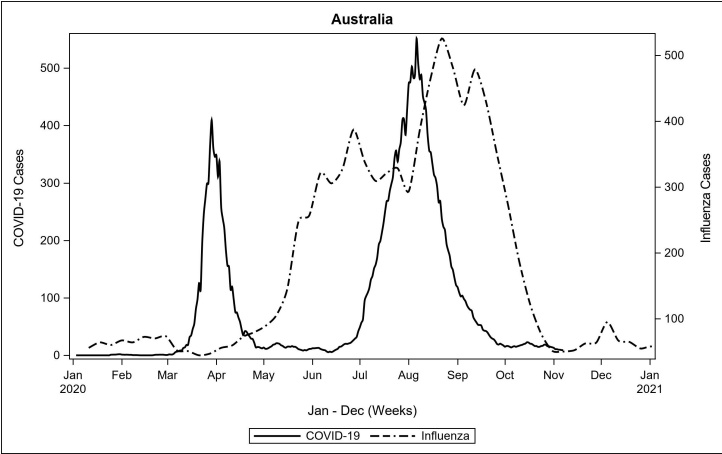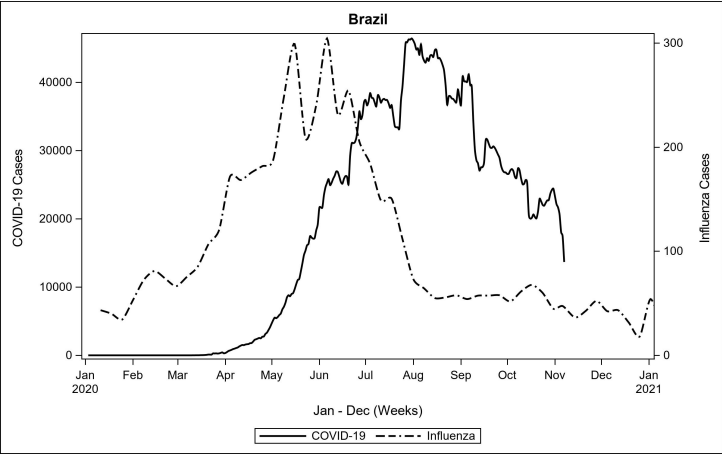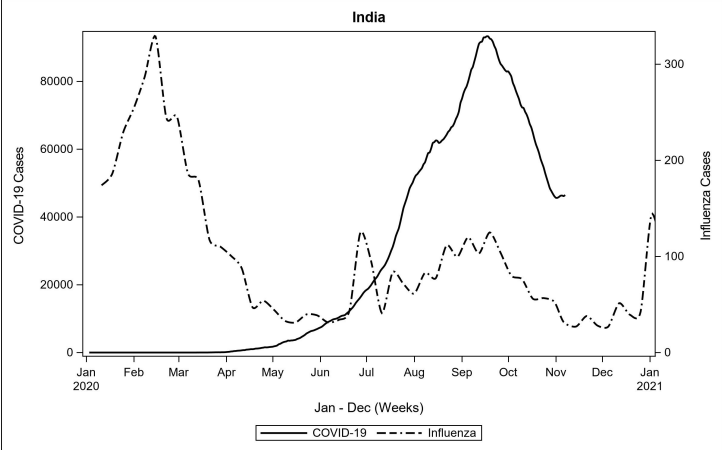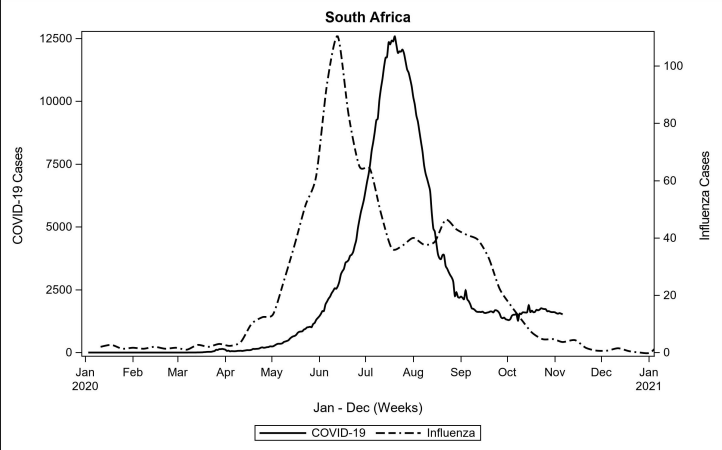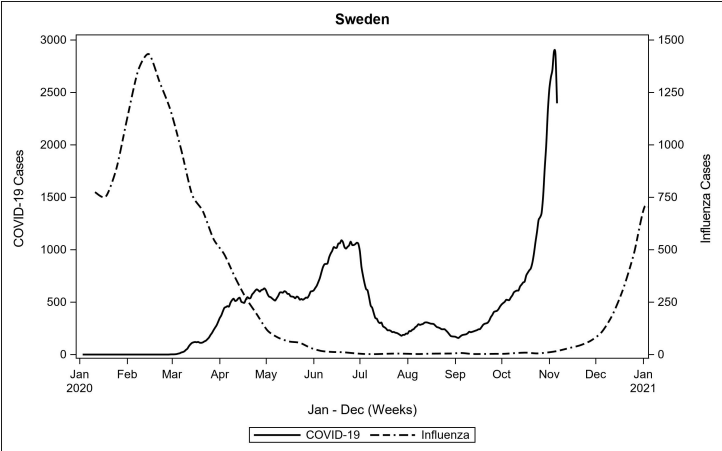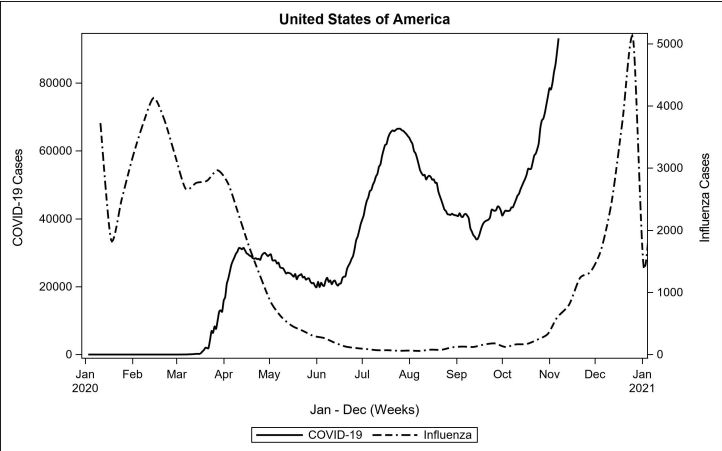

Supplement: Multimedia Appendix 5 [file publichealth_v7i3e24696_app5.pdf]
